# Skeletal muscle: A review of molecular structure and function, in health and disease

**DOI:** 10.1002/wsbm.1462

**Published:** 2019-08-13

**Authors:** Kavitha Mukund, Shankar Subramaniam

**Affiliations:** ^1^ Department of Bioengineering University of California San Diego California; ^2^ Department of Bioengineering, Bioinformatics & Systems Biology University of California San Diego California; ^3^ Department of Computer Science and Engineering University of California San Diego California; ^4^ Department of Cellular and Molecular Medicine and Nanoengineering University of California San Diego California

**Keywords:** molecular mechanisms, molecular structure, muscle health and disease, muscle physiology, skeletal muscle

## Abstract

Decades of research in skeletal muscle physiology have provided multiscale insights into the structural and functional complexity of this important anatomical tissue, designed to accomplish the task of generating contraction, force and movement. Skeletal muscle can be viewed as a biomechanical device with various interacting components including the autonomic nerves for impulse transmission, vasculature for efficient oxygenation, and embedded regulatory and metabolic machinery for maintaining cellular homeostasis. The “omics” revolution has propelled a new era in muscle research, allowing us to discern minute details of molecular cross‐talk required for effective coordination between the myriad interacting components for efficient muscle function. The objective of this review is to provide a systems‐level, comprehensive mapping the molecular mechanisms underlying skeletal muscle structure and function, in health and disease. We begin this review with a focus on molecular mechanisms underlying muscle tissue development (myogenesis), with an emphasis on satellite cells and muscle regeneration. We next review the molecular structure and mechanisms underlying the many structural components of the muscle: neuromuscular junction, sarcomere, cytoskeleton, extracellular matrix, and vasculature surrounding muscle. We highlight aberrant molecular mechanisms and their possible clinical or pathophysiological relevance. We particularly emphasize the impact of environmental stressors (inflammation and oxidative stress) in contributing to muscle pathophysiology including atrophy, hypertrophy, and fibrosis.

This article is categorized under:

Physiology > Mammalian Physiology in Health and Disease
Developmental Biology > Developmental Processes in Health and Disease
Models of Systems Properties and Processes > Cellular Models

## Graphical abstract

The current review focuses on molecular structure and function of the various components of muscle physiology. Within each component, we highlight the necessary molecular mechanisms and cross‐talk critical for defining the state of muscle health. We also highlight instances of aberrant molecular mechanisms underlying disease.

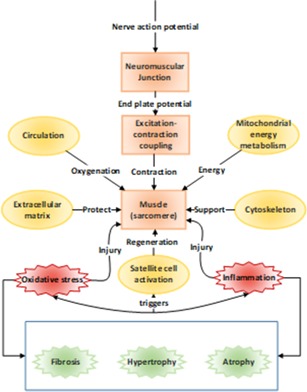

## 1 INTRODUCTION

**Figure 1:**
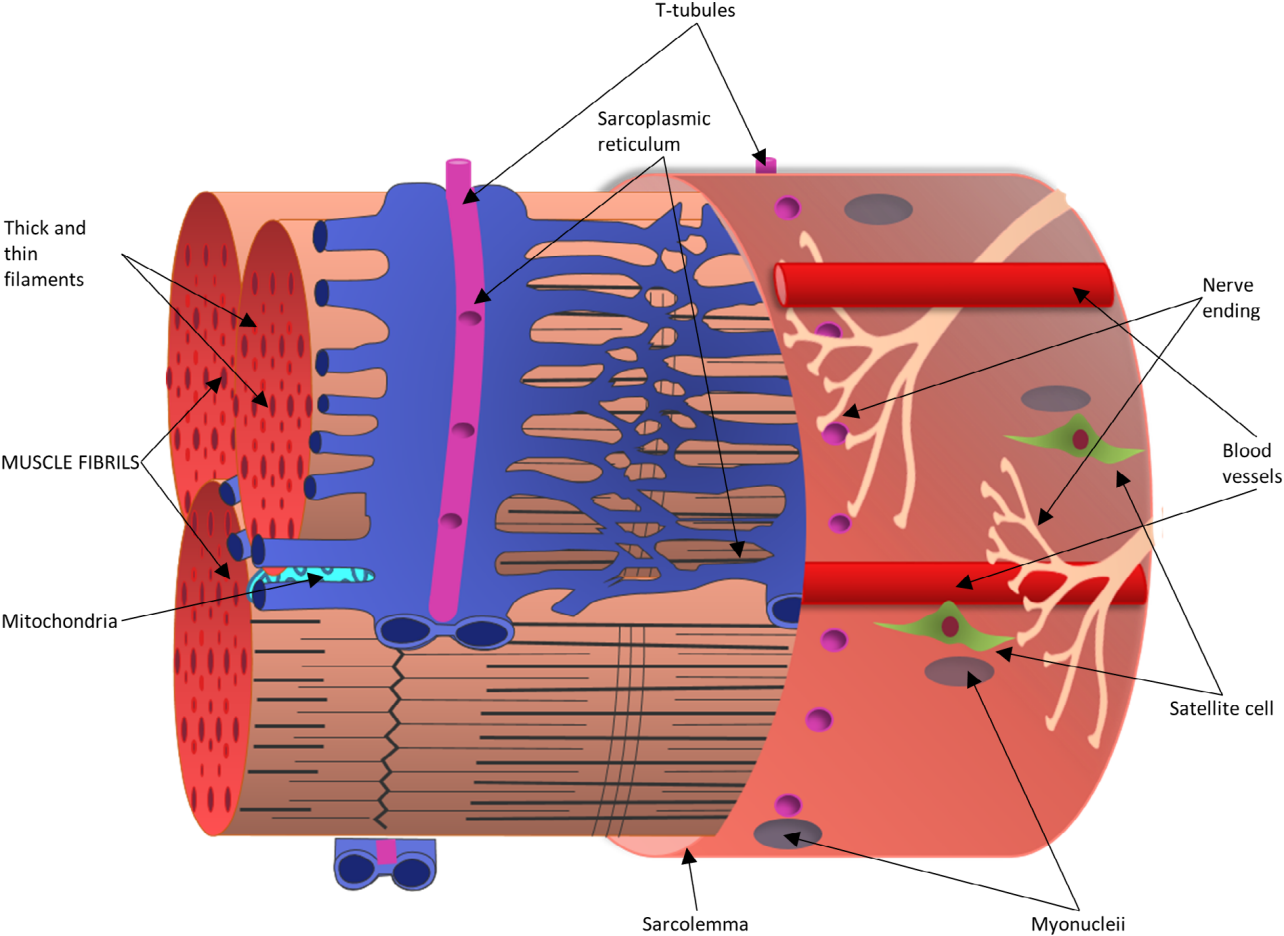
Schematic representation of skeletal muscle fiber—a single mature muscle fiber is shown here as a bundle of myofibrils, encased by the sarcolemma. The sarcoplasmic reticulum enmeshes fibrils with transverse (T) tubules intersecting them. Bundles of myofibers form fascicles, which further group together to form the muscle tissue. Satellite cells reside along the host muscle fiber, directly above the sarcolemma under the basal lamina of muscle and in proximity of myonuclei. Innervating nerve fibers and local capillaries extend along the length of the muscle fiber. Each layer is successively encased by the extracellular matrix, not shown here

Striated muscle is composed of two major muscle types—skeletal and cardiac. While the cardiac (heart) muscle functionally represents a set of self‐stimulating, non‐fatiguing muscle cells with an intermediate energy requirement, skeletal muscle represents a set of innervated, voluntary muscle cells that exhibit fatigue with high energy requirements (e.g., muscles of the thigh or forearm). A cursory glance at the cellular structure and molecular cross‐talk allows us to appreciate the complexity in composition, structure and function of striated muscle, designed to accomplish the task of generating contraction, force and movement. Briefly, skeletal muscle is a highly organized tissue containing several bundles of muscle fiber (myofibers). Each myofiber (containing several myofibrils), represents a muscle cell with its basic cellular unit called the sarcomere. Bundles of myofibers form the fascicles, and bundles of fascicles form the muscle tissue, with each layer successively encapsulated by the extracellular matrix (ECM; Lieber, 2009) and supported by the cytoskeletal networks. Skeletal muscle is highly vascularized and innervated, and embedded with components of the metabolic and regulatory machinery, supporting efficient energy production and cellular homeostasis (Figure 1). Precisely coordinated activity between each of these components is essential for shaping the state of muscular health and associated motor activity. Any perturbations (e.g., genetic or environmental) to this coordination, result in loss of muscle health and function, typically characterized by muscle fiber loss, reduced motor output and in some cases death.

**Figure 2:**
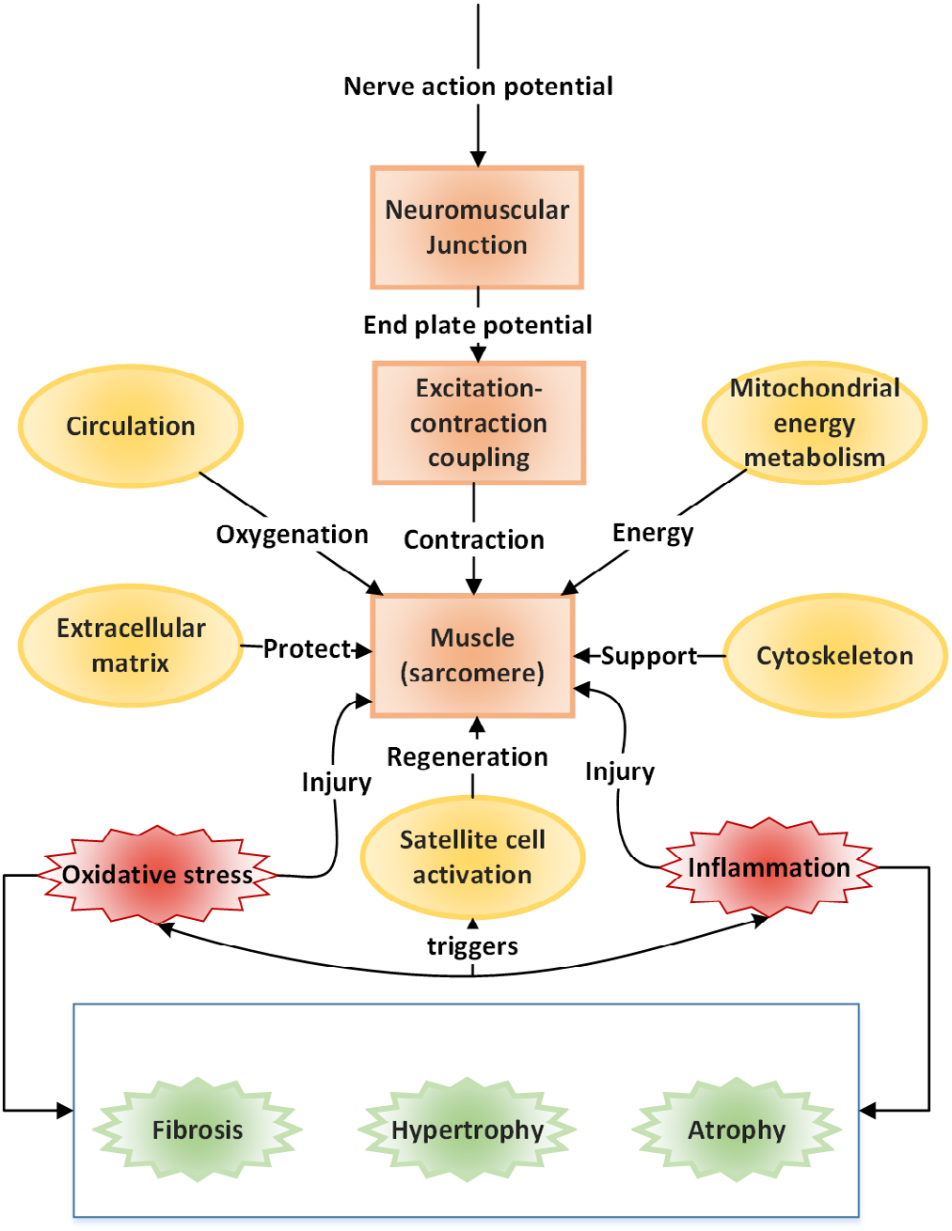
Components of muscle structure and function—a schematic representation of the various functional components necessary for or arising as a consequence of muscle function, in health and disease. The structure and function of each of these units are discussed in this current review. The arrows identify a one‐word description for each of the units and their role in governing normal muscle function

Over the decades, reviews in skeletal muscle research have focused extensively on specific aspects of muscle structure, or function. Our current review focuses on providing a more holistic picture of the various interacting components within skeletal muscle. In this review, we emphasize the idea of viewing the muscle as a biomechanical device requiring the coordination between several factors (or components) both intrinsic (e.g., genetic) and extrinsic (e.g., environmental stressors, circulatory factors, etc.) essential for normal muscle function. Within each of these components, we highlight the necessary molecular cross‐talk critical for defining its state. We also highlight instances of aberrant molecular mechanisms leading to disease, thus, bridging muscle research at genomic, molecular and mechanistic level, in health and disease (Figure 2).

This review begins with a focus on muscle tissue “development and regeneration”, outlining the embryological development of muscle, and the role for specific muscle regulatory factors in growth and development (Section 2). We also review satellite cell quiescence and activation that govern muscle regeneration and repair (Section 3). The “structural and functional” aspects of muscle, starting with the three most basic units that drive skeletal muscle contraction, namely (a) Neuromuscular junction (NMJ) which serves as a junction between nerve and muscle; (b) Machinery involved in excitation–contraction coupling (ECC), which is the process of transduction of electric impulses from nerve to muscle, required to initiate mechanical contraction; and (c) Sarcomere, the contractile apparatus required for force generation are discussed in Sections 4–6. Different muscle fiber types and the effect of exercise on fiber‐type remodeling are also presented. We next discuss the ECM which encapsulates the muscle, protecting it (Section 7), and the cytoskeleton, which is necessary for mechanical support, and capable of sustaining muscle's rapid contraction and relaxation cycles (Section 8). We discuss the pathophysiological changes arising in muscle as a response to triggers (such as inflammation, oxidative stress, exercise), specifically, the impact on structural and functional integrity of the muscle, such as fibrosis, hypertrophy and atrophy in Sections 7 and 9. Stress signaling (e.g., due to disease or injury) initiates a host of protective responses including inflammation and oxidative stress and are discussed in Section 10. Carbohydrate metabolism serves as the major energy source required for muscle function. We discuss the basic bioenergetics pathways associated with energy metabolism (glucose and fat) in Section 11, along with a brief introduction to the effect of exercise on metabolism. The dynamics of interaction between molecular actors of immunity and metabolism (immunometabolism) has been recently identified as vital to maintaining the health of skeletal muscle and is also discussed. The vasculature necessary for oxygenation required to sustain muscle is reviewed in Section 12, with a special emphasis on vascular endothelial growth factors (VEGFs). Through the sections, we highlight and emphasize molecular perturbations and clinical manifestations of relevant diseases affecting muscle (italicized in text). Finally, in Section 13, we summarize and highlight common molecular mechanisms underlying a spectrum of muscle disorders, identified in our work previously, and using a network theoretic approach.

Research in the past decade has increasingly acknowledged the contribution of noncoding components (e.g., long noncoding RNAs [lncRNAs], small open reading frames [smORFs]) to muscle development and function (Anderson et al., 2015; Andrews & Rothnagel, 2014; Fatica & Bozzoni, 2014; Gonçalves & Armand, 2017; Lim et al., 2018; Nelson et al., 2016; Nie, Deng, Liu, & Wang, 2015). However, it is beyond the scope of our current review and discussed only cursorily. The complexity in structure and function for each of the 13 units discussed here are immense, with several years of dedicated study by researchers. In this current review, we present a basic list of cellular components and molecular mechanisms for each unit, introducing the reader to the breadth of muscle research. In many instances, we use the more widely used names or symbols for several molecular markers within this review for improved readability. We provide their official gene symbol in Supplementary Table 1 for accuracy. The interested reader is directed to outstanding papers, of research and reviews, for in‐depth discussions of relevant mechanisms and concepts, within the individual topics discussed here.

## 2 MUSCLE EMBRYOLOGICAL DEVELOPMENT AND THE ROLE FOR MUSCLE REGULATORY FACTORS

The positions and identities of cells that will form the three germ layers (ectoderm, mesoderm, and endoderm) are determined early in gestation (S. J. Arnold & Robertson, 2009). The mesoderm is anatomically separated into paraxial, intermediate, and lateral mesoderm, based on the position from the midline/neural tube. Lineage tracing and fate‐mapping experiments have identified that embryonically, body skeletal muscle is derived from mesodermal precursor cells originating from the myotome, a somite‐derived lineage (Tajbakhsh & Cossu, 1997). Somites are bilaterally paired epithelial clusters that are formed by epithelialization of the paraxial mesoderm concomitant with segmentation. The processes of somite formation, segmentation and myogenesis are closely regulated by expression of genes involved directly or indirectly with WNT (von Maltzahn, Chang, Bentzinger, & Rudnicki, 2012), FGF (Pownall & Isaacs, 2010) and the inhibitory NOTCH (Buas & Kadesch, 2010) signaling pathways, in addition to the four myogenic regulatory factors (MRFs, MYOG1, MYOD, MRF4, and MYF5) (Bentzinger, Wang, & Rudnicki, 2012; Pownall, Gustafsson, & Emerson, 2002).

**Figure 3:**
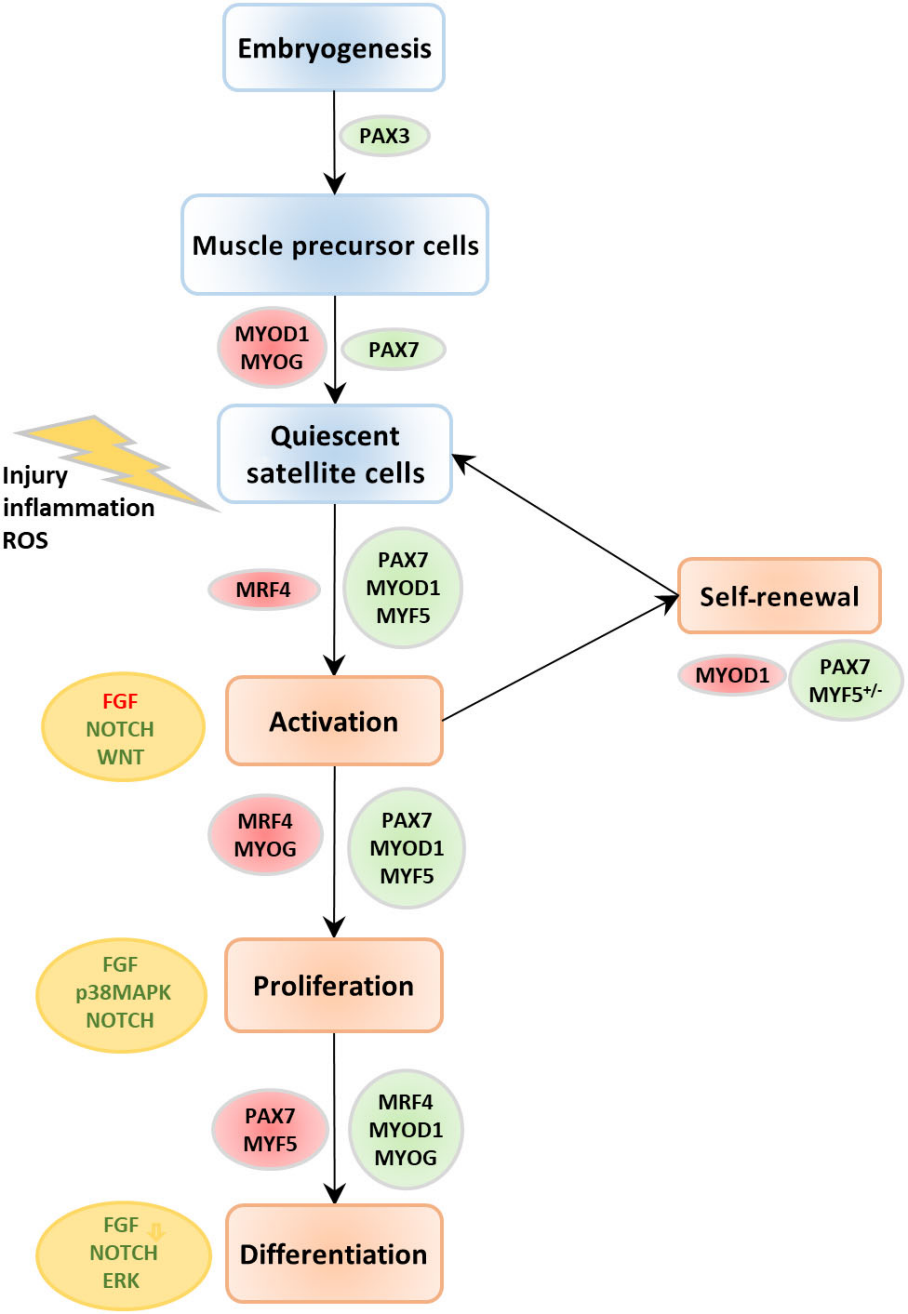
Expression of markers and pathways involved in stages of quiescence, activation and differentiation of satellite cells. During embryonic development, a portion of the muscle precursor cell population are incorporated into postnatal muscle as quiescent satellite cells which can transform again into muscle precursor cells (myogenic progenitor cells), upon activation. The major molecular markers and pathways that are necessary for transition of satellite cells from quiescent to a differentiated state are identified here. The markers/pathways that are upregulated are shown in green, downregulated in red

PAX3, a transcription factor, controls migration of muscle precursor cells by regulating LBX1 and cMET (Birchmeier & Brohmann, 2000). SIX1 and SIX4, two transcription factors are considered to be at the apex of the regulatory cascade that establishes the myogenic lineage of the precursor cells (Bentzinger et al., 2012; Grifone et al., 2005). Myoblasts activate MYF5 and MYOD1, two MRFs that control specification of head, epaxial, hypaxial and limb body muscle progenitors of the vertebrate embryo and mark a commitment to the muscle lineage. MYOD1 expression persists beyond differentiation, while MYF5 ceases during differentiation. Activation of a second wave of MRFs (MYOG and MRF4) induces terminal differentiation of myoblasts into myocytes that additionally express muscle‐specific genes such as the contractile proteins of the muscle (myosin, actin, etc.) and muscle creatine kinase. The mononucleated myocytes eventually fuse to form multinucleated, mature, contracting muscle fibers (Figure 3). However, an understanding of specific molecular mechanisms controlling cell fusion of myocytes to mature myofibers is yet to be achieved. Recently, a minimal “two component program” for the induction of mammalian myocyte fusion comprising of Minion, an essential microprotein and Myomaker, a transmembrane protein (Gamage et al., 2017; Millay et al., 2013; Millay, Sutherland, Bassel‐Duby, & Olson, 2014), have been identified as sufficient for fusion (Q. Zhang, Vashisht, O'Rourke, et al., 2017). During the late phase of embryonic myogenesis, a distinct population of somite‐derived precursor cells remain in a quiescent undifferentiated state closely associated with myofibers (Lepper & Fan, 2010) and are called (adult) satellite cells (SCs). Many shared components including transcription factors and signaling molecules exist between embryonic myogenesis and muscle regeneration by SC activation in mature skeletal muscle (Tajbakhsh, 2009), as will be seen in the following section detailing SC quiescence, activation and muscle regeneration.

## 3 SATELLITE CELLS AND MUSCLE REGENERATION

Regeneration is one of the hallmarks of mature skeletal muscle tissue. Its ability to regenerate is governed significantly by the interaction between SCs (Scharner & Zammit, 2011) (SCs, unipotent muscle precursor cells) and its microenvironment (niche) (Lander, Kimble, Clevers, et al., 2012). Muscle regeneration is a highly orchestrated process, which involves activation and migration of SCs to the site of injury and their proliferation and differentiation into muscle fibers.

SCs represent a population of adult stem cells, mostly derived from PAX3^+^/PAX7^+^ embryonic progenitor cells (Buckingham, 2007), and incorporated into growing fibers during postnatal muscle development. Anatomically, SCs appear wedged between basal lamina (BL), and the sarcolemma, sequestered in a particular microenvironment called the “niche,” within the adult skeletal muscle (Yin, Price, & Rudnicki, 2013). These cells are in a “quiescent”/hibernating state. The BL serves as a scaffold for SCs and functions to limit and orient their migration during injury (Sanes, 2003). BLs present a large number of binding sites for integrins‐α7/integrin‐β1, which anchor the actin cytoskeleton of SCs to the BL (Blanco‐Bose, Yao, Kramer, & Blau, 2001). This tethering also serves to relay extracellular mechanical cues (from myofibers) into intracellular chemical signals (within the SCs) (Boppart, Burkin, & Kaufman, 2006). The niche embedding the SCs is composed of both acellular and cellular components, including growth factors (GFs), ECM proteins, fibroadipogenic progenitors (FAPs), chemokines, and matrix metalloproteinases (MMPs). Beyond the immediate niche, local interstitial cells, motor neurons, vasculature and secreted factors (e.g., see Section 12.1), all have an ability to influence SC activity (Dumont, Wang, & Rudnicki, 2015; Yin et al., 2013).

The SC population is heterogeneous, differing in lineage potential, expression patterns, and myogenic differentiation potential (Kuang, Kuroda, Le Grand, & Rudnicki, 2007). The SC population is maintained uniformly, which however reduces in population density and efficacy with age (Almada & Wagers, 2016). Functional differences in regenerative potential exist between satellite stem cells (never expressed MYF5) and committed myogenic progenitor cells (that have expressed MYF5 at some point in development). Following transplantation, SCs preferentially repopulate the SC niche and contribute to long‐term muscle regeneration in a PAX7‐dependent manner (Günther et al., 2013).

### 3.1 Satellite cell quiescence

**Figure 4:**
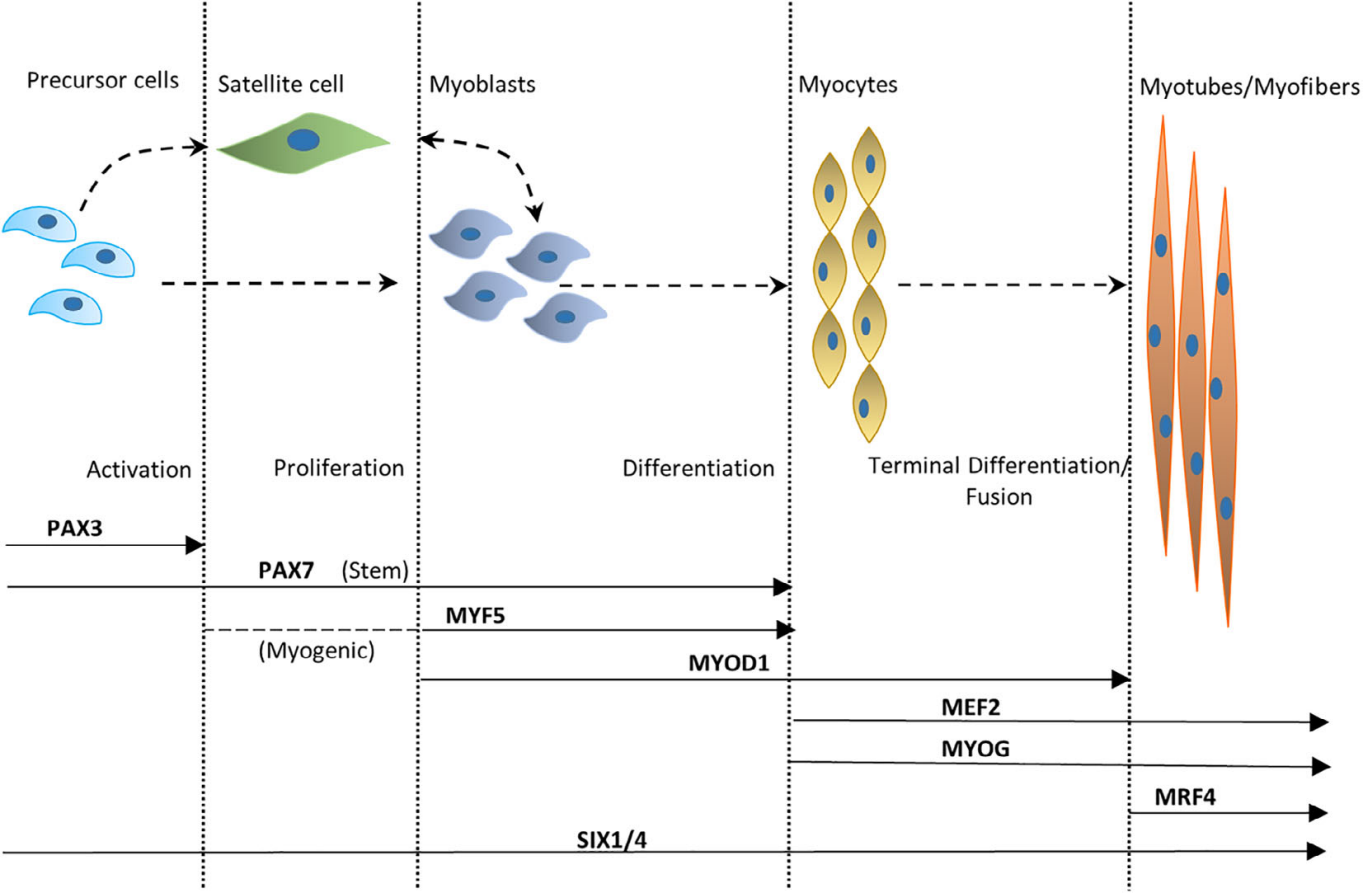
Hierarchy of transcription factors regulating myogenic lineage. This figure represents the major transcription factors involved in muscle development and shows their temporal sequence of activation across various stages of myogenesis. Satellite stem cells expressing PAX7 derive from the PAX3/PAX7 expressing progenitors, whereas satellite myogenic cells additionally exhibit an activation of MYF5. Following activation and entrance into the cell cycle, stem cells express MYF5 and MYOD1. Activation of MYOG and MEF2C, with downregulation of MYF5 and later MYOD1 mark the start of terminal differentiation. Activation of MRF4 happens several days after the induction of differentiation, following a reduction in MYOG

Quiescence defines a state of dormancy in adult stem cells, with quiescent SCs (QSCs) exhibiting an ability to rapidly activate, proliferate and differentiate into myofibers upon injury. The QSCs are characterized by the expression of definitive molecular markers, particularly PAX7, and a marked absence of two MRFs, MYOD1 and MYOG (Figure 4). Activation of NOTCH (Bjornson et al., 2012) and WNT signaling is essential for maintaining quiescence in SCs by inhibiting MYOD1 expression and inducing PAX7 (Olguin & Olwin, 2004). Recent work has identified an alternative pathway for NOTCH activation involving FOXO3 in QSCs (Gopinath, Webb, Brunet, & Rando, 2014). Several other molecular markers regulating quiescence have been identified including cell cycle inhibitors such as p21, p27 (Fukada et al., 2007), and DACH1 (which inhibits cell cycle progression and regulates activity of pro‐myogenic SIX1 and SIX4) (Pallafacchina et al., 2010). Skeletal muscle‐specific TGFβ family member, myostatin, suppresses SC activation via induction of p21 (McCroskery, Thomas, Maxwell, Sharma, & Kambadur, 2003; Thomas et al., 2000). Retinoblastoma proteins (Carnac et al., 2000; Weinberg, 1995), and activated ID proteins (Benezra, Davis, Lockshon, Turner, & Weintraub, 1990) (particularly ID3; Kumar, Shadrach, Wagers, & Lassar, 2009) have also been identified as essential markers of QSCs. Activated CALCR, a calcitonin receptor, serves as both a spatial and temporal regulator of QSCs (Fukada et al., 2007; Yamaguchi et al., 2015). SPRY1, a tyrosine inhibitor kinase, is necessary for maintenance and re‐entry of PAX7^+^ SCs into quiescence (Shea, Xiang, LaPorta, et al., 2010). Additionally, integrin‐β1 and CXCR4, integrin‐α7 and CD34 are all definitive cell surface markers for QSCs in skeletal muscle, in vivo (Maesner, Almada, & Wagers, 2016). A detailed review of additional molecular markers, metabolic states, and mobility of QSCs is presented in Rocheteau, Vinet, and Chretien (2015).

### 3.2 Satellite cell activation, differentiation, and proliferation

In response to muscle injury, several environmental cues (niche) and chemical signals trigger activation of SCs, signaling the proliferation and differentiation of SCs to mature fibers, replacing damaged ones. Activated SCs (ASCs) are characterized by PAX7 and MRF expression (MYOD1, MYOG, and MYF5). The relative expression of MYOD1, MYOG, and MYF5 in PAX7^+^ cells and their temporal sequence regulates and maintains ASC proliferation (reviewed in detail in Yin et al., 2013; Figure 4). Terminal differentiation begins with downregulation of MYF5 and later MYOD1, and a concerted expression of MYOG, MEF2C, and MRF4 much later. Downstream targets of MYOD1 and MYOG (including MEF2s), further activate fiber type specific contractile and cytoskeletal genes (Cooper et al., 1999; Yin et al., 2013). Several mechanisms are suggested to play a role in the activation of MRFs and its downstream targets (Francetic & Li, 2011). For instance, MYF5 is induced via the methyltransferase CARM1's action on PAX7 and recruitment of histone acetyltransferases to the enhancers of MYF5 (Kawabe, Wang, McKinnell, Bedford, & Rudnicki, 2012). PAX3 also regulates early MYF5 expression via direct regulation of DMRT2 (Sato, Rocancourt, Marques, Thorsteinsdóttir, & Buckingham, 2010). SIX family of proteins (SIX1, SIX4) regulate MYOG expression, particularly, SIX4 repress MYOG, while SIX1 activate MYOG expression, thereby regulating proliferation and differentiation fates of ASCs (Yajima et al., 2010).

The migration to, and proliferation of SCs at the site of injury is driven by chemoattractants (released from the ECM or from the inflammatory cells), mostly, GFs such as VEGFs (see Section 12.1), fibroblast GFs, insulin GFs, and hepatocyte GFs, damage‐associated molecular patterns (Hindi & Kumar, 2016; Lotze et al., 2007), and cytokines (TNFα and TGFβ) released by resident cells and infiltrating inflammatory cells (Allen & Boxhorn, 1989; Christov et al., 2007; Y.‐P. Li, 2003; Sheehan & Allen, 1999; Tidball & Villalta, 2010). The JAK‐STAT pathway, activated by various cytokines, has been suggested to play a crucial role in early myogenic differentiation (K. Wang, Wang, Xiao, Wang, & Wu, 2008) and SC proliferation and differentiation (Doles & Olwin, 2014). More recent studies also demonstrate the requirement of Gαi2, the α‐subunit of the heterotrimeric G‐protein complex, for SC differentiation in a protein kinase C and histone deacetylase (HDAC)‐dependent manner (Minetti et al., 2014).

During regeneration, a portion of the ASC population has the capacity to return to quiescence to maintain the SC pool, essential for maintaining muscle integrity. STAT3 has been shown to regulate the self‐renewal potential of SCs (H. Zhu et al., 2016), in injured muscle, during muscle regeneration. STAT3 is also associated with SC proliferation in an IL‐6‐dependent manner upon injury (Toth et al., 2011). The local production of IL‐6 by skeletal muscle cells and stromal cells upon injury/exercise promotes SC activation, though the precise signaling mechanism of IL‐6‐dependent SC activation and proliferation, under various physiological states (e.g., injury, aging) is under much scrutiny (Belizário, Fontes‐Oliveira, Borges, Kashiabara, & Vannier, 2016; Brack & Muñoz‐Cánoves, 2015). p38MAPK serves as a powerful regulator of myogenesis via regulation of MRF activation (Lluís, Perdiguero, Nebreda, & Muñoz‐Cánoves, 2006) and stem cell renewal and quiescence (Segalés, Perdiguero, & Muñoz‐Cánoves, 2016). Fibroblast GF signaling serves as a potent activator of both STATs and p38MAPK in SCs (Pawlikowski, Orion Vogler, Gadek, & Olwin, 2017).

Mechanistic insights into the metabolic constraints for maintaining quiescence and transitioning to a proliferating/differentiating state are still in its infancy. Current research points to a switch from oxidative phosphorylation as energy source in quiescence to glycolysis in proliferating SCs (Koopman, Ly, & Ryall, 2014). The presence of an autophagic flux via the activation of SIRT1, a NAD^+^/NADH (nutrient) sensor, in QSCs is suggested as being required to meet the bioenergetics demands of the SC upon activation (Pardo & Boriek, 2011; Tang & Rando, 2014).

An understanding of posttranslational modifications and epigenetic control on SC quiescence, proliferation and differentiation states is gaining momentum and has been reviewed in detail in Segalés et al. (2016). They form an important mechanism for regulating the activation and activity of MRFs and subsequently of myogenesis (Giordani & Puri, 2013; Puri & Sartorelli, 2000; Saccone & Lorenzo, 2010).

Research in dystrophies have shown impacted activity of SCs which additionally undergo premature senescence (akin to sarcopenia) and a significant reduction in their population sizes, contribute to a reduction in muscle regenerative capacity (Heslop, Morgan, & Partridge, 2000; Jiang et al., 2014; Kudryashova, Kramerova, & Spencer, 2012; Yablonka‐Reuveni & Anderson, 2006). Efforts are underway to rejuvenate stem cells to mitigate the effects of stem‐cell aging on muscle regeneration (Bengal, Perdiguero, Serrano, & Muñoz‐Cánoves, 2017) with a possibility of offering therapeutic relief in chronic diseases such as the dystrophies.

In the following sections, we review the basic molecular structure of muscle tissue and the components that enable muscle function.

## 4 NEUROMUSCULAR JUNCTION

**Figure 5:**
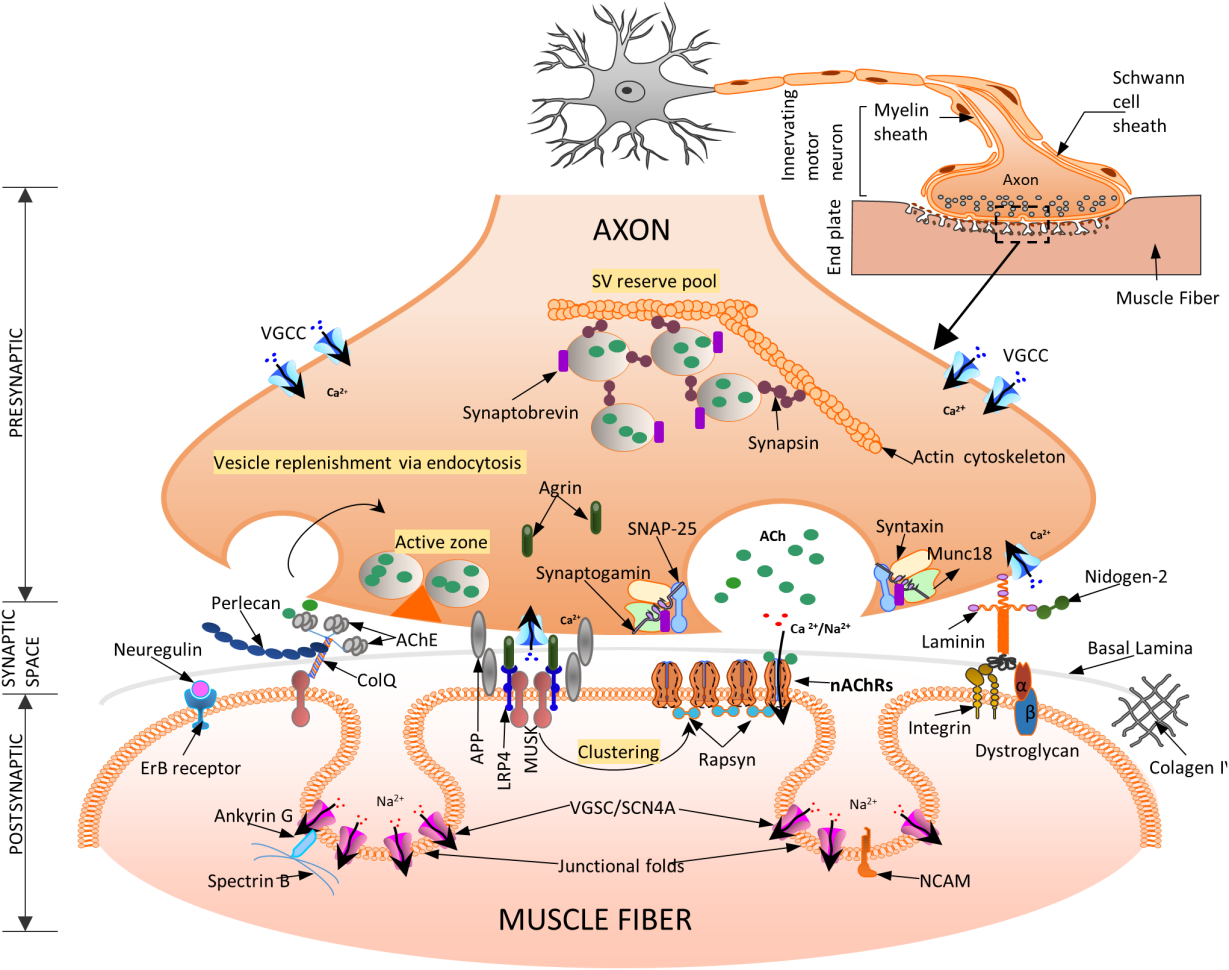
A schematic representation of a neuromuscular junction (NMJ) and its main molecular actors—three specific regions define the NMJ: (a) the presynaptic motor nerve terminal where vesicles fuse with the terminal membrane to release acetylcholine (ACh) into the synaptic cleft. Calcium influx through the voltage‐gated Ca channels (VGCC) trigger vesicle fusion and release from the active zones (described in detail in Section 5.1); (b) the synaptic space contains the basal lamina (BL, extra cellular matrix layer), and shows the presence of AChE‐ColQ (essential for the inactivation of ACh). ColQ binds MuSK and Perlecan necessary for stabilization of BL. MuSK enables AChR clustering via rapsyn (detailed in Section 5.2); (c) postsynaptic organization of the skeletal muscle membrane include several folds with receptors for the diffusing ACh (AChRs) at the crest and voltage‐gated sodium channels (VGSC) in the troughs of the folds necessary for efficient neuromuscular transmission. The agrin‐Lrp4‐MuSK complex, present on the trough of the postsynaptic membrane is essential for the formation of the NMJ (described in detail in Section 5.3). The entire structure is finally attached to the actin cytoskeleton (not shown here for simplicity)

NMJ is the chemical synapse responsible for transmission of electric impulses from the innervating motor neuron to the innervated muscle fibers. The complexity and distribution of NMJs on the surface of muscle fibers differ greatly within and between muscle fibers in health and disease (Hall & Sanes, 1993; Hughes, Kusner, & Kaminski, 2006; Sanes & Lichtman, 1999). The NMJ comprises of three major regions: (a) the presynaptic region, comprising of the Schwann cell which envelops the nerve terminal containing the neurotransmitter; (b) the synaptic space lined by the basement membrane; and (c) the postsynaptic region containing the junctional sarcoplasm, and the postsynaptic membrane which contains receptors for the neurotransmitter (Figure 5).

### 4.1 Presynaptic region

Schwann cell envelops much of the nerve terminal at the NMJ, except the part that faces the postsynaptic membrane. The nerve terminal contains an abundance of synaptic vesicles (SVs), which function to store, release and uptake the neurotransmitter, acetylcholine (ACh) (Denker & Rizzoli, 2010; Rizzoli & Betz, 2005). SVs fuse to the presynaptic membrane at “active zones” initiating neuromuscular transmission (Nishimune, 2012). Active zones are visually dense zones, containing specialized proteins (such as Piccolo, Bassoon, and RIM1, interconnected by fibrils and embedded in a matrix), at the presynaptic membrane. Active zones are associated with vesicle docking and fusion, exocytosis, and vesicle recovery (Ackermann, Waites, & Garner, 2015). SVs are known to dock at active zones in highly definite patterns (Harlow, Ress, Stoschek, Marshall, & McMahan, 2001; Szule et al., 2012). Synapsin is suggested to anchor vesicles in reserve pools to the actin cytoskeleton, which are transported to active zones by myosin motors on actin tracks upon synaptic ingress of Ca^2+^ via presynaptic P/Q type voltage‐gated calcium channels (VGCCs P/Q type; Cai & Sheng, 2009; Südhof, 2004). Rapid exocytosis from active zones is closely orchestrated by Ca^2+^ and subsequently by the VGCC. Lambert–Eaton myasthenic syndrome, a rare autoimmune disease of the presynaptic membrane, manifests when IgG antibodies cross‐link VGCC, leading to a disruption of normal architecture and affecting active zone complexes (Fukunaga, Engel, Osame, & Lambert, 1982). The coupling mechanisms and modes of exocytosis of SVs are varied and are suggested to depend largely on muscle type and stimulus (Alabi & Tsien, 2013; L.‐G. Wu, Hamid, Shin, & Chiang, 2014).

The exocytotic machinery comprises mainly of the soluble NSF‐attachment protein receptor (SNARE) and SEC1/MUNC18‐like (SM) proteins, which bring vesicles in close proximity of the presynaptic membrane (reviewed in Südhof & Rizo, 2011). Formation of a SNARE complex (the SNARE pin) occurs in three steps: (a) Presynaptic membrane‐associated SNAP25 binds syntaxin‐1 forming a complex (t‐SNARE) at the presynaptic membrane. SM proteins (specifically MUNC18) binds to assembling SNARE complex via syntaxin‐1, and has been shown to be essential for SV fusion in vivo (Shen, Tareste, Paumet, Rothman, & Melia, 2007); (b) Synaptogamins serve as a sensor for presynaptic Ca^2+^ and bind with the t‐SNARE, bringing the vesicle in close proximity to the presynaptic membrane (C. Wang, Bai, Chang, Chapman, & Jackson, 2006); (c) t‐SNARE engages vesicle‐associated VAMP/synaptobrevin to complete the formation of the SNARE complex. Complexins (CPLX1), that bind syntaxin‐1 with synaptobrevins, play a role in both repressing and activating SNARE‐dependent vesicle fusion, in conjunction with Ca^2+^ activated synaptogamins (Maximov, Tang, Yang, Pang, & Südhof, 2009). Botulinum neurotoxins, a class of bacterial poisons, target various proteins of this exocytotic machinery leading to a failure in neurotransmission and eventual paralysis (Pirazzini, Rossetto, Eleopra, & Montecucco, 2017). Following exocytosis, endocytosis rapidly recycles vesicles, vesicular membrane proteins and sustains further exocytosis. NSF, neurexin, and α‐SNAP are known to be involved with the disassembly of SNAREs following exocytosis and play a crucial role in maintaining fusion dynamics and vesicle recovery within the synapse (C. Zhao, Slevin, & Whiteheart, 2007).

### 4.2 The synaptic space and the synaptic basal lamina

Space between the pre‐ and postsynaptic membranes through which ACh diffuses, is divided into the primary cleft (bounded by the presynaptic membrane and the basement membrane) and the secondary clefts (space between the junctional folds of the postsynaptic membrane). Center of the synaptic cleft is occupied by the synaptic BL (basement membrane, BL). In addition to a mechanical role, synaptic BL plays an important role in NMJ innervation, development and regeneration, specifying architecture and physiological roles of pre‐ and postsynaptic membranes in both normal and disease pathology (Sanes, 2003). Components of the synaptic BL include laminins (4, 9, and 11) (Rogers & Nishimune, 2017), collagens IV, and nidogen‐2 (Fox, Ho, Smyth, & Sanes, 2008). A portion of the diffusing ACh is hydrolyzed by AChE, promoting cessation of signal transmission (Soreq & Seidman, 2001). AChE is anchored to the BL via COLQ and perlecan (Anglister & McMahan, 1985; Kimbell, Ohno, Engel, & Rotundo, 2004). Expression of perlecan is crucial for localizing AChE to the synaptic BL (Arikawa‐Hirasawa, Rossi, Rotundo, & Yamada, 2002), while COLQ is suggested to control postsynaptic differentiation (Sigoillot, Bourgeois, Lambergeon, Strochlic, & Legay, 2010). Agrin, a NMJ heparin sulfate (HS) proteoglycan (PG), critical for organization of the ACh receptors and NMJ, is found in the BL along with neuregulin which acts downstream of agrin (Mc Mahan, 1990). ECM in the synaptic space also plays a role in, reinnervation (Glicksman & Sanes, 1983; Sanes, Marshall, & McMahan, 1978) and synaptic adhesion (Yamagata, Sanes, & Weiner, 2003).

### 4.3 Postsynaptic region

Postsynaptic region consists of junctional folds, which amplify the postsynaptic membrane area and consequently the volume of synaptic space, and the junctional sarcoplasm (Figure 5). Junctional sarcoplasm fills the synaptic space and contains several cellular structures such as mitochondria, Golgi apparatus, and intermediate filaments (IFs), required to meet the metabolic and structural needs of the postsynaptic region.

**Figure 6:**
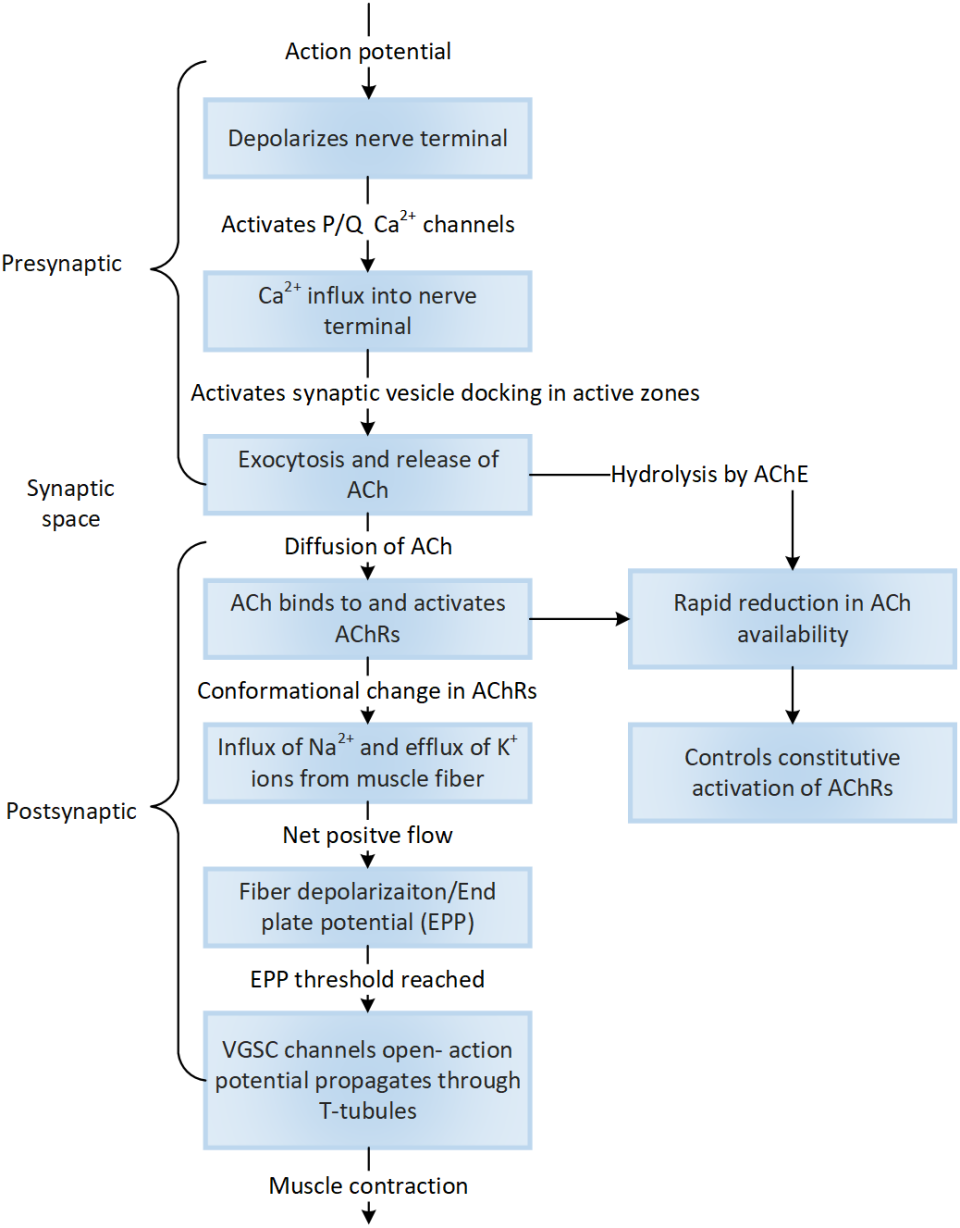
The sequence of cellular events associated with synaptic signaling that activates the cascade of downstream events towards muscle contraction are identified here

The terminal expansions (crests) of the junctional folds are packed with nicotinic acetylcholine receptors (nAChRs) which are pentameric ion channels with subunits α, β, γ, δ, and ε (Kramer, 2016) which are linked via rapsyn (Zuber & Unwin, 2013). ACh that reaches the postsynaptic membrane activates nAChRs, creating a local depolarization potential. Under normal physiological conditions, nAChR are impermeable to Cl^−^ ions but allow Na^2+^ and K^+^ ions and to a lesser extent Ca^2+^ and Mg^2+^ ions. The magnitude and direction of current through the nAChRs depends however on the membrane potential. This in turn activates the voltage‐gated sodium channels (VGSCs) concentrated in the troughs of junctional folds (Awad et al., 2001), along with neural cell adhesion molecule (Rafuse, Polo‐Parada, & Landmesser, 2000), creating an action potential which is transmitted through the fiber via the T‐tubules. Ankyrin‐G and β‐spectrin are essential for maintaining VGSC densities in the postsynaptic folds, necessary for impulse propagation (Flucher & Daniels, 1989; Tee & Peppelenbosch, 2010; Wood & Slater, 1998). MUSK a master regulator of NMJ development is suggested to induce AChR clustering via agrin and its co‐receptor, LRP4 (Zong et al., 2012). Detailed reviews of agrin associated signaling via muscle‐specific and cytoskeletal proteins (e.g., MUSK, LRP4), necessary for AChR clustering and formation of postsynaptic structures are presented in Bezakova and Ruegg (2003) and H. Wu, Xiong, and Mei (2010) (Figure 6).

Additionally, research has indicated important organizational roles for amyloid precursor proteins (APP, APLP1, APLP2) specifically trans‐adhesion of the post‐ and presynaptic membranes via their interaction with LRP4 and agrin at the NMJ (H. Y. Choi et al., 2013; Klevanski et al., 2014). Neuregulin (a neural trophic factor similar to agrin) and its receptors ERBB2/3/4 aggregate on the postsynaptic membrane. Neuregulin/ERBB signaling is suggested to function in stabilizing agrin‐induced AChR clusters, via phosphorylation of αDystrobrevin‐1, subsequently maintaining organization of the adult NMJ (Schmidt et al., 2011).

Myasthenia gravis (MG, acquired, neonatal and congenital) represent the largest group of progressive disorders caused due to impaired signal transmission across the motor end plates due to perturbations to a single (SCN4A mutations, Tsujino et al., 2003) or multiple proteins associated with postsynaptic membrane (nAChR degradation or its associated proteins; rapsyn, agrin, etc.; Engel, 2014). MG is characterized first by loss of control and weakness in eye muscles, followed by throat and neck and subsequently limb muscles. Majority of the acquired and neonatal MG cases are associated with IgG antibody cross‐linking of the postsynaptic nAChRs, resulting in the reduction of the number of effective receptors. Autoantibody binding, results in increasing degradation or nAChRs and subsequent damage to the postsynaptic membrane and its dynamics with synaptic folds, leading to impacted muscle contraction (Hirsch, 2007). Recent research has also shown mutations in COL13A1 (a transmembrane collagen shown to regulate synaptic integrity via its binding to COLQ) resulting in a novel subtype of congenital MG (Härönen et al., 2017; Logan, Cossins, Cruz, et al., 2015).

## 5 EXCITATION CONTRACTION COUPLING

**Figure 7:**
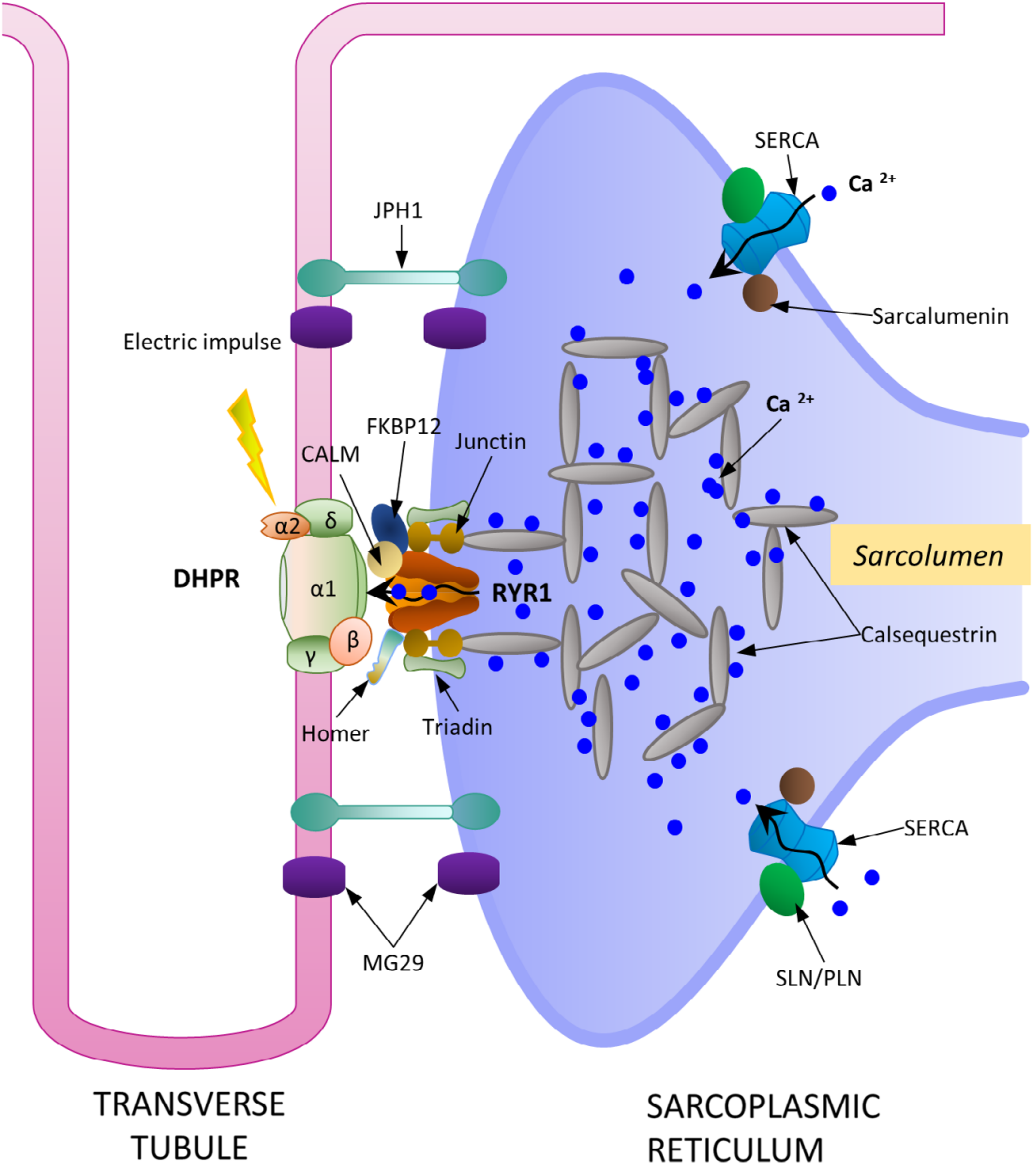
A schematic representation of the important molecular actors involved in excitation contraction coupling at the triad junction. DHPR, RYR1, SERCA pump, along with calsequestrin form the main proteins responsible for Ca^2+^ cycling and storage within the sarcoplasmic reticulum. Calsequestrin is a high capacity Ca^2+^ binding protein found in dense, highly concentrated filamentous matrices within the terminal cisternae of sarcoplasmic reticulum. JPH1 and MG29, are suggested to play significant roles in maintaining the structural integrity of this junction

Muscle contraction begins with the activation of fast sodium channels (postsynaptic voltage channels, SCN4A), generating an action potential that is transmitted to the muscle fiber, initiating contraction. This process, called ECC occurs at the junction between two membranous structures, namely, the transverse tubules (T‐tubules) and the sarcoplasmic reticulae, called the triad junction (Figure 1 for a birds‐eye view, Figure 7). The transmitted nerve action potential depolarizes the dihydropyridine receptor (DHPR) of the T‐tubules, a voltage‐gated Ca^2+^ channel (VGCC L‐Type), which in turn triggers the intracellular release of a large bolus of Ca^2+^ from the sarcoplasmic reticulum (SR) terminal cisternae via the ryanodine receptors (RYRs, calcium release channels). DHPR is suggested to act as a voltage sensor in skeletal muscle, and controls the opening of RYRs through direct molecular interactions (Franzini‐Armstrong, 2004). Dominant point mutations in DHPR (Ptáček, Tawil, Griggs, et al., 1994), and in SCN4A (Jurkat‐Rott et al., 2000), are associated with hypokalemic periodic paralysis, a disease characterized by muscle weakness/loss in fiber strength at low extracellular potassium levels.

In healthy cells, large amounts of Ca^2+^ are effectively sequestered in the vicinity of RYRs, within the SR lumen by calsequestrin (CASQ). CASQ, a very high affinity Ca^2+^ binding protein, sequesters large amounts of Ca^2+^ in densely concentrated filamentous matrices within the terminal cisternae of SR (Beard, Laver, & Dulhunty, 2004) and is suggested to regulate RYR dynamics (Beard et al., 2005) affecting muscle contractility. Two proteins, mitsugumin29 (MG29) and junctophilin function to maintain the structural and functional integrity of the triad junction. Junctophilin physically docks SR to the T‐tubule (Takeshima, Komazaki, Nishi, Iino, & Kangawa, 2000) maintaining the spatial proximity and MG29 is suggested to co‐localize within the junction (N. R. Brandt & Caswell, 1999; Takeshima, Shimuta, Komazaki, et al., 1998) and is necessary for efficient signal transduction of ECC between the SR and T‐tubules (Komazaki, Ito, Takeshima, & Nakamura, 2002; Nishi et al., 1999). Two other integral membrane proteins triadin (which aids in sequestering of CASQ) and junctin (a CASQ binding protein) are both suggested to form a quaternary complex with CASQ and RYR and are required for the normal regulation of Ca^2+^ release (Györke, Hester, Jones, & Györke, 2004; L. Zhang, Kelley, Schmeisser, Kobayashi, & Jones, 1997). RYR1 interacts with several other proteins integral to the SR such as FKBP1A, Homer, and calmodulin (CaM) leading to tight regulation of Ca^2+^ concentrations for efficient coupling and force generation. FKBP1A and Homer are essential for stabilization, and proper functioning of the Ca^2+^ release channels within muscle (Avila, Lee, Perez, Allen, & Dirksen, 2003; Pouliquin & Dulhunty, 2009). CaM, a soluble Ca^2+^ binding protein binds RYR and activates/inhibits its function depending on cytosolic Ca^2+^ concentration (Tripathy, Xu, Mann, & Meissner, 1995). Ca^2+^/CaM‐dependent protein kinases (CaMK), specifically CaMKII, associated with the terminal cisternae of SR are shown to phosphorylate a series of proteins within the SR and regulate their function directly affecting ECC (Chin, 2005). A newly discovered Z‐disk protein NRIP, is suggested to activate CaMKII throughCa^2+^‐dependent binding with CaM regulating mitochondrial function, slow myosin expression and muscle regeneration (Chen et al., 2015). Recent studies have identified S100A1 as a physiological modulator of RYR1, which structurally alters the RYR1/CaM complex suggesting complex dynamics between the three players at varying Ca^2+^ concentrations (Rebbeck et al., 2016).

Genetic defects in Ca^2+^ release channels (RYR1) are associated with two diseases classified broadly under congenital myopathies, namely, malignant hyperthermia (MH) and central core disease (CCD). CCD is a rare, inherited, non‐progressive myopathy characterized by loss in muscle tone and muscle weakness, accompanied often by MH (Jungbluth, 2007). Patients with MH exhibit adverse responses to inhalational anesthetics and muscle relaxants. Physiologically, in the presence of triggering agents such as anesthetics, mutated release channels (i.e., RYR1) flood the cell with spontaneous and enhanced rates of Ca^2+^, overpowering the Ca^2+^ pump action. Sustained muscle contractions lead to muscle rigidity, with increased rates of glycolytic metabolism, lactic acid production, CO_2_ and heat combined with an enhanced oxygen uptake. Loss of ion homeostasis and associated membrane damage lead to other life‐threatening systemic problems (hypoxemia, hyperkalemia, ventricular fibrillation, renal failure, and cyanosis) and in many cases, death (Loke & MacLennan, 1998).

The elevation of cytosolic Ca^2+^ brings a conformational change in troponin, beginning the cascade to muscle contraction. In contrast, muscle relaxation is brought about by removal of cytosolic Ca^2+^ and is associated with high chemical energy requirements. ATP‐dependent Ca^2+^ ATPase (SERCA pumps) densely packed on the non‐junctional face of the SR terminal cisternae function to return cytosolic Ca^2+^ released into the terminal cisternae (Periasamy & Kalyanasundaram, 2007). Three homologous ATP2A genes have been identified to encode three SERCA isoforms and their splice variants, with SERCA1a being ubiquitously expressed in mature skeletal muscle and SERCA1b in immature (fetal and neonatal) skeletal muscle. Additionally, SERCA1a binds sarcolipin (SLN) and phospholamban (PLN) (two homologs) that at low Ca^2+^ cytosolic concentrations significantly reduce SERCA's affinity to Ca^2+^, bringing about muscle relaxation (Espinoza‐Fonseca, Autry, & Thomas, 2015). Sarcalumenin (SRL), a luminal glycoprotein, plays a role in maintaining protein stability of SERCA pumps as well as buffering of Ca^2+^ in skeletal and cardiac muscles (Yoshida et al., 2005). Parvalbumin, a high Ca^2+^ affinity protein, present in the soluble sarcoplasm acts as a relaxing factor by binding free Ca^2+^ and is directly correlated with relaxation speeds of mammalian fast muscle (Rall, 1996).

Excitation contraction coupling results in the contraction of the sarcomeric machinery as outlined in the next section.

## 6 MUSCLE CONTRACTION AND FORCE GENERATION

### 6.1 The sarcomere

**Figure 8:**
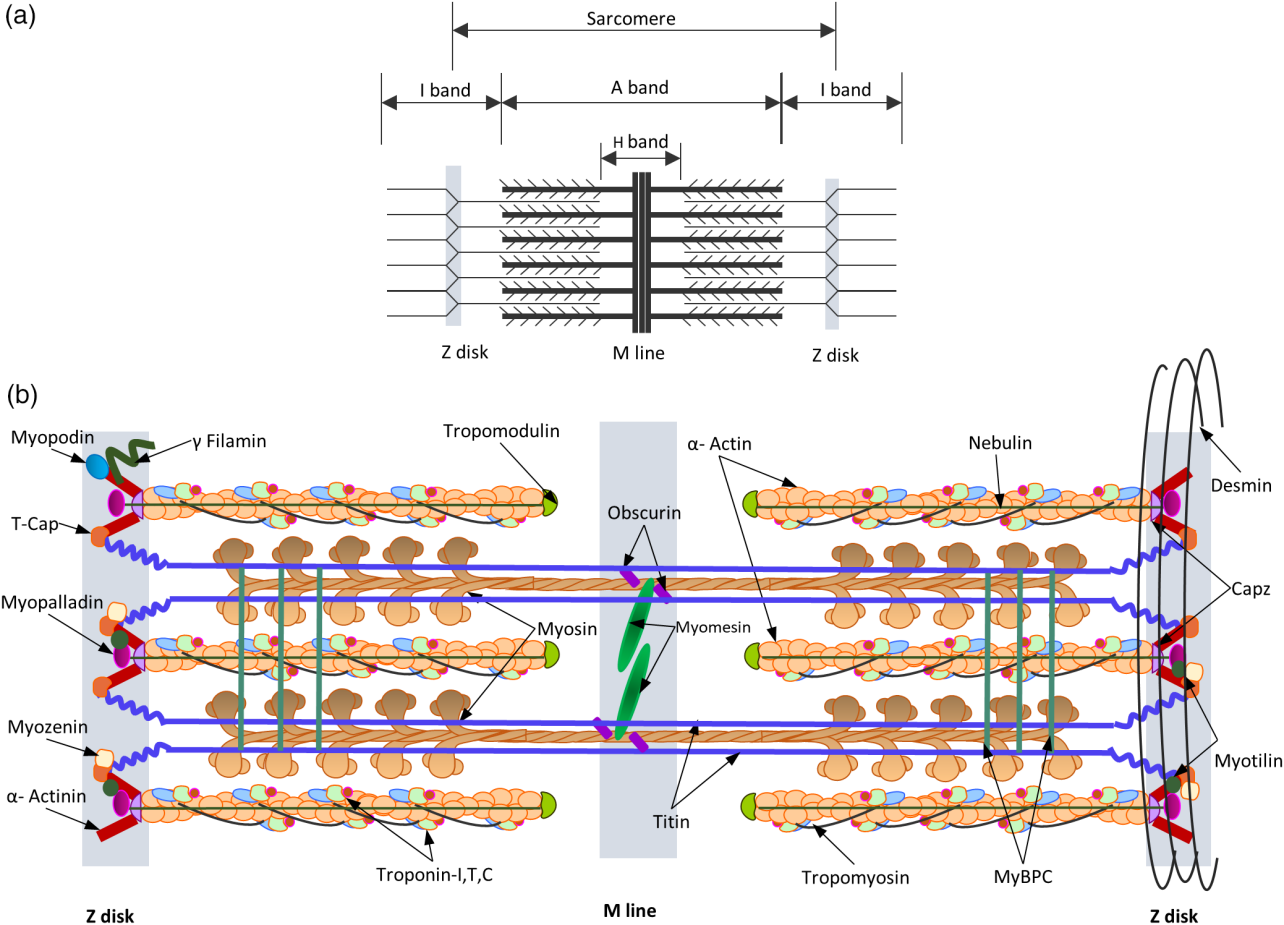
(a) Schematic representation of the striated skeletal muscle sarcomere showing the arrangement of thick and thin filaments in the sarcomere and identifying bands of overlap between them. (b) Schematic diagram of the sarcomere summarizing organization and location of major sarcomeric proteins. Cytosolic Ca^2+^ brings about a conformational change in the structure of troponin C, revealing myosin binding sites. Myosin heads successively bind and crawl along the length of actin, bringing about sarcomeric contraction. Titin and nebulin, function as “molecular templates” maintaining the length of the thick and thin filaments, respectively. A whole host of proteins within the M‐line and Z‐disk function mainly to maintain structural integrity of thick and thin filament lattices, respectively. The desmin intermediate filaments reinforce and integrate the structure of the muscle cell by forming transverse links between adjacent myofibrils

Force generation and rapid movement are hallmarks of striated muscle function brought about by contraction of the sarcomere. Sarcomeres represent an elegant piece of molecular machinery whose complex structure is composed of two main alternating sets of protein filaments: thin filaments (α‐actin and associated proteins) and thick filaments (myosin and associated proteins) which run parallel to the muscle fiber axis. Visually, the sarcomere is bordered at each end by a dark narrow line called the Z‐disk. Each Z‐disk bisects a lighter I band which is shared between adjacent sarcomeres. At the center of the sarcomere is a dense A‐band made up of thick filaments, with a lighter H‐zone. The M‐line bisects the H‐zone. Thin filaments are held together, in a lateral array, at the Z‐disk while the M‐band interconnects the thick filaments (Figure 8a; Huxley, 1957). Functionally, contraction begins with the binding of troponin‐C with the Ca^2+^ released during ECC. This brings about a conformational change in the troponin‐tropomyosin complex resulting in the exposure of myosin binding sites on the actin filaments Myosin heads then bind and crawl along the length of the actin filament bringing about hydrolysis of ATP and subsequently contraction (Huxley, 1969; Huxley & Kress, 1985).

The following sections briefly outline the major sarcomeric proteins, the mechanism of sarcomeric contraction, fiber types and their roles in health and disease (Clark, McElhinny, Beckerle, & Gregorio, 2002; Figure 8b).

#### 6.1.1 Thick filament

The thick filament is mainly composed of myosin proteins. Myosin is both an enzyme as it hydrolyzes ATP (head) and a structural protein (tail) and is associated with other non‐myosin proteins with specialized (mostly structural) functions such as myosin binding proteins (MyBPs) C and H of the M‐band. MyBPCs are an important class of MyBPs that contribute to myosin's precise organization and regulate force generation by the actomyosin complex (Ackermann & Kontrogianni‐Konstantopoulos, 2013). MyBPC is found associated with titin (Freiburg & Gautel, 1996) and as transverse stripes within the sarcomeric A‐band (R. Gilbert, Cohen, Pardo, Basu, & Fischman, 1999). The giant elastic protein, titin, extends along the length of the thick filament, as far as the Z‐line ensuring that equal forces are developed in the two halves of the A‐band in a mature muscle (K. Wang, McClure, & Tu, 1979). For a more detailed review of the titin gene and protein function, the reader is suggested a recent review by Linke (2018). Developmentally titin is suggested to act as a “molecular template,” a ruler, for defining the precise length and organization for myosin filaments (Horowits, Kempner, Bisher, & Podolsky, 1986).

As observed in Oldfors (2007), a new group of muscle diseases called “hereditary myosin myopathies” have emerged, associated mainly with myosin mutations. They broadly represent at least five different muscle diseases including myosin storage myopathy (MSM). MSM is a slowly progressing, relatively mild congenital myopathy characterized by accumulation of myosin in Type I muscle fibers. Other diseases included the Freeman–Sheldon and Sheldon–Hall syndromes as a result of MYH3 mutations, dominant inclusion body myopathy caused by mutations in fast myosin IIA and distal arthrogryposis trismus pseudocamptodactyly syndrome caused by mutations in perinatal MYH (reviewed in Laing & Nowak, 2005; Oldfors, 2007).

A whole class of proteins at the M‐band/M‐line, associate myosin with titin, which function to stabilize the transverse and longitudinal order of the thick filament lattice and link neighboring filaments for coordinated contraction of the sarcomeres (Hu, Ackermann, & Kontrogianni‐Konstantopoulos, 2015). Myomesin is one of the main proteins of the M‐line that are suggested to function as strain sensors within the sarcomere (Xiao & Gräter, 2014). Anti‐parallel dimers of myomesin link myosin filaments at the M‐line, and are linked in a ternary complex with obscurin and titin (Gautel & Djinović‐Carugo, 2016; Pernigo et al., 2015; Pernigo, Fukuzawa, Beedle, et al., 2017). Obscurin, serves as ligand for small ankyrin‐1, a protein integral to the network SR (Ackermann et al., 2011; Kontrogianni‐Konstantopoulos, Catino, Strong, et al., 2006; Kontrogianni‐Konstantopoulos, Jones, Van Rossum, & Bloch, 2003) and is suggested to regulate alignment of the network SR around the sarcomere (Kontrogianni‐Konstantopoulos et al., 2006). Creatine kinase, present in the M‐band binds to myosin and acts as spatial ATP buffer, essential for maintaining energy homeostasis and serving immediate ATP requirements of the sarcomere (Wallimann & Eppenberger, 1985; Wallimann, Schlösser, & Eppenberger, 1984). The presence of this protein kinase at the M‐band suggests an additional enzymatic role for the M‐band within the sarcomere. The M‐line also serves as a scaffold for a number of components of the protein turnover machinery via ubiquitin‐mediated turnover (Durham et al., 2006; Sarparanta et al., 2010) and is suggested to be involved in cytoskeletal remodeling (Hu et al., 2015).

#### 6.1.2 Thin filament

Actin isoforms polymerize to form thin filaments, an essential part of the contraction machinery. Similar to thick filaments, thin filaments are associated with a host of proteins that facilitate contraction. The most important are troponin (TNN‐I, the inhibitory subunit that binds to actin; TNN‐C, the calcium binding subunit and TNN‐T, the tropomyosin binding component) and tropomyosin that functions to stabilize actin and provide a molecular scaffold for positioning the Ca^2+^‐sensitive troponin molecule on the filament (reviewed in Zot & Potter, 1987). Ca^2+^ released upon fiber depolarization, raises the free Ca^2+^ concentration in cytosol, binding to Ca^2+^‐specific sites of TNN‐C, forming the initial signal for myofribrillar contraction, with changes propagating to TNN‐I/TNN‐T structure. These changes influence the troponin/tropomyosin and subsequently its interaction with actin, revealing sites for myosin binding on the actin filament (Galińska‐Rakoczy et al., 2008). Similar to titin, nebulin functions as a molecular template for thin filaments (Horowits et al., 1986). Tropomodulin, the capping protein for the pointed end of actin, prevents polymerization or depolymerization of actin thus maintaining the precise filament length necessary for efficient contraction (Gokhin, Ochala, Domenighetti, & Fowler, 2015).

Mutations in genes encoding skeletal muscle actin, tropomyosin, TNN‐T and nebulin result in molecular defects causative of a group of muscle disorders largely defined as congenital myopathies (particularly, nemaline rod myopathy). A detailed review, its clinical relevance and management is provided in Jungbluth et al. (2018) and Nance, Dowling, Gibbs, and Bönnemann (2012).

#### 6.1.3 Z‐disk

The Z‐disk/Z‐line anchors and cross‐links anti‐parallel actin filaments in a regular lateral array and connects repeating sarcomeres into the linear array of the myofibril. A large proportion of known sarcomeric proteins are identified within the Z‐disk including α‐actinin, myozenins, myotilin, myopalladin, myopodin, γ‐filamin, γ‐actin (Papponen, Kaisto, Leinonen, Kaakinen, & Metsikkö, 2009), muscle LIM protein (MLP), desmin, overlapping portions of thin filaments (nebulin, actin), titin and the more recently discovered NRIP protein (Chen et al., 2015; see Section 5).

α‐Actinin is a key structural component and cross‐linking protein of the Z‐disk. It also connects titin molecules from opposing sarcomere halves (Luther, 2009). Capping proteins for actin, CapZ (Yamashita, Maeda, & Maéda, 2003) and for titin‐telethonin/TCAP (Valle, Faulkner, De Antoni, et al., 1997; Zou et al., 2006), are located within the Z‐disk. Myopalladin (Bang et al., 2001) links nebulin to α‐actinin subsequently anchoring nebulin to the Z‐disk. It also interacts with titin and ANKRD1, suggesting a role in the stretch sensor system within the muscle. Myopodin, an actin bundling protein, co‐localizes with α‐actinin, γ‐filamin (Linnemann et al., 2010), synaptopodin 2‐like (Beqqali et al., 2010) and is suggested to participate in signaling between the nucleus and the Z‐disk during development and cellular stress. Myozenin binds to several Z‐disk proteins α‐actinin, γ‐filamin (Takada et al., 2001) and myotilin (Gontier et al., 2005) and is suggested to influence the dimerization and subsequent lateral spacing of thin filaments at the Z‐disk. Studies in exercise‐induced muscle remodeling have identified a translocation of myotilin from the Z‐disk to M‐bands (Carlsson, Yu, Moza, Carpén, & Thornell, 2007). MLPs are suggested to play role in mechano‐sensing (via costameric proteins; Flick & Konieczny, 2000) and actin dynamics (bundling and cross‐linking; Hoffmann et al., 2014).

Mutations in Z‐disk genes (myotilin, T‐cap, and titin) are associated with a form of dystrophy called limb‐girdle muscular dystrophy (LGMD; W.‐C. Liang & Nishino, 2015). LGMD are a genetically heterogeneous disease group, clinically characterized by progressive weakness of first proximal and then distal muscles. Myotilin, along with two other Z‐disk associated proteins, desmin and αB‐crystallin have also been implicated in myofibrillar myopathies characterized by abnormal myofibrillar degradation and accumulation of degradation products (Selcen & Engel, 2004).

In addition to the diseases specifically mentioned in the sections above, mutations in several sarcomeric proteins are also the cause for a major class of inherited diseases that affect cardiac mass and function called familial hypertrophic cardiomyopathy (FHC). Over 100 mutations have been identified in cardiac isoforms of thick, and thin filament proteins such as MYH7, TNNT2, TNNI3, TPM1, MYOZ2, MYL2, ACTC1, TCAP, MYBPC3, and TTN as contributing to FHC (reviewed in Bonne, Carrier, Richard, Hainque, & Schwartz, 1998; Marian, 2008).

### 6.2 Force generation

It is understood that muscle fibers have a consistent fiber diameter between muscles of different sizes and fiber size is directly proportional to fiber force generation. However, architecturally, how the myofibers arrange themselves with respect to the force‐generating axis demonstrates the versatility of muscle function. Three main classes of muscle architecture have been identified (Lieber & Friden, 2000): (a) longitudinal, where myofibers run along the length of muscle's force‐generating axis (e.g., biceps); (b) unipennate in which myofibers run along a fixed angle of the axis (e.g., vastus lateralis muscle); and (c) multipennate architecture in which muscle fibers run at several angles relative to the muscle's force‐generating axis (e.g., gluteus medius muscle).

**Figure 9:**
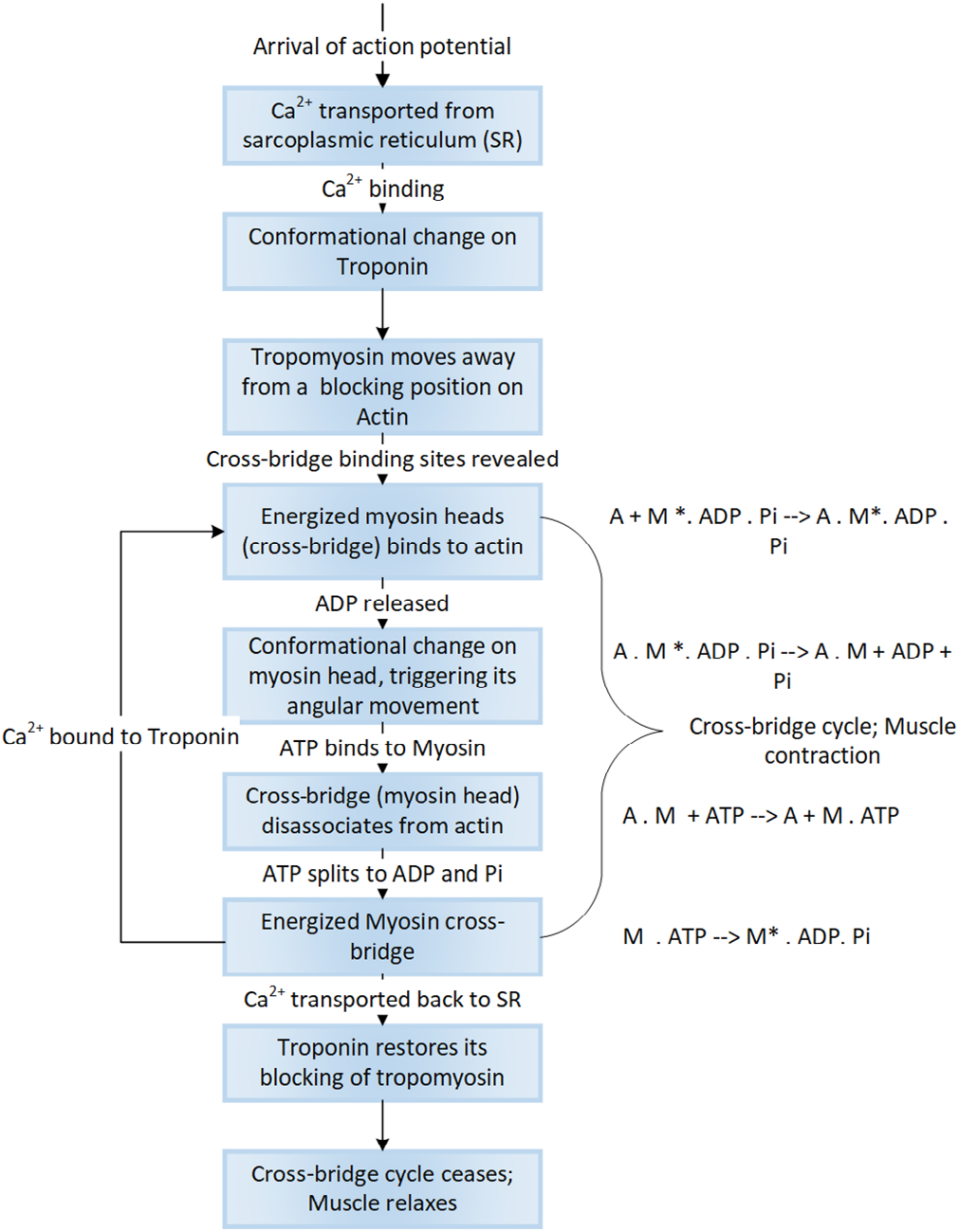
Cellular events underlying force generation. Force generation begins with arrival of an impulse, which changes the Ca^2+^ dynamics within muscle leading to a highly orchestrated set of specific changes to the molecular structure of the actomyosin complex bringing about sarcomeric contraction

At a molecular level, the sarcomeric contraction is a movement of the myosin heads on actin filaments—called cross‐bridge cycle. The cross‐bridge cycle is a sequence of enzymatic reactions responsible for movement of myosin heads on actin filaments, generating force within each individual myofibril, which is collectively experienced by the muscle. Briefly, force generation occurs in six steps and is summarized as follows (Fitts, 2008; Figure 9). At the onset of contraction, free cytosolic Ca^2+^ brings a conformational change in troponin, revealing myosin‐binding sites on actin filaments. Myosin head swings out towards the thin filament at a 45° angle and is in a rigor (stiff) state. Available ATP binds to myosin, briefly dissociating myosin from actin. The ATPase activity of myosin hydrolyzes ATP to ADP and Pi (free phosphate) (still bound to myosin) causing the myosin filament to weakly rebind actin at the 90° angle (cross‐bridge) relative to the actin filament. The release of Pi initiates the power stroke. The myosin head rotates on its hinge pushing the actin filament past it, towards the M‐band. At the end of the power stroke, myosin head releases ADP and regains its rigor state.

### 6.3 Fiber types

Force generation depends on the size and fiber type composition of skeletal muscle. Four types of muscle fibers (within two major fiber types) dominate skeletal muscle, namely slow‐twitch (Type I) and fast‐twitch (Type II) fibers containing subtypes IIA, IIB, and IIX. It is recognized that the pattern of Type II fiber specialization depends on expression patterns of myosin heavy chains isoforms during histogenesis (Rubinstein & Kelly, 2004).

Phenotypically, slow‐twitch or Type I muscle is highly vascularized and saturated with mitochondria and myoglobin exhibiting high mitochondrial and oxidative enzyme content with low glycolytic activity. Slow‐twitch fibers are resistant to fatigue, relying on oxidative metabolism for energy, while contracting for long periods with little force generated. Type I fibers are found more abundantly in elite endurance athletes (e.g., swimmers). Fast‐twitch or Type II muscle, exhibit faster contraction times, sustaining short anaerobic bursts of activity, fatiguing easier than Type I fibers. Type II fibers have a high glycolytic capacity ensuring adequate ATP generation to compensate for the accelerated rate of ATP hydrolysis. For this reason, a higher proportion of Type II fibers can be seen in elite strength and power athletes (e.g., sprinters, weight lifters). Of the three major subtypes (IIA, IIX, and IIB), that vary in both contractile speed and force generation. IIA fibers are similar to slow‐twitch in the sense that they have more myoglobin and depend more on oxidative metabolism.

Physiologically, the difference between fast‐ and slow‐twitch muscles is based on differences in their calcium kinetics, ECC mechanisms, and molecular motor activity, which governs the basic twitch parameters (time to peak tension and half‐relaxation time). Fast fibers exhibit shorter twitch parameters, and rapid contraction of the sarcomere. Fast fibers allow for generation of fast and large calcium transients, contributed by lower cystolic‐free Ca^2+^, reduced Ca^2+^ entry from extracellular space, and greater abundance of RYRs and SERCA pumps (Reggiani & Te Kronnie, 2006). Fast fibers are endowed with a powerful contractile machinery primarily due to differing myosin isoforms (MYH2 in IIA, MYH4 in IIB, and MYH1 in IIX fibers, respectively) exhibiting rapid sarcomeric shortening velocity and higher mechanical power. Slow fibers contract much more slowly, generating less mechanical power with lesser ATP expenditure, making them (fiber subtypes) metabolically diverse (Rivero, Talmadge, & Edgerton, 1998).

Genetically, each muscle fiber type is equally diverse with different thick and thin filament isoforms being expressed in slow and fast muscle. For instance, MYH7, MYL2/3, MYBL2, TNNT1/I1/C1, TPM3, TMOD1, ATP2A2, and CASQ2 represent slow fiber isoforms, while MYL1, MYBP2, TNNT3/I1/C2, TPM1, TMOD4, ATP2A1, CASQ1 all represent fast fiber isoforms.

#### 6.3.1 Fiber‐type remodeling and the effect of exercise on fiber types

Skeletal muscle fibers exhibit remarkable plasticity, an ability to undergo adaptive changes, in response to physical activity (exercise) or inactivity (disuse, disease, injury). Studies have identified mechanisms necessary for specifying fiber type during development and maintaining or switching fiber types thereafter. For instance, Buller, Mommaerts, and Seraydarian (1969) first demonstrated fiber type switching in cats as a result of changes in nerve activity. The role of exercise in fiber‐type remodeling and muscle function is well studied in the context of sports physiology (Wilson et al., 2012), and, is of importance in metabolic diseases and cardiovascular health. For instance, exercise in human and animal models is shown to induce a switch in fiber types to a more oxidative fast fiber phenotype (IIX → IIA in humans, and IIB → IIX → IIA in rats and mice with a nonsignificant switch to a slow phenotype (Ausoni, Gorza, Schiaffino, Gundersen, & Lomo, 1990). Fiber‐type switching has been evidenced to involve signaling mechanisms containing the calcineurin‐NFAT signaling pathway as reported in a seminal paper by Chin et al. (1998); bidirectional promoters (which can generate both sense and antisense transcripts located in the vicinity of MYH genes; Rinaldi et al., 2008), and/or miRNAs (located within the MYH genes; van Rooij et al., 2009). MYH gene expression and fiber type profile (as a result of disease or exercise) are also known to be affected by the activity of genes such as MEF2 (H. Wu et al., 2000), PPAR‐β/δ (Schuler et al., 2006; Y.‐X. Wang et al., 2004), activated protein kinase (AMPK; Lee‐Young, Canny, Myers, & McConell, 2009), and PGC1‐α (Handschin et al., 2007; Lin et al., 2002).

Studies in various animal and human models of disease and injury have additionally shown that both skeletal and cardiac muscle fibers begin to express embryonic and developmental isoforms, such as MYH3 and MYH8 (Mukund & Subramaniam, 2015; Mukund, Ward, Lieber, & Subramaniam, 2017; Taegtmeyer, Sen, & Vela, 2010), possibly contributing to observed changes in muscle force and resistance to fatigue. A detailed and versatile review on the functional, physiological and mechanistic differences between muscle fiber types is provided in Schiaffino and Reggiani (2011).

## 7 EXTRACELLULAR MATRIX

Connective tissue of muscle is a complex entity, comprising of non‐contractile ECM with embedded fibroblasts and macrophages and an extensive network of capillaries and nerves, flexible enough to adjust to contraction–relaxation cycles. ECM is multifunctional within muscle and enables uniform distribution and transmission of force within muscle and from muscle to tendon (along BL). ECM also serves as a scaffold for cell matrix interactions (focal adhesion) necessary for a host of biological responses within the muscle (Grzelkowska‐Kowalczyk, 2016). The cytoskeleton‐ECM‐reticular linkage (via the dystrophin associated protein complex, DAPC) has been shown to be crucial for providing necessary biomechanical support and handling contraction (stretch) stresses within the muscle, behaving as a key modulator for maintaining mechanical homeostasis within the muscle (Humphrey, Dufresne, & Schwartz, 2014).

Traditionally, ECM in skeletal muscle is organized into three discrete but interconnected structures: epimysium, perimysium, and the endomysium. The epimysium, a dense connective tissue layer encapsulates the entire muscle while the perimysium derives from the epimysium and surrounds the fascicles (bundles of muscle fibers). The endomysium or basement membrane (comprising of an inner BL [adjacent to the sarcolemma] and an outer reticular lamina) is a delicate layer of ECM surrounding each myofiber. This gross classification is in much debate given our increasing recognition of the ECM complexity in structure and function (Gillies & Lieber, 2011).

**Figure 10:**
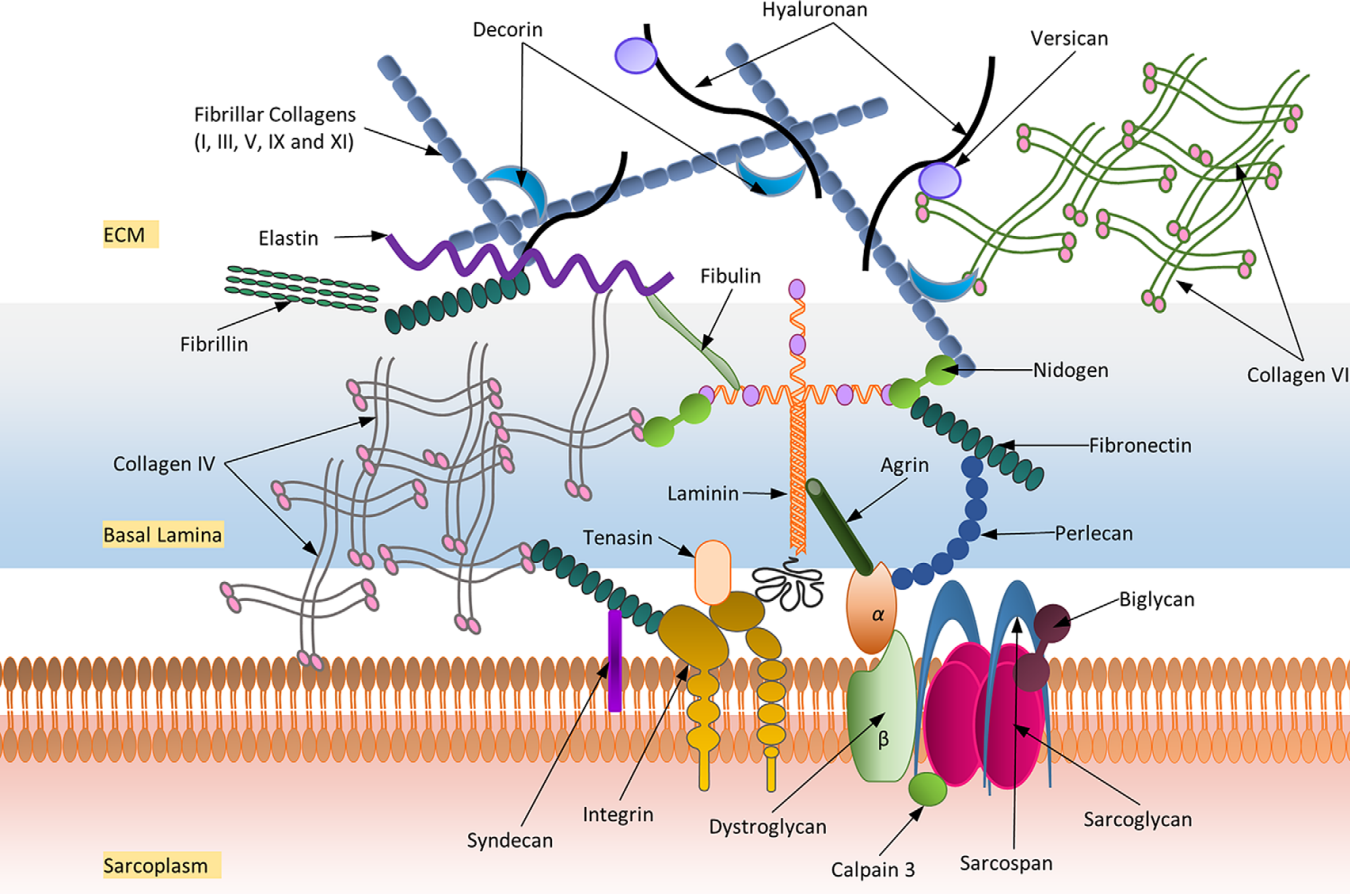
A schematic representation of the main extracellular matrix proteins and their approximate localization surrounding skeletal muscle

The ECM constitutes three main classes of proteins namely collagens, non‐collagenous glycoproteins and PGs (Figure 10). Collagens represent the largest fraction of matrix proteins within the muscle (Gelse, Pöschl, & Aigner, 2003; Gordon & Hahn, 2010). Collagens I, III, V, and XI are fibrillar collagens that are capable of forming fibrils in the muscle ECM. Collagen I, a major collagen within muscle, exhibits considerable biomechanical properties including tensile strength and load bearing. Collagen VI, a microfibrillar protein forms a network of fine filaments, while collagen IV forms the most important structural component of the basement membrane integrating laminins, nidogens (Fox et al., 2008; Ho, Böse, Mokkapati, Nischt, & Smyth, 2008), and other proteins into a stable structure. Lysyl hydroxylase‐3 plays a particularly important role in the biosynthesis of functional collagen types IV and VI (Salo et al., 2008). Fibronectin functions as a “master organizer,” aiding in fibril organization along with fibrillin‐1 and as a bridge between proteins including integrins (α7/β1), collagen IV, PGs and other focal adhesion molecules (Halper & Kjaer, 2014). It also plays an essential role in the assembly of fibrillin‐1 into structured microfibrils (Sabatier et al., 2009). Elastin is the main component of elastic fibers (encased in layers of microfibrils and PGs) contributing to muscle elasticity (A. Gilbert, Wyczalkowska‐Tomasik, Zendzian‐Piotrowska, & Czarkowska‐Paczek, 2016; Kozel, Ciliberto, & Mecham, 2004).

A variety of regulatory ECM proteins are involved in matrix assembly and the modulation of cell–matrix interactions, including nidogens, periostin, and SPP1. MMPs and their inhibitors (TIMP1, TIMP2) are an important class of ECM‐associated enzymes that maintain ECM integrity and regulate ECM protein degradation (Arpino, Brock, & Gill, 2015; S. Murphy & Ohlendieck, 2016). A variety of PGs (such as hyaluronan), chondroitin sulfate/dermatan sulfate PGs (such as versican; Nandadasa, Foulcer, & Apte, 2014), small leucine‐rich repeat PGs (e.g., biglycan, decorin, lumican, fibromodulin) and HS PGs (e.g., syndecan, perlecan, agrin) have been identified to be distributed between the collagen fibers (Halper & Kjaer, 2014). HA is a large, linear glycosaminoglycan highly expressed in muscle during development (Tammi et al., 2011). Biglycan interacts with α‐sarcoglycan and γ‐sarcoglycan (Bowe, Mendis, & Fallon, 2000), while decorin, a known inhibitor of TGF‐β, is the primary PG molecule of the perimysium (J. Zhu et al., 2007). Syndecan, perlecan, and agrin are found associated with the basement membrane and co‐operate with integrins to facilitate cell–ECM interactions (Sarrazin, Lamanna, & Esko, 2011). Several of the above‐mentioned proteins serve as important signaling mediators that directly influence muscle regeneration, wound healing and recovery (Aya & Stern, 2014; Y. Li et al., 2007; Schultz & Wysocki, 2009). Production and maintenance of these ECM components is tightly regulated by a host of growth factors sequestered at the ECM including connective tissue growth factor (regulates collagen gene expression), HGFs (regulate quiescent satellite activation), FGFs (stimulate angiogenesis and regulate fibroblast proliferation and action), and TGFβ (regulate fibroblast and ECM expression) (Flaumenhaft & Rifkin, 1991). ECM at the NMJ plays a crucial role in the organization and interaction between the nerve terminal and muscle fiber as outlined in Section 4.2.

### 7.1 ECM in pathology

Muscle is capable of regenerating and should ideally recover completely upon injury. However, muscle ECM composition and function are dramatically affected after chronic/acute injury arising from disease (Carmignac & Durbeej, 2012), diet (Tam, Power, Markovic, et al., 2015), poisons/pathogens (Mukund et al., 2017), Crum‐Cianflone (Crum‐Cianflone, 2008), and age (Stearns‐Reider et al., 2017). Insulin resistance, the hallmark of diabetes, is tightly linked with ECM remodeling and deposition of ECM proteins such as collagens, laminins and fibronectin, predisposing diabetes (Ban & Twigg, 2008; Fukui et al., 1992; A. S. Williams, Kang, & Wasserman, 2015). Studies stimulating chronic/acute muscle and nerve injury have repeatedly identified ECM expansion as a crucial step in muscle recovery, particularly at the BL. Mutations of laminin α2 and collagen VI of the synaptic BL have more recently been identified to be causative of congenital muscular dystrophy (Muntoni & Voit, 2004). In models of chronic/acute injury, rapid fiber necrosis is observed immediately upon injury, resulting in the activation of the complement cascade and infiltration of leukocytes and neutrophils followed by monocytes (macrophages). Phagocytic macrophages clear damaged myofibers and produce anti/pro‐inflammatory cytokines such as TGFβ and TNFα, which regulate cell migration, proliferation and muscle regeneration (Philippou, Maridaki, Theos, & Koutsilieris, 2012). In muscle recovery, resident fibroblasts are transformed into myofibroblasts (which synthesize ECM components such as fibrous collagens I and III and BL collagens IV and VI; Chapman, Mukund, Subramaniam, Brenner, & Lieber, 2017), bringing about an expansion of ECM proteins. Several myopathies and dystrophies are associated with mutations in several ECM genes such as decorin, perlecan, syndecan (Van et al., 2017) as outlined in Supplementary Table 2.

#### 7.1.1 Fibrosis

In most pathologies, the initial ECM expansion process becomes uncontrolled, leading to a substantial remodeling of muscle ECM. This uncontrolled and irreversible ECM expansion accompanied by an accumulation of ECM due to inhibited degradation (turnover), results in a fibrotic phenotype within muscle (Mann et al., 2011), especially in chronic diseases such as dystrophinopathies (Serrano & Muñoz‐Cánoves, 2017). The chronic and sustained inflammatory response in dystrophic muscle serves as a positive feedback mechanism prolonging macrophage activity, release of inflammatory cytokines and increased ECM production (Serrano & Muñoz‐Cánoves, 2010). We have previously also shown evidence for fibrosis in muscle injected with botulinum neurotoxin A (Mukund et al., 2014).

TGFB1, a secreted cytokine of M2 (anti‐inflammatory) macrophages is a crucial regulator of fibroblast activity and collagen synthesis and accumulation in wound healing and repair (Biernacka, Dobaczewski, & Frangogiannis, 2011). Though the precise molecular mechanism of TGFβ action on fibroblasts is yet to be understood, it is suggested to stimulate transition of resident fibroblasts into myofibroblasts (key effector cells for ECM production, and in pathology fibrosis), via the SMAD pathway (Evans, Tian, Steadman, & Phillips, 2003) and in a SMAD independent manner involving PI3K/AKT pathway (Conte et al., 2011; Wilkes, Mitchell, Penheiter, et al., 2005). Myostatin has been shown to directly influence fibrosis and fibroblast activation via the p38MAPK and AKT pathways (Z. B. Li, Kollias, & Wagner, 2008). The myofibroblast phenotype is characterized by formation of gap junctions and the expression of α‐smooth muscle actin (incorporated into the newly formed contractile bundles imparting contractility and facilitating repair), fibronectin and non‐muscle myosin (MYH10) (Baum & Duffy, 2011). Recent studies in cardiac and skeletal muscle have identified scleraxis (SCA), a transcription factor, as being critical for regulating expression of resident fibroblasts and myofibroblasts (Bagchi et al., 2016; Mendias et al., 2012).

Additionally, mesenchymal transition of fibro/adipogenic progenitor (FAP) in regenerating/degenerating fiber microenvironments has been implicated in contributing to an activated fibroblast population (Joe, Yi, Natarajan, et al., 2010; Uezumi, Fukada, Yamamoto, Takeda, & Tsuchida, 2010; Uezumi, Ikemoto‐Uezumi, & Tsuchida, 2014). In addition to the well accepted role of macrophages in muscle regeneration (Tidball & Villalta, 2010), a more recent study highlighted their role in “directing” muscle fate between regeneration and fibrosis, by maintaining a balance between apoptotic TNFα (from M1 macrophages) and anti‐inflammatory TGFβ (TGFB1, from M2 macrophages) (Lemos, Babaeijandaghi, Low, et al., 2015). This balance appears to be essential for maintaining FAP population homeostasis in regenerating/degenerating fiber microenvironment (Muñoz‐Cánoves & Serrano, 2015). Briefly, the sequence of expression with an early wave of TNFα expression followed by a later wave of TGFβ is crucial for healthy muscle regeneration. A loss of this sequential progression under acute/chronic inflammatory conditions causes elevated TGFβ which stimulates differentiation of FAPs into fibroblasts contributing to fibrotic phenotype.

## 8 CYTOSKELETON

**Figure 11:**
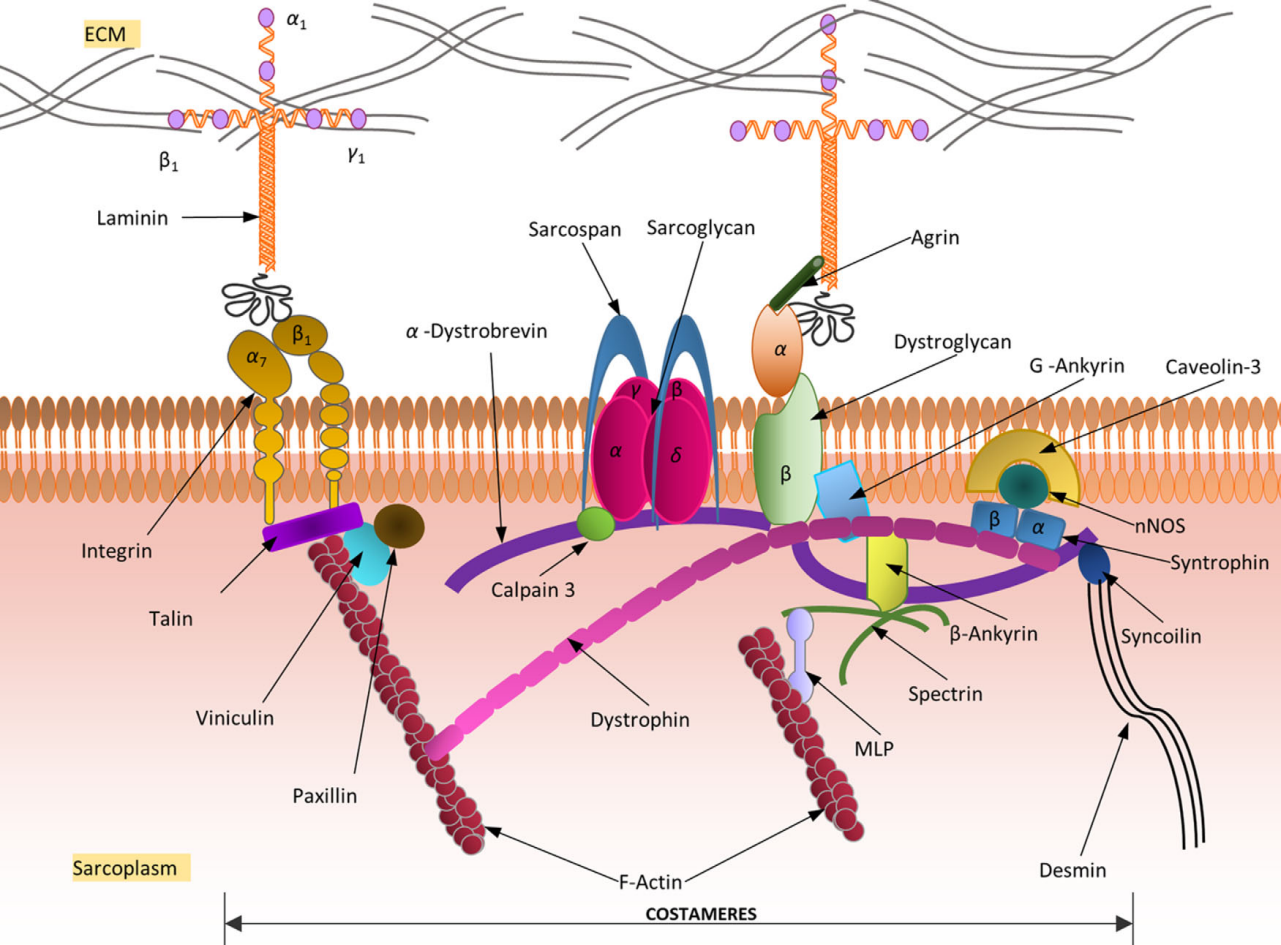
A schematic representation of the main cytoskeletal proteins associated with skeletal muscle. The dystrophin‐associated protein complex (DAPC) is a group of sarcoplasmic (α‐dystrobrevin, syntrophins, and nNOS), transmembrane (β‐dystroglycan, sarcoglycans, caveolin‐3, and sarcospan) and extracellular proteins (α‐dystroglycan and laminin), linking dystrophin to the extracellular matrix (ECM). Dystrophin also links to desmin, an important sarcolemma integrity protein, via the α‐dystrobrevin‐syncoilin interaction, providing a strong mechanical link between the intracellular cytoskeleton and the extracellular matrix

The plasticity of muscle, that is, the ability to not self‐destruct after repeated stresses of contraction and relaxation, can be attributed to the complex, and yet‐to‐be fully understood muscle cytoskeleton. The muscle cytoskeleton serves as the structural and supportive scaffold for sarcomeres within the muscle. The cytoskeletal framework consists of the following major components (Figure 11): (a) a sub‐sarcolemmal network that mediates attachment of several cytoskeletal proteins to the sarcolemma; (b) a transverse connecting system anchored to the sub‐sarcolemmal network; (c) the protein complex that connects the ends of the myofibrils to the sarcolemmal folds at the myotendinous junction and longitudinally arranged microtubules running parallel and in between the myofibrils.

Dystrophin is a large protein that serves to maintain synchronous stretch and contractions by anchoring the sarcomere (via actin filaments) to the sarcolemma (via the BL) of the muscle (Hoffman, Brown, & Kunkel, 1987). Duchene muscular dystrophy (DMD‐d) and Becker muscular dystrophy represent two major dystrophinopathies that are caused due to mutations (frameshift in the former case) resulting in aberrant dystrophin expression causing asynchronous stretching of the sarcomere and tears in the sarcolemma (Mah et al., 2014). Studies in dystrophic animal models with mutated dystrophin have shown an overexpression of utrophin, a protein similar to dystrophin in structure and function probably as a compensatory mechanism for reduced dystrophin functionality (Hirst, McCullagh, & Davies, 2005). Dystrophin is part of a large group of proteins DAPC containing sarcoplasmic (signaling) proteins (α‐dystrobrevin, syntrophins and neuronal nitric oxide synthase [nNOS]), mechanical support proteins that are transmembrane (β‐dystroglycan, the sarcoglycans, caveolin‐3, and sarcospan) and extracellular (α‐dystroglycan and laminin; Constantin, 2014).

The costamere forms a critical component of striated muscle morphology connecting (or “bolting”) the sarcomeres to the sarcolemma (Peter, Cheng, Ross, Knowlton, & Chen, 2011). Costameres comprise of two groups of interacting proteins, both anchored on cytoskeletal F‐actin filaments, one containing the DAPC and the other containing the integrin (α7/β1) and its associated proteins talin, viniculin, and paxillin. An ankyrin‐based mechanism for sarcolemma localization of dystrophin and β‐dystroglycan has been evidenced (Ayalon, Davis, Scotland, & Bennett, 2008), with ankyrin‐G being required for retention of both proteins to at the costameres (Tee & Peppelenbosch, 2010). Spectrin‐B2 is required for the association of β‐ankyrin with dystrophin at the costameres (Ayalon et al., 2011). Spectrin‐B2 also interacts with MLP (Z‐disk protein; Flick & Konieczny, 2000). Myofibrils are exposed to, and have to withstand, both axial and lateral forces during active contraction. The IF network is responsible for maintaining fiber integrity and lateral force transmission. IFs form a sheath surrounding each myofibril at that Z‐disk and connect the transverse cytoskeletal network with the sarcolemma. Desmin (mature muscle isoform) and vimentin (immature muscle isoform) are the major proteins of IF in a healthy muscle (Paulin & Li, 2004). Desmin mutations are associated with forms of familial myofibrillar myopathies (Goldfarb, Vicart, Goebel, & Dalakas, 2004; Selcen, 2011) and cardiomyopathies (Harada et al., 2018). Smaller quantities of other IF proteins nestin/paranemin, syncolin, and synemin/desmuslin connect the IF network with edges of Z‐disk. Various plectin isoforms (PLEC, 1f, 1, 1d and 1b) have been suggested to link desmin IF (DIF) with the thin filaments, mitochondria and nucleus within muscle (Castanón, Walko, Winter, & Wiche, 2013). The costameres and DIF together form the transverse fixation system of muscle.

Plectin deficiency results in epidemyolysis bullosa simplex, a class of congenital diseases characterized by dermal–epidermal separation leading to skin blistering, co‐manifested in many cases by muscular dystrophy (Winter et al., 2016) and blistering of the gastrointestinal tract (pylori atresia; Natsuga et al., 2010). Mutations of proteins associated with the transverse fixation system causes a loss in sarcolemmal integrity making muscle vulnerable to stresses leading to various types of muscular dystrophies or myopathies (Jaka, Casas‐Fraile, de Munain, & Sáenz, 2015). In most cases, the subcellular localization of the affected protein correlates with disease severity.

### 8.1 Cytoskeletal signaling

Two proteins of the DAPC, syntrophin and α‐dystrobrevin, are suggested to have a signaling role over a structural one, within muscle, in the presence of dystrophin. In the absence of these proteins, nNOS (a nitric oxide synthase) is displaced from the sarcolemma to the sarcoplasm. Recent studies suggest that aberrant nNOS signaling can negatively impact three important clinical features of dystrophinopathies and sarcoglycanopathies: maintenance of muscle bulk, force generation and fatigability (Percival, Anderson, Gregorevic, Chamberlain, & Froehner, 2008). Likewise, nNOS overexpression studies have shown an amelioration of the dystrophic phenotype perhaps owing to the anti‐inflammatory properties of nNOS (Wehling, Spencer, & Tidball, 2001). Syntrophin links to ECM via dystrophin in the DAPC, and is thought to regulate kinases, ion channels and several signaling protein cascades emphasizing its role in creating signal‐transduction complexes with the DAPC (Constantin, 2014). Additionally, DIF has been recently relegated a regulatory role, forming a stress‐transmitting, stress‐signaling network during high stress, and is associated with stress‐mediated JNK signaling within the muscle (Palmisano et al., 2015).

## 9 MUSCLE ATROPHY AND HYPERTROPHY

**Figure 12:**
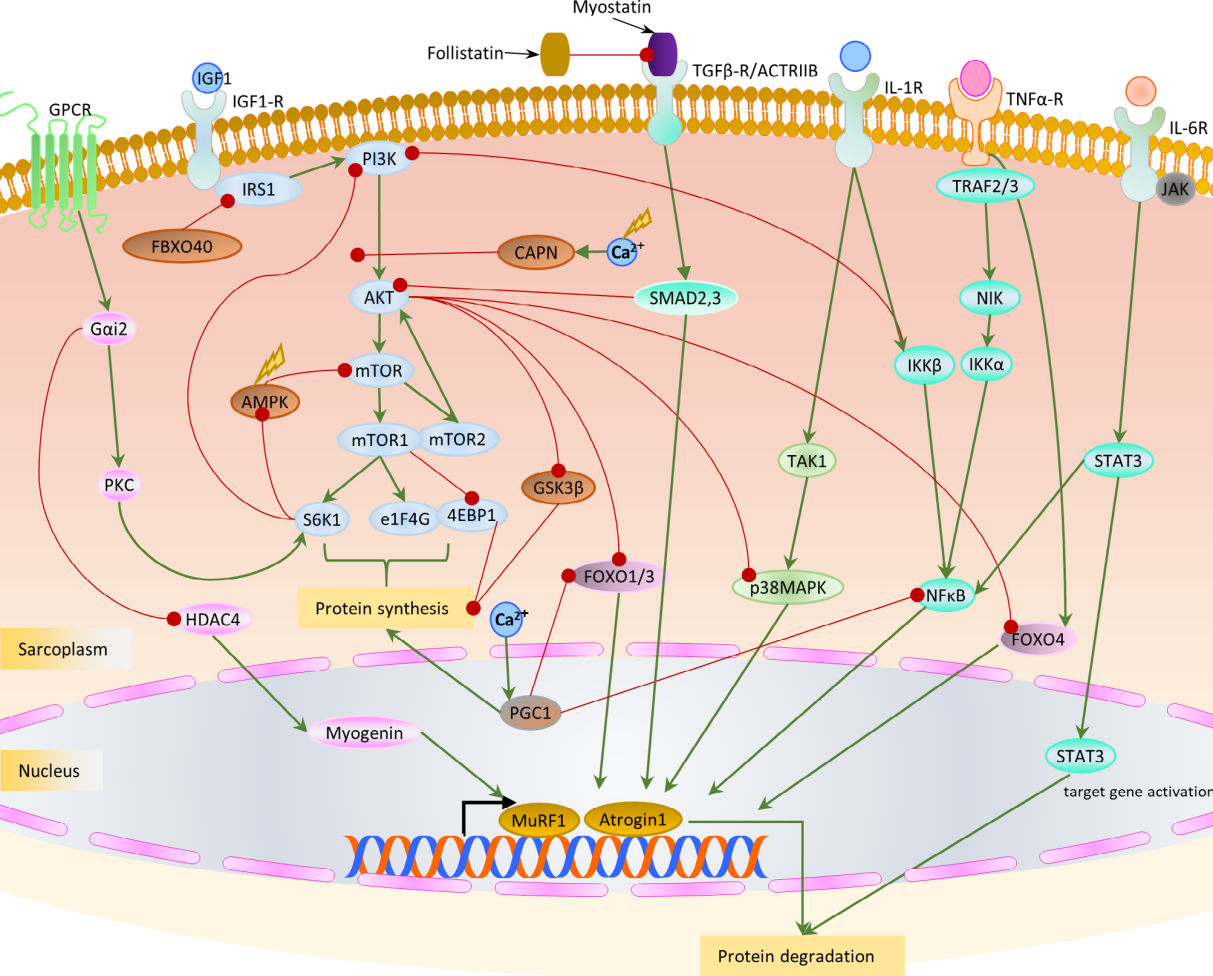
Schematic representation of the major receptors and signaling pathways/proteins involved in atrophy and hypertrophy. We identify only the major molecular actors within each pathway for the sake of simplicity. The IGF/AKT pathway forms a crucial pathway for hypertrophy in muscle. While activation of MURF1/Atrogin1 via the SMAD, NFκB and STAT signaling lead to atrophy

A dynamic balance between the rate of synthesis and degradation of contractile proteins establishes the health of the muscle fiber. A shift in this balance leads to visible changes in composition, appearance and performance of the muscle fiber and is caused due to factors internal and external to the muscle, such as GFs, inflammation (Haddad, Zaldivar, Cooper, & Adams, 2005; Jackman & Kandarian, 2004), oxidative stress and muscle disuse (Powers, Kavazis, & McClung, 2007), exercise (LaPier, 1997), steroids (Yu et al., 2014), and disease (Bailey, Zheng, Hu, Price, & Mitch, 2006; Doucet et al., 2007). The major signaling pathway that regulates muscle mass and protein synthesis is the IGF1‐Akt/PKB‐mTOR signaling pathway (Schiaffino & Mammucari, 2011; Figure 12). Briefly, signaling via IGF1 begins with IGF1 ligand binding to its receptor (IGF1R), which results in the recruitment of insulin receptor substrate (IRS1). IRS1 in turn activates P13K to produce phosphatidylinositol‐3,4,5 triphosphates (PIP3) via PIP2 phosphorylation. PIP3 activates AKT proteins which primarily function to promote protein synthesis and cell growth via direct phosphorylation and activation of its downstream target mammalian target of rapamycin (mTOR). Activated mTOR complexes with protein RPTOR to form mTOR complex 1 (mTORC1), and RICTOR to form mTOR complex 2 (mTORC2). mTORC1 positively regulates the activation of its downstream targets S6K1 and negatively the inhibitor of eIF4E‐4EBP1 leading to increased protein translation and synthesis. Other downstream targets of AKT include the glycogen synthase kinase 3b (GSK3β) and forkhead box O (FOXO) transcription factors. Inhibition of GSK3β by AKT relieves inhibition of the initiation factor eIF2B increasing protein synthesis.

Increased activation of IGF1‐AKT‐mTOR signaling pathway is one of the main causes for increased muscle bulk, via SC activation leading to muscle hypertrophy. Clinically, hypertrophy is characterized by both an increase in myocyte number (hyperplasia) and size. Hypertrophy is characteristic of a clinically strong and healthy “exercised” muscle (e.g., in athletes) and in pathology such as myotonia congenita (Varkey & Varkey, 2003). In addition to the classical IGF1‐AKT‐mTOR pathway, hypertrophy in healthy muscle has been shown to be induced due to SC activation via G‐protein coupled receptors, specifically via the α‐subunit Gαi2 (Minetti et al., 2011, 2014) and through myostatin inhibition (Amthor et al., 2009; X. Zhu, Hadhazy, Wehling, Tidball, & McNally, 2000). Gαi2 can bypass AKT in both a PKC‐dependent and HDAC4‐dependent manner, perturbing GSK3β and S6K1 signaling downstream of mTOR (Minetti et al., 2014). Recent knockdown studies of MRF4 have shown induction of muscle growth (hypertrophy) via activation of MEF2 and its downstream targets (Moretti et al., 2016). Aerobic exercise has been demonstrated to acutely and chronically alter protein metabolism and induce skeletal muscle hypertrophy (Konopka & Harber, 2014).

When the synthesis versus degradation balance shifts increasingly towards protein degradation in response to a variety of stimuli, including viral and bacterial infection, exposure to pro‐inflammatory cytokines, mitogens, GFs, and oxidative and biomechanical stresses, the muscle atrophies. Atrophying muscle is characterized by a wasting or a loss of muscle mass accompanied by a decrease in the cross‐sectional area of the muscle fiber, the muscle volume, and the amount of muscle protein (Boonyarom & Inui, 2006; Jackman & Kandarian, 2004). Muscle atrophy can occur for various reasons such as disease, injury, and extended periods of immobility (e.g., limb immobilization or even space flight). Four major proteolytic systems, namely, lysosomal (autophagic), proteasomal, calpains and caspases, become activated and contribute to atrophy depending on various environmental and cellular cues. Several studies have focused on systematically decoding the gene expression signatures for protein degradation in the atrophying muscle via the ubiquitin‐proteasome system (Bodine et al., 2001; Gomes, Lecker, Jagoe, Navon, & Goldberg, 2001; Lecker, Jagoe, Gilbert, et al., 2004; Sandri et al., 2004). Atrogin‐1 and MURF1 represent two ubiquitin E3 ligases of the ubiquitin‐proteasome system, with initiation factor eIF3f and myosin chains as main substrates, respectively (Foletta, White, Larsen, Léger, & Russell, 2011). They are largely considered “master regulators” of muscle atrophy (Bodine & Baehr, 2014). Expression of these two regulators in muscle depends on the translocation and activity of FOXO transcription factors, which are controlled via the AKT pathway (Sandri et al., 2004; J. Zhao et al., 2007). Specifically, reduced AKT activation in atrophic muscle permits phosphorylation and translocation of FOXO to the nucleus, sufficient to promote protein breakdown via the increased expression of atrogin‐1 and MURF1; while genetic activation of AKT is evidenced to be sufficient to initiate hypertrophy, it is crucial for weight recovery after atrophy (Sandri et al., 2004; Stitt et al., 2004). Myostatin, a secreted molecule of the TGFβ family acts to limit muscle growth (via the MURF1‐independent activation SMAD2/3 pathway; Sartori et al., 2009) in healthy muscle and is over‐expressed in certain forms of atrophies and hypertrophies (Rodriguez et al., 2014). Myostatin inhibition is currently being pursued as a potential therapy for certain myopathies (Y.‐S. Lee et al., 2015).

Pro‐inflammatory factors, particularly factors such as interleukin (IL‐1) and TNFα also upregulate the expression of the two key E3 ligases, MURF1 and atrogin‐1, signaling through two established pathways of p38MAPK (Y.‐P. Li et al., 2005) and NFκB (Jackman, Cornwell, Wu, & Kandarian, 2013) bringing about muscle atrophy. Independent activation of atrogin‐1 in the presence of a pro‐inflammatory cytokine TNFα via its action on FOXO4 (Moylan, Smith, Chambers, McLoughlin, & Reid, 2008, p. 4) has been reported. Chronic, low‐level increase in circulating interleukin (IL‐6) is observed in several disease states and exercised muscle. IL‐6, unlike IL‐1 has been suggested to induce atrophy through a negative feedback mechanism by controlling the STAT3 phosphorylation state (and its translocation to nucleus to activate its downstream targets) contributing to a more catabolic state in the muscle (via the JAK/STAT (Haddad et al., 2005) pathway and in an NFκB‐dependent manner (Ma et al., 2017)). Recent work has highlighted an amelioration of denervation‐induced atrophy by inhibiting IL‐6‐STAT3 signaling in FAPs (Madaro et al., 2018; Marazzi & Sassoon, 2018), further emphasizing the influence of immune signaling on muscle atrophy. Another ubiquitin ligase E3, FBXO40, is shown to initiate atrophy in denervated muscle ubiquitinating IRS1, thus inhibiting the downstream PI3K/AKT pathway (Ye, Zhang, Xu, Zhang, & Zhu, 2007). Loss of Ca^2+^ homeostasis due to increased oxidative stress (Smuder, Kavazis, Hudson, Nelson, & Powers, 2010) or in diseases such as muscular dystrophy (Tidball & Spencer, 2000), can activate non‐lysosomal Ca^2+^‐regulated proteases called calpains (R. M. Murphy, 2010). In muscle wasting, calpain‐3 proteolysis occurs via the AKT pathway, and is suggested to act on several cytoskeletal proteins such as titin, desmin and α‐actinin, the actomyosin complex—preferentially at the Z‐disk, contributing to atrophy and fiber necrosis (Bartoli & Richard, 2005; Huang & Zhu, 2016). Contradictory to the role of ubiquitous calpains, inactivation of calpain 3 leads to muscular dystrophy and its complete lack is associated with a type of LGMD‐2A (Saenz, Leturcq, Cobo, et al., 2005). Inflammatory cascades, beginning with activation of interleukins (see Section 10.1) acting via the NFκB signaling pathway, also lead to muscle atrophy. Caspases, a set of apoptotic enzymes, specifically caspase‐3 are overexpressed in certain atrophies of the muscle (e.g., denervation atrophy, DMD‐d) (Du, Wang, Miereles, et al., 2004; Sandri, El Meslemani, Sandri, et al., 2001).

As seen above, atrophy and hypertrophy occur as response to a variety of inflammatory stimuli, oxidative/biomechanical stresses inextricably linked to the health of muscle segueing into our next section on inflammation and oxidative stress.

## 10 INFLAMMATION AND OXIDATIVE STRESS

**Figure 13:**
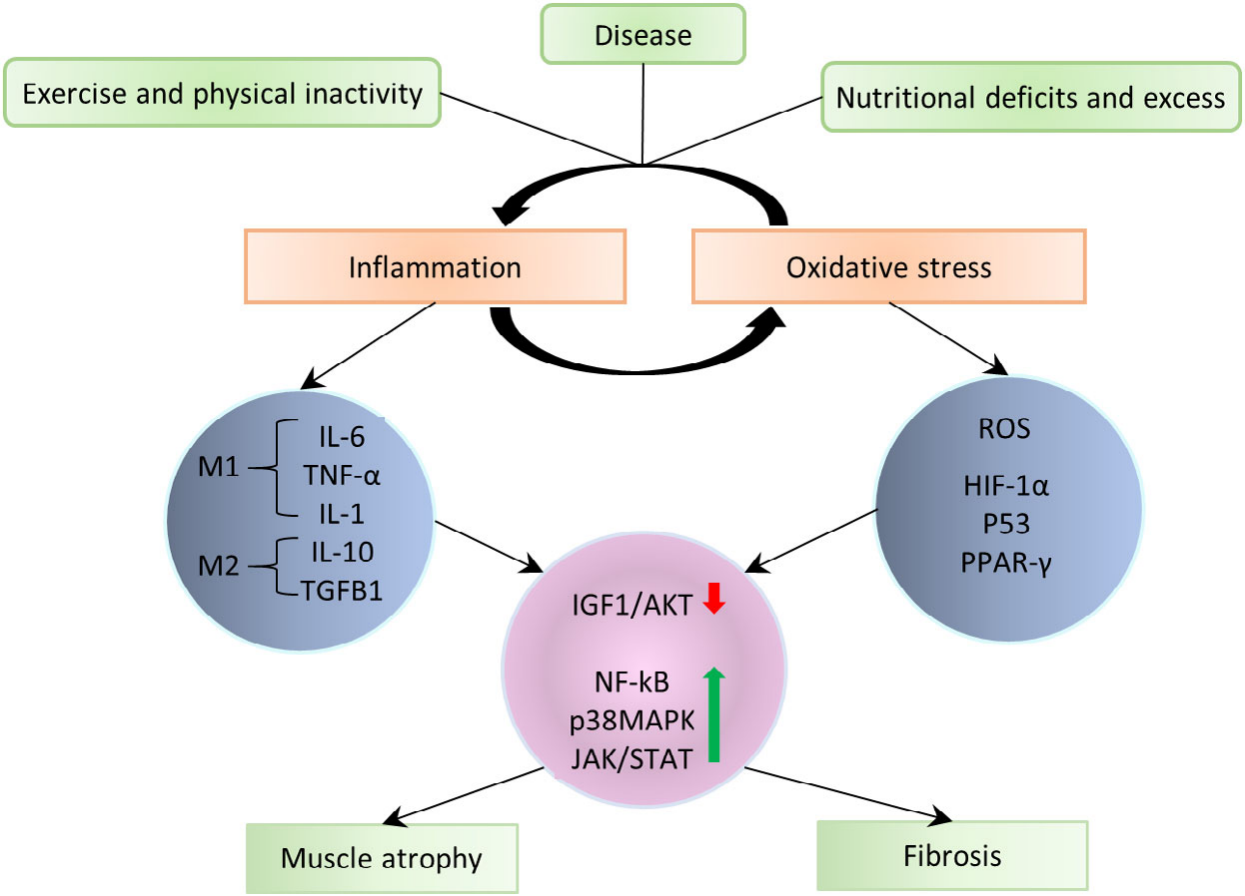
A schematic representation of the relationship between inflammation and oxidative stress—various triggers including excessive exercise or a complete lack, nutritional excess and deficits, or disease and injury greatly influences the mediators

Research in the past two decades has focused extensively on a synergistic relationship between oxidative stress and inflammation, which in turn mediate several chronic diseases of muscular, neurological, nephrological and pulmonary etiology (Biswas, 2016; Cachofeiro et al., 2008; Hald & Lotharius, 2005), in addition to cancer (Khansari, Shakiba, & Mahmoudi, 2009; Reuter, Gupta, Chaturvedi, & Aggarwal, 2010). While oxidative stress is defined as an imbalance between production and removal of free radicals and reactive metabolites (reactive oxygen species, ROS) generated as by‐products of metabolism and catabolism, via protective mechanisms (antioxidants); inflammation is a biological response to injury with the recruitment and activation of several anti‐ and pro‐inflammatory factors (Figure 13). In the following sections, we uncover the most basic mechanisms and response to inflammation and oxidative stress within skeletal muscle.

### 10.1 Inflammation

Inflammatory response is a crucial biological response, which has been extensively studied in the context of skeletal muscle growth and repair, sarcopenia, and myopathies. Inflammation begins with a coordinated activation of several signaling pathways and the recruitment of pro‐/anti‐inflammatory factors such as macrophages and neutrophils to the damage site, initiating tissue repair. Neutrophils represent the most abundant immune cells recruited to the site of injury (within the first 24 hr) with numbers declining past 24 hr, with increasing recruitment of macrophages by 48 hr after injury (Tidball, 2011). However, the precise mechanism by which inflammatory cells are attracted to injury sites is still an active area of research. Recruited and resident immune cells of injured muscle secrete pro‐inflammatory cytokines such as IL‐1, IL‐8, IL‐6, and TNFα triggering a cascade of downstream inflammatory signaling pathways NFκB represents one of the most significant signaling molecule activated upon injury in skeletal muscle (Mourkioti & Rosenthal, 2008). It has long been identified to play a crucial role in atrophying and diseased muscle (e.g., inflammatory myopathies and dystrophinopathies; Jackman et al., 2013; H. Li, Malhotra, & Kumar, 2008).

Canonically, NFκB is triggered by stimulation of pro‐inflammatory factors such as the TNFα or its associated cytokines (Lawrence, 2009), toll‐like receptor family (TLR) and cytokine IL‐1. This triggers the activation of IKKβ leading to phosphorylation and degradation of IκB complexes. NFκB (NFκB1) liberated from IκB inhibitory proteins translocates to the nucleus leading to target gene transcription. Noncanonically, NFκB (p52/NFκB2) is activated by stabilizing NIK (NFκB inducing kinase) through the degradation of TRAF inhibitory proteins. NIK activates IKKα leading to the phosphorylation of p100 and subsequent target gene transcription via p52/RELB (Sun, 2011). NFκB target genes encode cytokines, chemokines, cell adhesion molecules, growth factors, and several enzymes associated with the ubiquitin‐proteasome system. For example, NFκB directly regulates cellular growth, differentiation and metabolism by regulating genes such as cyclin‐D1 (Guttridge, Albanese, Reuther, Pestell, & Baldwin, 1999) and MyoD (Guttridge, Mayo, Madrid, Wang, & Baldwin, 2000; Shintaku et al., 2016). NFκB increases protein turnover via MURF1 (C.‐L. Wu, Cornwell, Jackman, & Kandarian, 2014) and induces IL‐6 activation (Yeagley & Lang, 2010). Cytokines IL‐1 and TNFα are also shown to increase circulating levels of interferon‐γ, which initiates a cascade of events to clear myofiber debris, regulate regeneration (via activation of the JAK1/2‐STAT1 pathway; Doles & Olwin, 2014; Horvath, 2004) and control myogenesis (via CIITA repression of myogenin; Londhe & Davie, 2011). A second wave of macrophages secretes anti‐inflammatory cytokines such as IL‐10 and TGFB1 leading to an ablation in the inflammatory response (L. Arnold et al., 2007). More recently, the anti‐inflammatory action of IL‐10 has been shown to be mediated by a metabolic reprogramming of macrophages where IL‐10 inhibits lipopolysaccharide (LPS)‐induced glycolysis and promotes oxidative phosphorylation. IL‐10 also suppresses mTOR activity causing mitophagy and suppressed inflammasome activation (Ip, Hoshi, Shouval, Snapper, & Medzhitov, 2017). Under certain pathological conditions chronic activation of certain cytokines such as IL‐6 lead to deleterious effects (Muñoz‐Cánoves, Scheele, Pedersen, & Serrano, 2013). IL‐6 is suggested to affect insulin growth factor signaling (IGF1) and shift the balance of STAT3 via SOCS3 protein phosphorylation in favor of a more catabolic profile, promoting muscle atrophy (Haddad et al., 2005).

A balance between the pro‐ and anti‐inflammatory cytokines is essential to maintain muscle health with imbalances leading to deleterious effects, such as incases of chronic inflammation as seen in inflammatory myopathies (IM, polymyositis, dermatomyositis, and inclusion body myositis) (Reimers, Fleckenstein, Witt, Müller‐Felber, & Pongratz, 1993). These IM are clinically characterized by proximal and symmetric muscle weakness and histologically by an excess of inflammatory infiltrates. Histopathology shows evidence for necrosis, fiber size variation, and a muscle degeneration/regeneration phenotype. IMs mostly also exhibit fibrosis (Ueha, Shand, & Matsushima, 2012; see Section 7.1).

### 10.2 Oxidative stress

The high metabolic capacity/activity of skeletal muscle makes it susceptible to increased oxidative stress, with the cell generating ROS including peroxidases, superoxides, and hydroxyl radicals, as a byproduct of normal cellular metabolism (Powers, Ji, Kavazis, & Jackson, 2011). Lack of ROS homeostasis leads to cellular damage and dysfunction via its interaction with/modification of cellular macromolecules (e.g., membrane lipids, DNA, proteins and protein thiol side chains) (Berlett & Stadtman, 1997; Meng & Yu, 2010).

In normal physiology, antioxidant mechanisms maintain free radical homeostasis including release of enzymes such as superoxide dismutase, catalase, and glutathione peroxidase that scavenge ROS (Kozakowska, Pietraszek‐Gremplewicz, Jozkowicz, & Dulak, 2015). Oxidative stress regulates/is regulated by a host of transcription factors including NFκB (Morgan & Liu, 2011), p53 (Beyfuss & Hood, 2018), HIF‐1α (Mason & Johnson, 2007), leading to the expression of several GFs, inflammatory cytokines, chemokines, and cell cycle regulatory molecules. Studies have associated increasing ROS with fiber atrophy and necrosis observed in cases of severe muscle disuse (Powers, Smuder, & Judge, 2012), sarcopenia (Brioche & Lemoine‐Morel, 2016), obesity and diabetes (Haskins, Bradley, Powers, et al., 2003; Newsholme, Cruzat, Keane, Carlessi, & de Bittencourt, 2016), and muscular dystrophies (M. H. Choi, Ow, Yang, & Taneja, 2016; Terrill et al., 2013).

Oxidative stress inhibits the AKT/mTOR pathway and its downstream targets, subsequently suppressing protein synthesis and promoting atrophy (O'Loghlen, Perez‐Morgado, Salinas, & Martin, 2006; Tan, Shavlakadze, Grounds, & Arthur, 2015; L. Zhang, Kimball, Jefferson, & Shenberger, 2009). Alternatively, AMPK activation in response to oxidative stress also inhibits protein synthesis via mTOR1 and TSC2 phosphorylation (Y. Zhao et al., 2017). An autophagic response leading to atrophy can also be initiated via mTOR phosphorylation. mTOR1 activation induces the autophagosome formation by activating a required protein, the ULK1 complex (ULK1, ATG13, and FIP200), and subsequently the PI3K complex (in the presence of Ambra1) (Di Rienzo, Antonioli, Fusco, et al., 2019; Nazio & Cecconi, 2013). Superoxide or hydroxyl radicals derived from superoxides are suggested to contribute to the oxidative damage especially during reperfusion in muscle (Zweier & Talukder, 2006). In addition to metabolism (mitochondrial ROS production), a chronic inflammatory response can activate nicotinamide adenine dinucleotide phosphate oxidases and other inducible family of enzymes, which produce ROS, cyclically amplifying ROS and triggering further inflammation in skeletal muscle. A detailed review on the relationship between oxidative stress and autophagy is presented in Rodney, Pal, and Abo‐Zahrah (2016).

## 11 ENERGY METABOLISM

**Figure 14:**
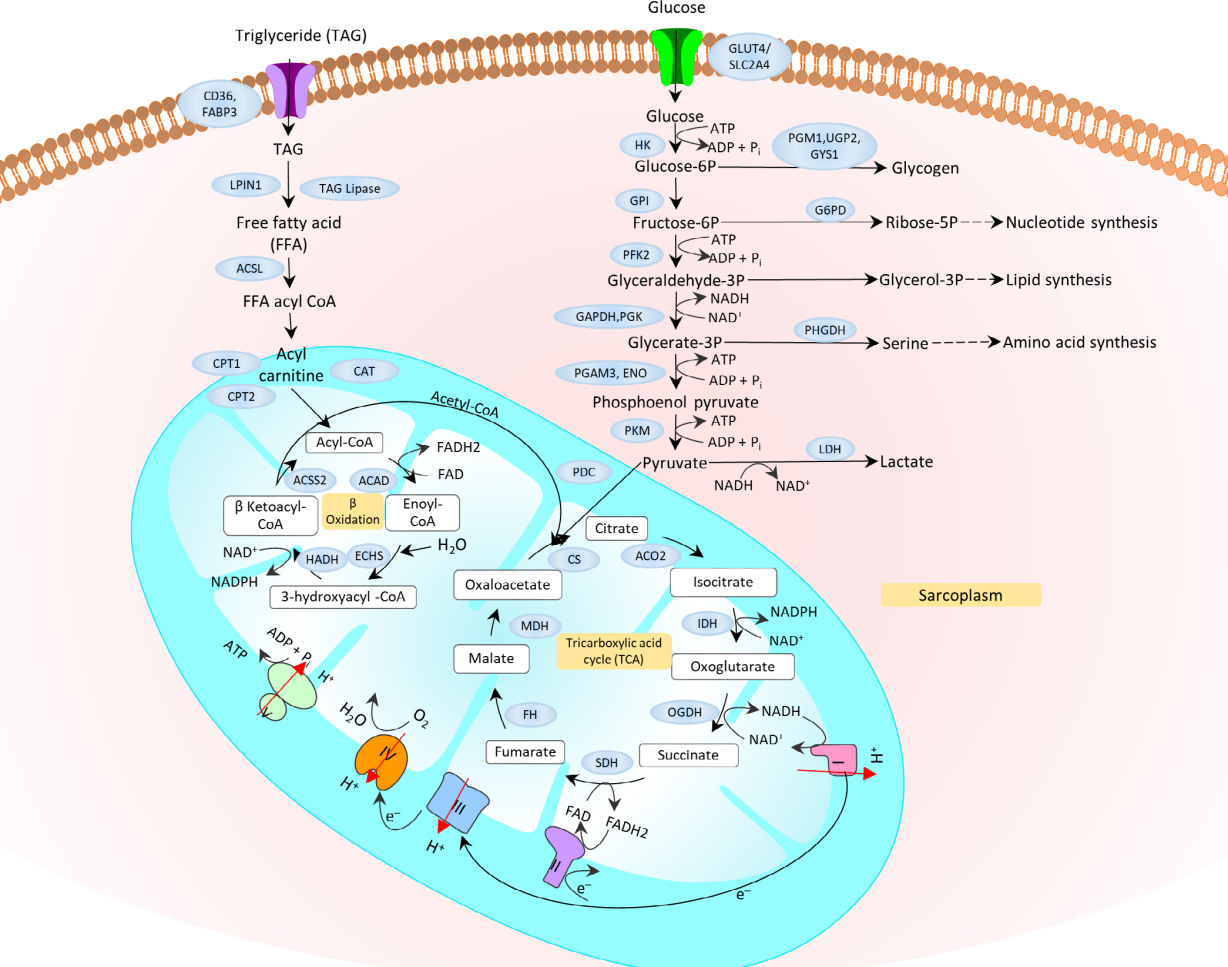
Schematic representation of major molecular markers involved in muscle energy metabolism—glycolysis occurs outside the mitochondrion—when a six‐carbon is converted to a 3‐carbon pyruvate molecule generating energy in the form of ATP and NADH. Pyruvate is additionally imported into the mitochondrion, where it is converted to acetyl‐CoA and enters the citric acid (TCA) cycle. Acetyl‐CoA is also generated via β‐oxidation of lipids in the mitochondria. The energy produced during TCA (NADH) is utilized by the electron transport chain, in the cristae of the mitochondria to generate three energy rich ATP molecules and water. In anaerobic glycolysis, the NADH produced is utilized for lactate production, in contrast to the OXPHOS system for ATP generation under aerobic conditions. For simplicity, the number of molecules of ATP, NADH, or NAD are not shown

In addition to liver, skeletal, and cardiac muscles represent major sites for maintaining an organism's energy homeostasis. The high metabolic capacity of skeletal muscle is driven by an extensive requirement of ATP by the cross‐bridge cycle (see Section 6.2), required to generate force and motion. Two processes serve as the major mechanisms for muscle energy metabolism, namely, glycolysis and oxidative metabolism and are discussed in the following sections (Figure 14).

### 11.1 Glycolytic metabolism/glycolysis

Rapid ATP requirements are catered to by metabolism of glucose either aerobically or anaerobically in the exercising muscle. Glycolytic metabolism serves as the primary source of energy, especially, in fast Type II fibers during short intense activity bursts, in a setting of limited blood flow and oxygen (hypoxia). Anaerobic glycolysis occurs in conditions of high‐intensity and sustained isometric activity (such as lifting weights; Spriet, 1992) with muscle shifting to an aerobic glycolysis profile during isotonic exercise (such as walking). It is well known that most cancers adopt high rates of glycolysis irrespective of oxygen abundance (Warburg effect; Vander Heiden, Cantley, & Thompson, 2009).

Glycolysis begins with the transport of glucose across the sarcolemma by GLUT4 (Leto & Saltiel, 2012). GLUT4 is an insulin‐sensitive glucose transporter, crucial for glucose uptake by skeletal muscle and is promoted by AMPK activation (Musi & Goodyear, 2003). AMP‐AMPK, a serine/threonine kinase, is a key modulator of skeletal muscle metabolism that controls both transcription and phosphorylation states of metabolic enzymes (Hardie, 2011; Jäger, Handschin, Pierre, & Spiegelman, 2007). AMPK, which also serves as a nutrient sensor, is activated in the muscle during reduced ATP levels, in response to intense exercise or cellular stresses (e.g., oxidative stress). Chronic AMPK activation alters metabolic gene expression and induces mitochondrial biogenesis (Bergeron et al., 2001; McGee et al., 2003).Glucose is prepared for glycolysis by the phosphorylating enzyme hexokinase (HK1). Phosphorylase (PYGM) and other debranching enzymes produce αD‐Glucose 1P from glycogen. GYS1 serves to replenish intracellular glucose from glycogen stores. The rate‐limiting step in glycolysis, however, is the conversion of fructose‐6‐phosphate to fructose‐1,6‐diphosphate by the enzyme phosphofructokinase (PFKM). The last step in anaerobic glycolysis is the conversion of pyruvate to lactate by lactate dehydrogenase (LDHA/LDHB) (and the NADH is not utilized by OXPHOS), while the pyruvate is converted to acetyl CoA via pyruvate dehydrogenase complex (PDHA, DLAT, and DLD) during aerobic glycolysis, and is utilized by the tricarboxylic acid (TCA) cycle to generate ATP in the mitochondrial matrix (see oxidative metabolism). Additional molecular markers involved in glycolysis are identified in Figure 14.

Disruption in glycogen storage and metabolism result in glycogen storage diseases of the muscle (Hers, 1964; Özen, 2007). These diseases are often associated with exercise intolerance arising from limited energy supply and excessive glycogen buildup. Though accumulation of inorganic phosphates (Pi) and ADP are known to contribute to muscle fatigue, the major player in glycogen storage diseases appear to be acidification resulting from increasing lactate concentrations (lactic acidosis) within the muscle fibers. Immediate energy requirements in the muscle are additionally met by muscle creatine kinase (CKM) which transfers high‐energy phosphates (Pi) from phosphocreatine stores, to convert the ubiquitous ADP to ATP (Baird, Graham, Baker, & Bickerstaff, 2012).

### 11.2 Oxidative metabolism

Oxidative metabolism serves as the primary source of energy production and utilizes both lipids and the products of glycolysis for satisfying sustained energy requirement through aerobic oxidation, linking it tightly to the physical activity of an organism. Breakdown of lipids in muscle occurs mainly through β‐oxidation of fatty acids (Eaton, Bartlett, & Pourfarzam, 1996). β‐Oxidation begins with lipids/triglycerides broken down to free fatty acids via lipin and TAG lipases. Fatty acids primarily enter the muscle cell through fatty acid transporters such as fatty acid translocases (e.g., CD36), SLC27 family of fatty acid transporters (FATP/SLC27A1) and plasma membrane bound fatty acid binding protein (e.g., FABP3). Once inside the cell, a CoA group is added to the fatty acid by fatty acyl‐CoA synthase, forming long‐chain acyl‐CoA (Berg, Tymoczko, & Stryer, 2002).

Mitochondrial content or volume within muscle is a major quantitative indicator of the muscle's oxidative capacity. Mitochondria consume the greatest amount (some 85–90%) of oxygen in cells to allow mitochondrial oxidative phosphorylation (OXPHOS), which is the primary metabolic pathway for ATP production (Gnaiger, 2009). The coupling of upstream oxidative processes (glycolysis, β‐oxidation, and TCA turnover) to OXPHOS during energy demand results in release of free energy as ATP (Kunz, 2001). As the first step, carnitine palmitoyltransferase 1 (CPT1) converts long‐chain acyl‐CoA to long‐chain acylcarnitine allowing fatty acid moieties to be transported across the inner mitochondrial membrane via carnitine translocase, which exchanges long‐chain acylcarnitines for carnitine. The inner mitochondrial membrane CPT2 converts long‐chain acylcarnitine back to long‐chain acyl‐CoA. The long‐chain acyl‐CoA enters the fatty acid β‐oxidation pathway, resulting in the production of acetyl‐CoA, which enters the mitochondrial TCA cycle (Berg et al., 2002; Wanders, Ruiter, IJlst, Waterham, & Houten, 2010). The NADH and FADH2 produced by both fatty acid β‐oxidation and TCA cycle are used by the electron transport chain to produce three energy‐rich ATP molecules and a water molecule, in the cristae of the mitochondria (OXPHOS) (Kunz, 2001). More recently, cellular localization of the mitochondrial OXPHOS system has been detected in the sarcolemma (H. Lee et al., 2016). Details of several molecular actors in fatty acid oxidation are provided in Figure 14.

The peroxisome proliferator‐activated receptor γ (PPARγ) and its coactivator PPARγ 1α (PGC‐1α) tightly regulate oxidative metabolism and drive the expression of several genes responsible for ATP synthesis. PGC‐1α binds to, and increases the activity of PPARs, which regulate several genes including FATP, ACS, CD36, MCAD, CPT1, and LCAD (Muoio & Koves, 2007). Increased activation of PGC‐1α is associated with increased mitochondrial biogenesis in the muscle (Z. Wu et al., 1999). PGC‐1α is controlled by AMPK, which functions to either directly affect PGC‐1α phosphorylation or activate SIRT‐1, a deacetylase which increases the activity of PGC‐1α (Cantó & Auwerx, 2009). The oxidative phenotype and the activation of PGC‐1α is linked to physical activity levels and beneficial effects in metabolic diseases and other pathologies (H. Liang & Ward, 2006). In addition to glycolysis and β‐oxidation, amino acids can supply substrates to the TCA cycle for sustained mitochondrial ATP production; for example, the amino acid, glutamine, can generate glutamate, which subsequently fuels the TCA cycle through a series of biochemical reactions termed glutaminolysis (DeBerardinis, Mancuso, Daikhin, et al., 2007).

### 11.3 Effect of exercise on metabolism

Exercise intensity (aerobic or endurance training vs. anaerobic or resistance training) determines the choice of either a glycolytic or an oxidative metabolic profile (Baker, McCormick, & Robergs, 2010) in fiber types. The relative contribution of carbohydrate and lipid to oxidative metabolism during exercise is further influenced by prior diet, training status, sex, and environmental conditions (Jeukendrup, 2004; Romijn, Coyle, Sidossis, et al., 1993) which in turn affect the availability of several important factors such as ATP, levels of circulating hormones (e.g., insulin), substrates, and metabolites. For instance, as mentioned earlier, PGC‐1α has been identified as a core regulator of mitochondrial biogenesis. A single bout of endurance exercise is shown to induce rapid and sustained increase of PGC‐1α (Mathai, Bonen, Benton, Robinson, & Graham, 2008) with improvements to whole‐body oxygenation (peak oxygen uptake), and a shift from carbohydrate to fat substrates (Calvo et al., 2008). Overexpression studies of PGC‐1α have also shown large increases in functional mitochondrial and genetic programs characteristic of slow‐twitch fibers resistant to contraction‐induced fatigue (Lin et al., 2002). Knockout of PGC‐1α in mice models was shown to cause a shift in fiber types from oxidative Type I and IIA to Types IIX and IIB. These animals also exhibited reduced endurance capacity and increased fiber damage, further emphasizing the role of PC‐1α in maintaining muscle fiber integrity and energy homeostasis (Handschin et al., 2007).

Utilization of carbohydrate substrates increase with increasing exercise intensity, coupled with reduced lipid oxidation. It is suggested that carnitine, which is essential for CPT1 regulation, serves as the regulatory candidate for fatty acid oxidation in muscle. As exercise intensity increases, the level of free carnitine fall reducing CPT1 activity and inhibiting β‐oxidation (Jeppesen & Kiens, 2012). Exercise training, its intensity, duration, and glucose supply, have been shown to be factors regulating GLUT4 (regulated by AMPK) translocation and activity. Regulation of GLUT4 contributes to improved insulin action, glucose disposal and enhanced muscle glycogen storage following exercise (reviewed in Richter & Hargreaves, 2013). The interaction between fat and carbohydrate metabolism in exercise is further reviewed in Spriet (2014).

### 11.4 Immunometabolism—A synergistic relationship between immunity and metabolism

Skeletal muscle serves as the major site for insulin‐stimulated glucose disposal and subsequently, glucose homeostasis. Association of metabolic and cardiovascular diseases with exercise and muscle metabolism are widely acknowledged. Several of these diseases also exhibit chronic tissue inflammation with obesity, as an underlying etiology. Recent research has begun to unravel the complexity of this cross‐talk (both inter‐ and intra‐organ) between inflammation and metabolism, spawning a body of active and rapidly expanding research called “immunometabolism” (Hotamisligil, 2017a). In the following section, we highlight several important factors that have been identified as contributing to immunometabolism within skeletal muscle. We do not focus on a whole‐body view (intra‐organ) signaling that drives communication between immune and metabolic factors (Hamrick, 2011, 2012; Y. S. Lee, Wollam, & Olefsky, 2018).

**Figure 15:**
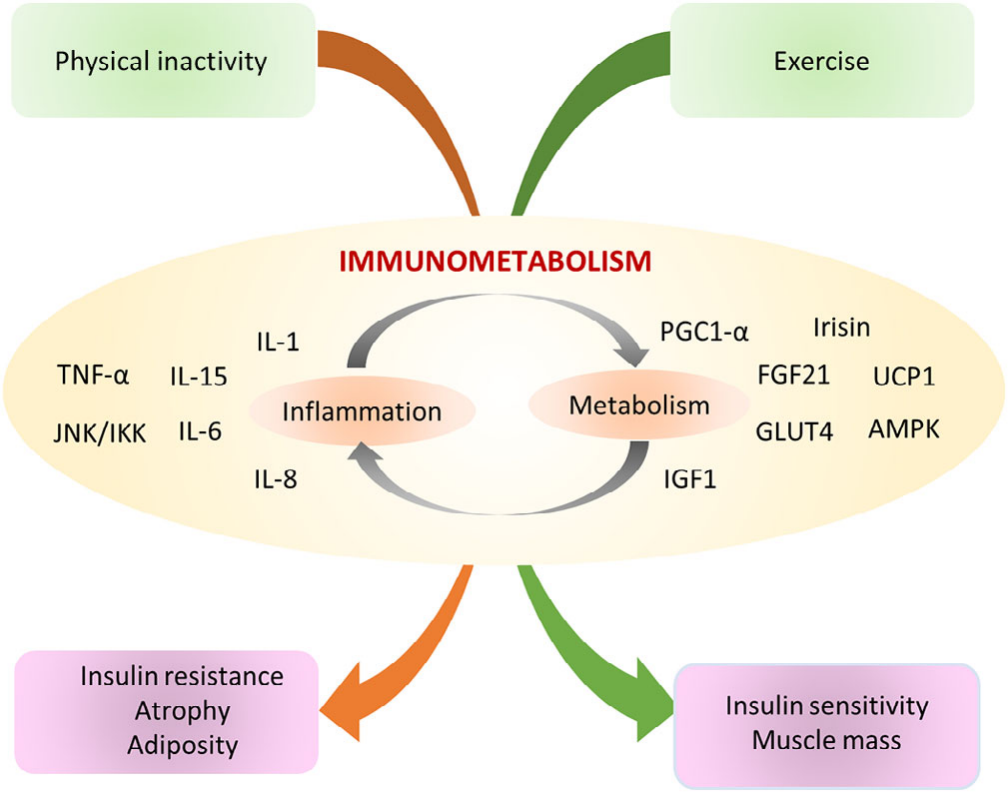
Schematic representation of the key signaling molecules in immunometabolism. The cross‐talk between metabolic and inflammatory signaling pathways occurs at multiple levels (inter‐ and intra‐tissue/organ). Several of the above shown factors are part of the skeletal muscle secretome (myokines; adipomyokines) such as IL‐15 a regulator of muscle adiposity, IL‐6 crucial for inflammation and glucose homeostasis; and several fat/ glucose metabolic genes (AMPK, FGF21, IGF1,PGC1α, Irisin) that play a pivotal role in maintaining the immunometabolic profile of skeletal muscle

Skeletal muscle, in states such as exercise, injury, inactivity or disease, is replete with infiltrating immune cells (e.g., macrophages, Pillon & Krook, 2017), and circulating immune factors (cytokines and adipokines derived from muscle fat depots—intermyocellular and perimuscular adipose tissue; Khan et al., 2015). Additionally, it is also now established that the skeletal muscle is an endocrine organ secreting cytokines and other peptides (such as IL‐6, IL‐8, IL‐15, IGF1, FGF21, FSTL1, irisin, all termed “myokines”), whose levels are regulated by muscle contractile activity (Febbraio & Pedersen, 2005; Pedersen, 2011; Schnyder & Handschin, 2015) and subsequently exercise (So, Kim, Kim, & Song, 2014, p. 201). Interestingly, several of these are known to be secreted by adipocytes and are often referred to in literature as adipomyokines (Raschke & Eckel, 2013). For instance, irisin, a recently discovered and much debated exercise (PGC‐1α) induced myokine (Albrecht et al., 2015; Panati, Suneetha, & Narala, 2016) is suggested to be a metabolic regulator in muscle (Blizzard LeBlanc, Rioux, Pelech, et al., 2017; Perakakis et al., 2017). A detailed review on role of exercise in influencing the action of several myokines mentioned here, and the cross‐talk between muscle and adipose tissues are presented in Stanford and Goodyear (2018). These cytokines/myokines exert auto‐, para‐ and/or endocrine effects in a context‐specific manner enabling muscle to maintain the metabolic homeostasis of lipids and proteins, in health and exercise (C. Brandt & Pedersen, 2010; Pedersen, Akerstrom, Nielsen, & Fischer, 2007; Figure 15).

A shift in balance from an immunometabolic adaptive profile, observed in healthy muscle tissue, to a maladaptive state as observed in chronic metabolic disorders (obesity, Ray, Mahata, & De, 2016 and T2DM), occurs through deficient cross‐talk between immune and metabolic signaling factors such as the inflammasome (Próchnicki & Latz, 2017), insulin receptors (Hotamisligil, 2017b), TNFα (Austin, Rune, Bouzakri, Zierath, & Krook, 2008; Hotamisligil, Shargill, & Spiegelman, 1993), and other cytokines (Fink, Oberbach, Costford, et al., 2013; Pillon & Krook, 2017).

For instance, elevation in circulating TNFα levels (observed in tissues such as adipose (Hotamisligil et al., 1993) and muscle (Saghizadeh, Ong, Garvey, Henry, & Kern, 1996)) causes a downstream activation of stress kinases, triggering TNF‐mediated insulin resistance and glucose dyshomeostasis (reviewed in Hotamisligil, 2017b).

IL‐6 is shown to have acute insulin‐like effects (Carey et al., 2006; Ellingsgaard et al., 2011; Pedersen & Febbraio, 2008) in healthy muscle, which do not persist under chronic conditions. IL‐6 enhances glucose uptake and translocation of the glucose transporter GLUT4, enhancing insulin‐stimulated glucose uptake (Carey et al., 2006) while chronically elevated IL‐6 has been shown impair insulin‐stimulated glucose uptake in muscles (Franckhauser, Elias, Sopasakis, et al., 2008). Circulating IL‐6 is also known to exert anti‐inflammatory effects in the context of exercise. Elevation of IL‐6 during exercise induces an anti‐inflammatory environment by inducing the production of IL‐1ra and IL‐10, and also inhibiting TNF‐α production; subsequently abating the chronic systemic low‐grade inflammation seen in cardiovascular disease, Type 2 diabeters, and muscle wasting (Pedersen & Febbraio, 2008). IL‐15 a circulating, exercise‐induced myokine has been shown to inhibit adipose tissue deposition (Quinn & Anderson, 2011; Quinn, Anderson, Strait‐Bodey, Stroud, & Argilés, 2009) and influence accumulation of fat and regulation of adiposity in muscle during inactivity (Pedersen & Febbraio, 2012). Other mechanisms that contribute to lipid‐induced modulation of insulin resistance (in vitro) have also been identified, such as stimulation of TLR4 (a classical innate immune surface receptor) which switches muscle metabolism to glycolysis (via LPS), inducing insulin resistance (H. Liang, Hussey, Sanchez‐Avila, Tantiwong, & Musi, 2013).

Metabolism within the factors associated with immunity, also affects their expression and function. For instance, metabolite profiling of activated macrophages has shown that accumulation of Kreb's cycle intermediates which are important for production of inflammatory cytokines (Jha et al., 2015). Macrophage polarization been shown to be affected by nutrient sensing pathways such as AMPK and mTOR1. Macrophages lacking a catalytic AMPK subunit (AMPKa1) are shown to have defective M2 polarization (Mounier et al., 2009). Constitutive mTOR1 activation has also been shown to result in defective M2 polarization, resulting in an enhanced pro‐inflammatory response to LPS (Byles et al., 2013). These mechanisms highlight the complex and yet to be fully understood interactions between immune and metabolic factors driving muscle heath.

## 12 SKELETAL MUSCLE CIRCULATION

Skeletal muscle accounts for ~40% of the total body weight while accounting for ~25% of the cardiac output to meet basal metabolic needs. The interested reader is directed to Korthuis (2011), for a detailed overview of skeletal muscle circulation. Briefly, anatomically, skeletal muscle is oxygenated and deoxygenated by an elaborate network of arteries and veins, respectively, whose density varies between muscle types. The arteries divide further into a network of smaller arteries (called arterioles) which penetrate the perimysium, arranged perpendicular to the muscle fiber axis branching terminally into a fine mesh of capillaries. This network joins with the network of venules and veins, giving rise to a rich lattice of vasculature enmeshing bundles of muscle fibers. Vascular smooth muscle cells (VSMCs), endothelial cells (ECs), and pericytes represent major cell types of the vascular walls. As the energy requirements vary across muscle types, so does the density of vasculature and thickness of capillaries to cater to its oxygen demand. Blood flow is also largely regulated by alterations in vascular resistance and blood viscosity. Vascular resistance (vasoconstriction) depends on the contraction of VSMCs, triggered by the availability of cytoplasmic free Ca^2+^. Free Ca^2+^ triggers formation of the Ca^2+^‐calmodulin complex, which in turn activate myosin light chain kinase, a CaMK, that phosphorylates myosin light chains, bringing about smooth muscle contraction. Ca^2+^ sensitization of the contractile proteins is signaled by the RhoA/Rho kinase pathway to inhibit the dephosphorylation of the light chain by myosin phosphatase (MLCP), maintaining force generation. Removal of Ca^2+^ from the cytosol and stimulation of MLCP initiates smooth muscle relaxation (Webb, 2003).

A host of molecular factors have been identified to modulate free Ca^2+^ concentration: calcium‐gated and permeable channels (Ghosh et al., 2017) such as CACN1C (Cav1.2), CACNA1G (Cav3.1), CACNA1H (Cav3.2), CACNA1L (Cav3.3); potassium channels (Jackson, 2005, 2018) such as KCNA5 (KV1.5), KCNA6 (KV1.6), KCNJ8 (KIR6.1), KCNJ2 (KIR2.1), KCNMA1 (Slo1); and EC‐derived factors (Kedzierski & Yanagisawa, 2001) such as endothelium‐derived hyperpolarizing factor, endothelin (a vasoconstrictor), and vasodilators such as NO, CO, and H_2_S.

Skeletal muscle research has focused extensively on the role of nitric oxide (NO) in vasodilation (McConell, Rattigan, Lee‐Young, Wadley, & Merry, 2012) and in response to exercise and injury (Hong, Betik, & McConell, 2014; McConell et al., 2012; Stamler & Meissner, 2001). NOS/cyclic guanosine monophosphate (cGMP)‐induced relaxation is shown to correlate with MLCP phosphorylation (Francis, Busch, & Corbin, 2010; Nakamura, Koga, Sakai, Homma, & Ikebe, 2007). Neuronal and endothelial nitric oxide synthase (nNOS/NOS2 and eNOS/NOS3) represent major muscle‐specific synthases that are activated by the interaction between Ca^2+^ and calmodulin. NO diffuses into VSMCs to activate guanylyl cyclase which results in the production of cGMP (Kobzik, Reid, Bredt, & Stamler, 1994) and a subsequent activation of protein kinase G, resulting in vasodilation. With dynamic exercise, there is considerable remodeling of the vascular system (Green, Spence, Rowley, Thijssen, & Naylor, 2012) driven by NO and VEGF (Hoier & Hellsten, 2014). VEGF represents an important class of angiogenic factors that affect and influence skeletal muscle circulation as detailed below.

### 12.1 Vascular endothelial growth factor signaling

Vascular endothelial growth factor A (VEGFA), with nine known isoforms, is the major regulator of vasculature development during embryogenesis (vasculogenesis) (Ferrara et al., 1996) and a potent inducer of neovascularization in adult tissue (angiogenesis) (Patan, 2004). VEGFA stimulates angiogenesis by promoting EC migration, proliferation and differentiation to form new vessel structures. VEGF induces DLL4, which functions to pattern the endothelial population into tip and stalk cells. VEGFA also serves as an angiogenic stimulus guiding tip cells through the ECM. Tip cells, enriched with VEGFA receptors (VEGFR2) sense and align spatially along the VEGFA gradient, thus providing a map for alignment of proliferating stalk cells to form capillaries (Gerhardt, 2008). Sufficient oxygen perfusion into the muscle upon capillary formation and maturation normalizes VEGFA levels. VEGF driven angiogenesis is heavily regulated by the expression of two of its receptors—VEGFR1 and VEGFR2 (Olsson, Dimberg, Kreuger, & Claesson‐Welsh, 2006).

VEGFR2 mediates most of the endothelial growth and survival signals and contributes to re‐organization of the cytoskeleton by phosphorylating FAK (focal adhesion kinase) and paxillin, while VEGFR1, an early inhibitor of angiogenesis plays an important role in disease, progression and management (Amano, Kato, Ito, et al., 2015; Hiratsuka et al., 2001; Jain, 2005). Interaction between VEGFR1 and VEGFR2, regulated by placental growth factor has been identified and suggested to amplify VEGFA‐driven angiogenesis (Autiero, Waltenberger, Communi, et al., 2003; Autiero, Luttun, Tjwa, & Carmeliet, 2003). Recent research suggests that VEGFR1 predominantly modulates VEGF activity and subsequently EC homeostasis by forming heterodimers with VEGFR2 (Cudmore et al., 2012). In addition to ECs, SCs and differentiating myoblasts also generate VEGFA in the muscle (R. S. Williams & Annex, 2004). Regenerating muscle is characterized by increased capillarization. It has been suggested that the increased expression of VEGFA and its receptors, in regenerating muscle promotes growth and fusion of myofibers and SC activation leading to a more rapid regeneration enabled via several mechanisms including activation of MAPK, PI3K/AKT pathways and SC activation (Arsic et al., 2004).

A bidirectional, reciprocal relationship between ECs and SCs is suggested to exist within the stem cell niche. In co‐culture experiments, ECs were found to promote myoblast proliferation by secreting a panel of GFs, such as IGF‐I, HGF, FGF, PDGF, and VEGF (Christov et al., 2007). Contrastingly, VEGFA was shown to promote re‐entry of SCs into quiescence. SCs in the proximity of pericytes and capillaries allow for angiopoietin‐1 binding on their TIE2 receptors, simultaneously stabilizing vessels and promoting SC quiescence through the ERK1/2 pathway. Based on these observations, it has been proposed that during muscle regeneration, ECs and SCs interact with each other promoting myo‐angiogenesis (Abou‐Khalil, Mounier, & Chazaud, 2010; Mounier, Chrétien, & Chazaud, 2011).

## 13 COMMON MOLECULAR MECHANISMS UNDERLYING MUSCLE DISEASES

**Figure 16:**
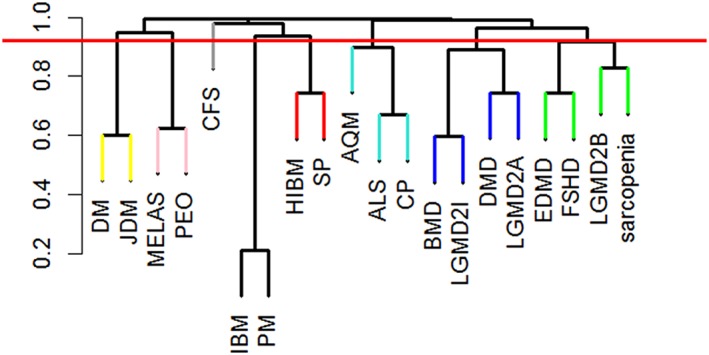
Extracting significant disease similarities from 20 diseases affecting muscle—above figure shows the hierarchical clustering dendrogram of disease correlation. Colors on the tree indicate the clusters/grouping of diseases, while the red line indicates the threshold used for clustering. (Reprinted with permission from Mukund and Subramaniam (2017). Copyright 2017 Frontiers Publication)

We have integrated in each section on muscle function, specific diseases that arise from genetic mutations and from aberrant functional pathways, including their clinical characteristics where appropriate (italicized); this is catalogued in Supplementary Table 2. In addition, we recently explored common and unique aspects of muscle disorders using transcriptional profiles and a systems biology approach (Mukund & Subramaniam, 2017; Figure 16). Our analysis revealed that a majority of muscle diseases share a few common mechanisms. Across the 20 muscle diseases in our study, we identified deficient bioenergetics and a lack of Ca^2+^ homeostasis as aberrant mechanistic signatures underlying muscle pathophysiology. Recent research in muscular degeneration (muscular dystrophies, Ramadasan‐Nair et al., 2014; cardiomyopathies, Wallace, 2000; and neuromuscular diseases such as ALS, Cozzolino & Carrì, 2012; Dupuis & Loeffler, 2009) have all identified mitochondrial dysfunction as a cause underlying the disease. Bioenergetics pathway enzymes have also recently been shown to be relevant biomarkers for muscular disease progression (Santacatterina et al., 2015).

Reduced efficiency in the action of the TCA cycle has been also assessed in diseased muscle associated with inflammatory myopathies (Coley et al., 2012), and muscular dystrophy (Even, Decrouy, & Chinet, 1994). Reduced ATP availability, contributing to suppressed muscle regeneration and an altered Ca^2+^ homeostasis are suggested to be pivotal to muscle wasting observed in certain dystrophies such as DMD‐d (Timpani, Hayes, & Rybalka, 2015). Ca^2+^ homeostasis in muscle determines its integrity and function, regulated mainly by SERCA pumps and RYRs. Work from our laboratory has previously identified strong dysregulation of these proteins in ALS and DMD‐d (Mukund & Subramaniam, 2015; Y. Wang, Winters, & Subramaniam, 2012). Likewise, regulation of genes regulating the action of these proteins such as ASPH (regulator of RYRs) and SLN have also been observed in muscle from cerebral palsy, a neuromuscular disease (Smith, Lee, Ward, Chambers, & Lieber, 2011).

Several of these debilitating muscle diseases often exhibit muscle atrophy, hypertrophy and fibrosis, occurring in various combinations and to varying degrees of severity (as discussed in Sections 8.1 and 10). Interestingly, however, muscle, upon injury or insult, begins to express a plethora of mixed fiber and immature muscle isoforms including myosins, actins, and members of the Ca^2+^ homeostasis machinery across diseases (Mukund et al., 2014), particularly dystrophies (reviewed in Beedle, 2016) and neuromuscular diseases such as ALS (Y. Wang et al., 2012). Cardiac muscle research suggests that activation of fetal isoforms in failing heart (cardiac muscle) confers an initial protective effect on heart function. However, precise consequences of present or persistent immature/mixed isoforms expression in skeletal muscle pathophysiology are not yet understood and offer exciting avenues for future research.

## 14 CONCLUSION

Human skeletal muscle is often characterized as a mechanical device responsible for generating contraction, force, and movement. Over the past decades, a detailed molecular picture of the skeletal muscle has begun to emerge, where each molecular player is associated with a “functional component” of the muscle. While some of these functional components, such as the contractile machinery or the sensing apparatus at the NMJ have received significant attention, a detailed mapping of known molecular components and their relationship to muscle function has not yet been broadly reviewed. This review is aimed at highlighting molecular components of the muscle, and the complex molecular cross‐talk with its various interacting partners (e.g., immune infiltrates, fibroblasts, adipocytes, nerve cells, ECs, environmental stressors) influencing and contributing to the health and activity of muscle tissue.

Given the breadth of muscle research, we acknowledge that thoroughly outlining every significant contribution within each of the components described here is a massive undertaking and not currently within the scope of this article. However, the comprehensive systems‐level molecular and functional pathway perspectives provided in this review, attempt to introduce the reader to important mechanisms in muscle that not only pave the way for a deeper analysis of muscle function in health and disease, but provide interesting insights into the molecular machinery that is core to muscle function. The adaptability of skeletal muscle as it attempts to revert into a precursor‐like state in response to insult or injury, as witnessed through the increased expression of fetal gene isoforms warrants further research. The contextual mechanisms described here in, also provide the basis for further investigations on the precision and limitations of pharmacological interventions.

## CONFLICT OF INTEREST

The authors have declared no conflicts of interest for this article.

## RELATED WIREs ARTICLES

https://doi.org/doi: 10.1002/wsbm.1184

https://doi.org/doi: 10.1002/wsbm.1197

## Supporting information


**Supplementary Table 1**. This table presents a list of genes (which have been referred to in the manuscript using their commonly used names) and their corresponding NCBI isoform gene symbol, relevant to our manuscript.
**Supplementary Table 2**. A list of myopathies with known genetic associations in muscle genes mentioned in the manuscript is presented. Inflammatory, idiopathic myopathies or myopathies resulting as a consequence of other diseases are not listed in the table.

## References

[wsbm1462-bib-0001] Wu, K. , Li, A. , Rao, M. , Liu, M. , Dailey, V. , Yang, Y. , … Pestell, R. G. (2006). DACH1 is a cell fate determination factor that inhibits cyclin D1 and breast tumor growth. Molecular and Cellular Biology, 26(19), 7116–7129. 16980615 10.1128/MCB.00268-06 PMC1592900

[wsbm1462-bib-0002] Abou‐Khalil, R. , Mounier, R. , & Chazaud, B. (2010). Regulation of myogenic stem cell behaviour by vessel cells: The "ménage à trois" of satellite cells, periendothelial cells and endothelial cells. Cell Cycle, 9(5), 892–896. 20160472 10.4161/cc.9.5.10851

[wsbm1462-bib-0003] Ackermann, F. , Waites, C. L. , & Garner, C. C. (2015). Presynaptic active zones in invertebrates and vertebrates. EMBO Reports, 16(8), 923–938. 26160654 10.15252/embr.201540434 PMC4552486

[wsbm1462-bib-0004] Ackermann, M. A. , & Kontrogianni‐Konstantopoulos, A. (2013). Myosin binding protein‐C slow: A multifaceted family of proteins with a complex expression profile in fast and slow twitch skeletal muscles. Frontiers in Physiology, 4, 391. 24399972 10.3389/fphys.2013.00391 PMC3872291

[wsbm1462-bib-0005] Ackermann, M. A. , Ziman, A. P. , Strong, J. , Zhang, Y. , Hartford, A. K. , Ward, C. W. , … Bloch, R. J. (2011). Integrity of the network sarcoplasmic reticulum in skeletal muscle requires small ankyrin 1. Journal of Cell Science, 124(21), 3619–3630. 22045734 10.1242/jcs.085159 PMC3215573

[wsbm1462-bib-0006] Alabi, A. A. , & Tsien, R. W. (2013). Perspectives on kiss‐and‐run: Role in exocytosis, endocytosis, and neurotransmission. Annual Review of Physiology, 75, 393–422. 10.1146/annurev-physiol-020911-153305 23245563

[wsbm1462-bib-0007] Albrecht, E. , Norheim, F. , Thiede, B. , Holen, T. , Ohashi, T. , Schering, L. , … Maak, S. (2015). Irisin—A myth rather than an exercise‐inducible myokine. Scientific Reports, 5, 8889. 25749243 10.1038/srep08889 PMC4352853

[wsbm1462-bib-0008] Allen, R. E. , & Boxhorn, L. K. (1989). Regulation of skeletal muscle satellite cell proliferation and differentiation by transforming growth factor‐beta, insulin‐like growth factor I, and fibroblast growth factor. Journal of Cellular Physiology, 138(2), 311–315. 2918032 10.1002/jcp.1041380213

[wsbm1462-bib-0009] Almada, A. E. , & Wagers, A. J. (2016). Molecular circuitry of stem cell fate in skeletal muscle regeneration, ageing and disease. Nature Reviews Molecular Cell Biology, 17(5), 267–279. 26956195 10.1038/nrm.2016.7 PMC4918817

[wsbm1462-bib-0010] Amano, H. , Kato, S. , Ito, Y. , Eshima, K. , Ogawa, F. , Takahashi, R. , … Majima, M . (2015). The role of vascular endothelial growth factor receptor‐1 signaling in the recovery from ischemia. PLoS One, 10(7), e0131445. 26133989 10.1371/journal.pone.0131445 PMC4489890

[wsbm1462-bib-0011] Amthor, H. , Otto, A. , Vulin, A. , Rochat, A. , Dumonceaux, J. , Garcia, L. , … Partridge, T. (2009). Muscle hypertrophy driven by myostatin blockade does not require stem/precursor‐cell activity. Proceedings of the National Academy of Sciences, 106(18), 7479–7484. 10.1073/pnas.0811129106 PMC2671322 19383783

[wsbm1462-bib-0012] Anderson, D. M. , Anderson, K. M. , Chang, C.‐L. , Makarewich, C. A. , Nelson, B. R. , McAnally, J. R. , … Olson, E. N. (2015). A micropeptide encoded by a putative long noncoding RNA regulates muscle performance. Cell, 160(4), 595–606. 25640239 10.1016/j.cell.2015.01.009 PMC4356254

[wsbm1462-bib-0013] Andrews, S. J. , & Rothnagel, J. A. (2014). Emerging evidence for functional peptides encoded by short open reading frames. Nature Reviews Genetics, 15(3), 193–204. 10.1038/nrg3520 24514441

[wsbm1462-bib-0014] Anglister, L. , & McMahan, U. (1985). Basal lamina directs acetylcholinesterase accumulation at synaptic sites in regenerating muscle. Journal of Cell Biology, 101(3), 735–743. 3875617 10.1083/jcb.101.3.735 PMC2113729

[wsbm1462-bib-0015] Arikawa‐Hirasawa, E. , Rossi, S. G. , Rotundo, R. L. , & Yamada, Y. (2002). Absence of acetylcholinesterase at the neuromuscular junctions of perlecan‐null mice. Nature Neuroscience, 5(2), 119–123. 11802174 10.1038/nn801

[wsbm1462-bib-0016] Arnold, L. , Henry, A. , Poron, F. , Baba‐Amer, Y. , van Rooijen, N. , Plonquet, A. , … Chazaud, B. (2007). Inflammatory monocytes recruited after skeletal muscle injury switch into antiinflammatory macrophages to support myogenesis. Journal of Experimental Medicine, 204(5), 1057–1069. 17485518 10.1084/jem.20070075 PMC2118577

[wsbm1462-bib-0017] Arnold, S. J. , & Robertson, E. J. (2009). Making a commitment: Cell lineage allocation and axis patterning in the early mouse embryo. Nature Reviews Molecular Cell Biology, 10(2), 91–103. 19129791 10.1038/nrm2618

[wsbm1462-bib-0018] Arpino, V. , Brock, M. , & Gill, S. E. (2015). The role of TIMPs in regulation of extracellular matrix proteolysis. Matrix Biology, 44, 247–254. 25805621 10.1016/j.matbio.2015.03.005

[wsbm1462-bib-0019] Arsic, N. , Zacchigna, S. , Zentilin, L. , Ramirez‐Correa, G. , Pattarini, L. , Salvi, A. , … Giacca, M. (2004). Vascular endothelial growth factor stimulates skeletal muscle regeneration in vivo. Molecular Therapy, 10(5), 844–854. 15509502 10.1016/j.ymthe.2004.08.007

[wsbm1462-bib-0020] Ausoni, S. , Gorza, L. , Schiaffino, S. , Gundersen, K. , & Lomo, T. (1990). Expression of myosin heavy chain isoforms in stimulated fast and slow rat muscles. Journal of Neuroscience, 10(1), 153–160. 2405110 10.1523/JNEUROSCI.10-01-00153.1990 PMC6570340

[wsbm1462-bib-0021] Austin, R. L. , Rune, A. , Bouzakri, K. , Zierath, J. R. , & Krook, A. (2008). siRNA‐mediated reduction of inhibitor of nuclear factor‐κB kinase prevents tumor necrosis factor‐α‐induced insulin resistance in human skeletal muscle. Diabetes, 57(8), 2066–2073. 18443205 10.2337/db07-0763 PMC2494681

[wsbm1462-bib-0022] Autiero, M. , Luttun, A. , Tjwa, M. , & Carmeliet, P. (2003). Placental growth factor and its receptor, vascular endothelial growth factor receptor‐1: Novel targets for stimulation of ischemic tissue revascularization and inhibition of angiogenic and inflammatory disorders. Journal of Thrombosis and Haemostasis, 1(7), 1356–1370. 12871269 10.1046/j.1538-7836.2003.00263.x

[wsbm1462-bib-0023] Autiero, M. , Waltenberger, J. , Communi, D. , Kranz, A. , Moons, L. , Lambrechts, D. , … Kliche, S . (2003). Role of PlGF in the intra‐and intermolecular cross talk between the VEGF receptors Flt1 and Flk1. Nature Medicine, 9(7), 936. 10.1038/nm884 12796773

[wsbm1462-bib-0024] Avila, G. , Lee, E. H. , Perez, C. F. , Allen, P. D. , & Dirksen, R. T. (2003). FKBP12 binding to RyR1 modulates excitation–contraction coupling in mouse skeletal myotubes. Journal of Biological Chemistry, 278(25), 22600–22608. 12704193 10.1074/jbc.M205866200

[wsbm1462-bib-0025] Awad, S. S. , Lightowlers, R. N. , Young, C. , Chrzanowska‐Lightowlers, Z. M. , Lømo, T. , & Slater, C. R. (2001). Sodium channel mRNAs at the neuromuscular junction: Distinct patterns of accumulation and effects of muscle activity. Journal of Neuroscience, 21(21), 8456–8463. 11606634 10.1523/JNEUROSCI.21-21-08456.2001 PMC6762790

[wsbm1462-bib-0026] Aya, K. L. , & Stern, R. (2014). Hyaluronan in wound healing: Rediscovering a major player. Wound Repair and Regeneration, 22(5), 579–593. 25039417 10.1111/wrr.12214

[wsbm1462-bib-0027] Ayalon, G. , Davis, J. Q. , Scotland, P. B. , & Bennett, V. (2008). An ankyrin‐based mechanism for functional organization of dystrophin and dystroglycan. Cell, 135(7), 1189–1200. 19109891 10.1016/j.cell.2008.10.018

[wsbm1462-bib-0028] Ayalon, G. , Hostettler, J. D. , Hoffman, J. , Kizhatil, K. , Davis, J. Q. , & Bennett, V. (2011). Ankyrin‐B interactions with spectrin and dynactin‐4 are required for dystrophin‐based protection of skeletal muscle from exercise injury. Journal of Biological Chemistry, 286(9), 7370–7378. 21186323 10.1074/jbc.M110.187831 PMC3044993

[wsbm1462-bib-0029] Bagchi, R. A. , Roche, P. , Aroutiounova, N. , Espira, L. , Abrenica, B. , Schweitzer, R. , & Czubryt, M. P. (2016). The transcription factor scleraxis is a critical regulator of cardiac fibroblast phenotype. BMC Biology, 14(1), 21. 26988708 10.1186/s12915-016-0243-8 PMC4794909

[wsbm1462-bib-0030] Bailey, J. L. , Zheng, B. , Hu, Z. , Price, S. R. , & Mitch, W. E. (2006). Chronic kidney disease causes defects in signaling through the insulin receptor substrate/phosphatidylinositol 3‐kinase/Akt pathway: Implications for muscle atrophy. Journal of the American Society of Nephrology, 17(5), 1388–1394. 16611720 10.1681/ASN.2004100842

[wsbm1462-bib-0031] Baird, M. F. , Graham, S. M. , Baker, J. S. , & Bickerstaff, G. F. (2012). Creatine‐kinase‐ and exercise‐related muscle damage implications for muscle performance and recovery. Journal of Nutrition and Metabolism, 2012, 1–13. 10.1155/2012/960363 PMC3263635 22288008

[wsbm1462-bib-0032] Baker, J. S. , McCormick, M. C. , & Robergs, R. A. (2010). Interaction among skeletal muscle metabolic energy systems during intense exercise. Journal of Nutrition and Metabolism, 2010, 1–13. 10.1155/2010/905612 PMC3005844 21188163

[wsbm1462-bib-0033] Ban, C. R. , & Twigg, S. M. (2008). Fibrosis in diabetes complications: Pathogenic mechanisms and circulating and urinary markers. Vascular Health and Risk Management, 4(3), 575. 18827908 10.2147/vhrm.s1991 PMC2515418

[wsbm1462-bib-0034] Bang, M.‐L. , Mudry, R. E. , McElhinny, A. S. , Trombitás, K. , Geach, A. J. , Yamasaki, R. , … Labeit, S. (2001). Myopalladin, a novel 145‐kilodalton sarcomeric protein with multiple roles in Z‐disc and I‐band protein assemblies. Journal of Cell Biology, 153(2), 413–428. 11309420 10.1083/jcb.153.2.413 PMC2169455

[wsbm1462-bib-0035] Bartoli, M. , & Richard, I. (2005). Calpains in muscle wasting. International Journal of Biochemistry & Cell Biology, 37(10), 2115–2133. 16125114 10.1016/j.biocel.2004.12.012

[wsbm1462-bib-0036] Baum, J. , & Duffy, H. S. (2011). Fibroblasts and myofibroblasts: What are we talking about? Journal of Cardiovascular Pharmacology, 57(4), 376–379. 21297493 10.1097/FJC.0b013e3182116e39 PMC3077448

[wsbm1462-bib-0037] Beard, N. A. , Laver, D. R. , & Dulhunty, A. (2004). Calsequestrin and the calcium release channel of skeletal and cardiac muscle. Progress in Biophysics and Molecular Biology, 85(1), 33–69. 15050380 10.1016/j.pbiomolbio.2003.07.001

[wsbm1462-bib-0038] Beard, N. A. , Casarotto, M. G. , Wei, L. , Varsányi, M. , Laver, D. R. , & Dulhunty, A. F. (2005). Regulation of ryanodine receptors by calsequestrin: Effect of high luminal Ca^2+^ and phosphorylation. Biophysical Journal, 88(5), 3444–3454. 15731387 10.1529/biophysj.104.051441 PMC1305491

[wsbm1462-bib-0039] Beedle, A. M. (2016). Distribution of myosin heavy chain isoforms in muscular dystrophy: Insights into disease pathology. Musculoskeletal Regeneration, 2, e1365. PMC4943764 27430020

[wsbm1462-bib-0040] Belizário, J. E. , Fontes‐Oliveira, C. C. , Borges, J. P. , Kashiabara, J. A. , & Vannier, E. (2016). Skeletal muscle wasting and renewal: A pivotal role of myokine IL‐6. Springerplus, 5(1), 619. 27330885 10.1186/s40064-016-2197-2 PMC4870483

[wsbm1462-bib-0041] Benezra, R. , Davis, R. L. , Lockshon, D. , Turner, D. L. , & Weintraub, H. (1990). The protein Id: A negative regulator of helix‐loop‐helix DNA binding proteins. Cell, 61(1), 49–59. 2156629 10.1016/0092-8674(90)90214-y

[wsbm1462-bib-0042] Bengal, E. , Perdiguero, E. , Serrano, A. L. , & Muñoz‐Cánoves, P. (2017). Rejuvenating stem cells to restore muscle regeneration in aging. F1000Research, 6, 76. 28163911 10.12688/f1000research.9846.1 PMC5271918

[wsbm1462-bib-0043] Bentzinger, C. F. , Wang, Y. X. , & Rudnicki, M. A. (2012). Building muscle: Molecular regulation of myogenesis. Cold Spring Harbor Perspectives in Biology, 4(2), a008342. 22300977 10.1101/cshperspect.a008342 PMC3281568

[wsbm1462-bib-0044] Beqqali, A. , Monshouwer‐Kloots, J. , Monteiro, R. , Welling, M. , Bakkers, J. , Ehler, E. , … Passier, R. (2010). CHAP is a newly identified Z‐disc protein essential for heart and skeletal muscle function. Journal of Cell Science, 123(7), 1141–1150. 20215401 10.1242/jcs.063859

[wsbm1462-bib-0045] Berg, J. M. , Tymoczko, J. L. , & Stryer, L. (2002). The utilization of fatty acids as fuel requires three stages of processing In Biochemistry (5th ed.). New York, NY: W. H. Freeman.

[wsbm1462-bib-0046] Bergeron, R. , Ren, J. M. , Cadman, K. S. , Moore, I. K. , Perret, P. , Pypaert, M. , … Shulman, G. I. (2001). Chronic activation of AMP kinase results in NRF‐1 activation and mitochondrial biogenesis. American Journal of Physiology‐Endocrinology and Metabolism, 281(6), E1340–E1346. 11701451 10.1152/ajpendo.2001.281.6.E1340

[wsbm1462-bib-0047] Berlett, B. S. , & Stadtman, E. R. (1997). Protein oxidation in aging, disease, and oxidative stress. Journal of Biological Chemistry, 272(33), 20313–20316. 9252331 10.1074/jbc.272.33.20313

[wsbm1462-bib-0048] Beyfuss, K. , & Hood, D. A. (2018). A systematic review of p53 regulation of oxidative stress in skeletal muscle. Redox Report, 23(1), 100–117. 29298131 10.1080/13510002.2017.1416773 PMC6748683

[wsbm1462-bib-0049] Bezakova, G. , & Ruegg, M. A. (2003). New insights into the roles of agrin. Nature Reviews Molecular Cell Biology, 4(4), 295–309. 12671652 10.1038/nrm1074

[wsbm1462-bib-0050] Biernacka, A. , Dobaczewski, M. , & Frangogiannis, N. G. (2011). TGF‐β signaling in fibrosis. Growth Factors, 29(5), 196–202. 21740331 10.3109/08977194.2011.595714 PMC4408550

[wsbm1462-bib-0051] Birchmeier, C. , & Brohmann, H. (2000). Genes that control the development of migrating muscle precursor cells. Current Opinion in Cell Biology, 12(6), 725–730. 11063939 10.1016/s0955-0674(00)00159-9

[wsbm1462-bib-0052] Biswas, S. K. (2016). Does the interdependence between oxidative stress and inflammation explain the antioxidant paradox? Oxidative Medicine and Cellular Longevity, 2016, 1–9. 10.1155/2016/5698931 PMC4736408 26881031

[wsbm1462-bib-0053] Bjornson, C. R. , Cheung, T. H. , Liu, L. , Tripathi, P. V. , Steeper, K. M. , & Rando, T. A. (2012). Notch signaling is necessary to maintain quiescence in adult muscle stem cells. Stem Cells, 30(2), 232–242. 22045613 10.1002/stem.773 PMC3384696

[wsbm1462-bib-0054] Blanco‐Bose, W. E. , Yao, C. C. , Kramer, R. H. , & Blau, H. M. (2001). Purification of mouse primary myoblasts based on alpha 7 integrin expression. Experimental Cell Research, 265(2), 212–220. 10.1006/excr.2001.5191 11302686

[wsbm1462-bib-0055] Blizzard LeBlanc, D. R. , Rioux, B. V. , Pelech, C. , Moffatt, T. L. , Kimber, D. E. , Duhamel, T. A. , … Sénéchal, M . (2017). Exercise‐induced irisin release as a determinant of the metabolic response to exercise training in obese youth: The EXIT trial. Physiological Reports, 5(23), e13539. 10.14814/phy2.13539 PMC5727287 29208692

[wsbm1462-bib-0056] Bodine, S. C. , & Baehr, L. M. (2014). Skeletal muscle atrophy and the E3 ubiquitin ligases MuRF1 and MAFbx/atrogin‐1. American Journal of Physiology‐Endocrinology and Metabolism, 307(6), E469–E484. 25096180 10.1152/ajpendo.00204.2014 PMC4166716

[wsbm1462-bib-0057] Bodine, S. C. , Latres, E. , Baumhueter, S. , Lai, V. K. , Nunez, L. , Clarke, B. A. , … Glass, D. J. (2001). Identification of ubiquitin ligases required for skeletal muscle atrophy. Science, 294(5547), 1704–1708. 11679633 10.1126/science.1065874

[wsbm1462-bib-0058] Bonne, G. , Carrier, L. , Richard, P. , Hainque, B. , & Schwartz, K. (1998). Familial hypertrophic cardiomyopathy: From mutations to functional defects. Circulation Research, 83(6), 580–593. 9742053 10.1161/01.res.83.6.580

[wsbm1462-bib-0059] Boonyarom, O. , & Inui, K. (2006). Atrophy and hypertrophy of skeletal muscles: Structural and functional aspects. Acta Physiologica, 188(2), 77–89. 16948795 10.1111/j.1748-1716.2006.01613.x

[wsbm1462-bib-0060] Boppart, M. D. , Burkin, D. J. , & Kaufman, S. J. (2006). α7β1‐Integrin regulates mechanotransduction and prevents skeletal muscle injury. American Journal of Physiology: Cell Physiology, 290(6), C1660–C1665. 16421207 10.1152/ajpcell.00317.2005

[wsbm1462-bib-0061] Bowe, M. A. , Mendis, D. B. , & Fallon, J. R. (2000). The small leucine‐rich repeat proteoglycan biglycan binds to α‐dystroglycan and is upregulated in dystrophic muscle. Journal of Cell Biology, 148(4), 801–810. 10684260 10.1083/jcb.148.4.801 PMC2169361

[wsbm1462-bib-0062] Brack, A. S. , & Muñoz‐Cánoves, P. (2015). The ins and outs of muscle stem cell aging. Skeletal Muscle, 6(1), 1. 10.1186/s13395-016-0072-z PMC4716636 26783424

[wsbm1462-bib-0063] Brandt, C. , & Pedersen, B. K. (2010). The role of exercise‐induced myokines in muscle homeostasis and the defense against chronic diseases. Journal of Biomedicine and Biotechnology, 2010, 6. 10.1155/2010/520258 PMC2836182 20224659

[wsbm1462-bib-0064] Brandt, N. R. , & Caswell, A. H. (1999). Localization of mitsugumin 29 to transverse tubules in rabbit skeletal muscle. Archives of Biochemistry and Biophysics, 371(2), 348–350. 10545225 10.1006/abbi.1999.1444

[wsbm1462-bib-0065] Brioche, T. , & Lemoine‐Morel, S. (2016). Oxidative stress, sarcopenia, antioxidant strategies and exercise: Molecular aspects. Current Pharmaceutical Design, 22(18), 2664–2678. 26891808 10.2174/1381612822666160219120531

[wsbm1462-bib-0066] Buas, M. F. , & Kadesch, T. (2010). Regulation of skeletal myogenesis by Notch. Experimental Cell Research, 316(18), 3028–3033. 20452344 10.1016/j.yexcr.2010.05.002 PMC4268546

[wsbm1462-bib-0067] Buckingham, M. (2007). Skeletal muscle progenitor cells and the role of Pax genes. Comptes Rendus Biologies, 330(6), 530–533. 17631448 10.1016/j.crvi.2007.03.015

[wsbm1462-bib-0068] Buller, A. , Mommaerts, W. , & Seraydarian, K. (1969). Enzymic properties of myosin in fast and slow twitch muscles of the cat following cross‐innervation. Journal of Physiology, 205(3), 581–597. 4243389 10.1113/jphysiol.1969.sp008984 PMC1348570

[wsbm1462-bib-0069] Byles, V. , Covarrubias, A. J. , Ben‐Sahra, I. , Lamming, D. W. , Sabatini, D. M. , Manning, B. D. , & Horng, T. (2013). The TSC‐mTOR pathway regulates macrophage polarization. Nature Communications, 4, 2834. 10.1038/ncomms3834 PMC3876736 24280772

[wsbm1462-bib-0070] Cachofeiro, V. , Goicochea, M. , De Vinuesa, S. G. , Oubiña, P. , Lahera, V. , & Luño, J. (2008). Oxidative stress and inflammation, a link between chronic kidney disease and cardiovascular disease: New strategies to prevent cardiovascular risk in chronic kidney disease. Kidney International, 74, S4–S9. 10.1038/ki.2008.516 19034325

[wsbm1462-bib-0071] Cai, Q. , & Sheng, Z.‐H. (2009). Molecular motors and synaptic assembly. The Neuroscientist, 15(1), 78–89. 19218232 10.1177/1073858408329511 PMC4955359

[wsbm1462-bib-0072] Calvo, J. A. , Daniels, T. G. , Wang, X. , Paul, A. , Lin, J. , Spiegelman, B. M. , … Rangwala, S. M. (2008). Muscle‐specific expression of PPARγ coactivator‐1α improves exercise performance and increases peak oxygen uptake. Journal of Applied Physiology, 104(5), 1304–1312. 18239076 10.1152/japplphysiol.01231.2007

[wsbm1462-bib-0073] Cantó, C. , & Auwerx, J. (2009). PGC‐1alpha, SIRT1 and AMPK, an energy sensing network that controls energy expenditure. Current Opinion in Lipidology, 20(2), 98–105. 19276888 10.1097/MOL.0b013e328328d0a4 PMC3627054

[wsbm1462-bib-0074] Carey, A. L. , Steinberg, G. R. , Macaulay, S. L. , Thomas, W. G. , Holmes, A. G. , Ramm, G. , … Febbraio, M. A. (2006). Interleukin‐6 increases insulin‐stimulated glucose disposal in humans and glucose uptake and fatty acid oxidation in vitro via AMP‐activated protein kinase. Diabetes, 55(10), 2688–2697. 17003332 10.2337/db05-1404

[wsbm1462-bib-0075] Carlsson, L. , Yu, J.‐G. , Moza, M. , Carpén, O. , & Thornell, L.‐E. (2007). Myotilin—A prominent marker of myofibrillar remodelling. Neuromuscular Disorders, 17(1), 61–68. 17056257 10.1016/j.nmd.2006.09.007

[wsbm1462-bib-0076] Carmignac, V. , & Durbeej, M. (2012). Cell–matrix interactions in muscle disease. Journal of Pathology, 226(2), 200–218. 21989954 10.1002/path.3020

[wsbm1462-bib-0077] Carnac, G. , Fajas, L. , L'honoré, A. , Sardet, C. , Lamb, N. J. , & Fernandez, A. (2000). The retinoblastoma‐like protein p130 is involved in the determination of reserve cells in differentiating myoblasts. Current Biology, 10(9), 543–546. 10801445 10.1016/s0960-9822(00)00471-1

[wsbm1462-bib-0078] Castanón, M. J. , Walko, G. , Winter, L. , & Wiche, G. (2013). Plectin‐intermediate filament partnership in skin, skeletal muscle, and peripheral nerve. Histochemistry and Cell Biology, 140(1), 33–53. 23748243 10.1007/s00418-013-1102-0 PMC3695321

[wsbm1462-bib-0079] Chapman, M. A. , Mukund, K. , Subramaniam, S. , Brenner, D. , & Lieber, R. L. (2017). Three distinct cell populations express extracellular matrix proteins and increase in number during skeletal muscle fibrosis. American Journal of Physiology: Cell Physiology, 312(2), C131–C143. 27881411 10.1152/ajpcell.00226.2016 PMC5336596

[wsbm1462-bib-0080] Chen, H.‐H. , Chen, W.‐P. , Yan, W.‐L. , Huang, Y. C. , Chang, S. W. , Fu, W. M. , … Chen, S. L. (2015). NRIP is newly identified as a Z‐disc protein, activating calmodulin signaling for skeletal muscle contraction and regeneration. Journal of Cell Science, 128(22), 4196–4209. 26430214 10.1242/jcs.174441

[wsbm1462-bib-0081] Chin, E. R. (2005). Role of Ca^2+^/calmodulin‐dependent kinases in skeletal muscle plasticity. Journal of Applied Physiology, 99(2), 414–423. 16020436 10.1152/japplphysiol.00015.2005

[wsbm1462-bib-0082] Chin, E. R. , Olson, E. N. , Richardson, J. A. , Yang, Q. , Humphries, C. , Shelton, J. M. , … Williams, R. S. (1998). A calcineurin‐dependent transcriptional pathway controls skeletal muscle fiber type. Genes & Development, 12(16), 2499–2509. 9716403 10.1101/gad.12.16.2499 PMC317085

[wsbm1462-bib-0083] Choi, H. Y. , Liu, Y. , Tennert, C. , Sugiura, Y. , Karakatsani, A. , Kröger, S. , … Herz, J. (2013). APP interacts with LRP4 and agrin to coordinate the development of the neuromuscular junction in mice. eLife, 2, e00220. 10.7554/eLife.00220 PMC3748711 23986861

[wsbm1462-bib-0084] Choi, M. H. , Ow, J. R. , Yang, N.‐D. , & Taneja, R. (2016). Oxidative stress‐mediated skeletal muscle degeneration: Molecules, mechanisms, and therapies. Oxidative Medicine and Cellular Longevity, 2016, 1–13. 10.1155/2016/6842568 PMC4700198 26798425

[wsbm1462-bib-0085] Christov, C. , Chrétien, F. , Abou‐Khalil, R. , Bassez, G. , Vallet, G. , Authier, F. J. , … Gherardi, R. K. (2007). Muscle satellite cells and endothelial cells: Close neighbors and privileged partners. Molecular Biology of the Cell, 18(4), 1397–1409. 17287398 10.1091/mbc.E06-08-0693 PMC1838982

[wsbm1462-bib-0086] Clark, K. A. , McElhinny, A. S. , Beckerle, M. C. , & Gregorio, C. C. (2002). Striated muscle cytoarchitecture: An intricate web of form and function. Annual Review of Cell and Developmental Biology, 18(1), 637–706. 10.1146/annurev.cellbio.18.012502.105840 12142273

[wsbm1462-bib-0087] Coley, W. , Rayavarapu, S. , Pandey, G. S. , Sabina, R. L. , van der Meulen, J. H. , Ampong, B. , … Nagaraju, K. (2012). The molecular basis of skeletal muscle weakness in a mouse model of inflammatory myopathy. Arthritis and Rheumatism, 64(11), 3750–3759. 22806328 10.1002/art.34625 PMC3485437

[wsbm1462-bib-0088] Constantin, B. (2014). Dystrophin complex functions as a scaffold for signalling proteins. Biochimica et Biophysica Acta, 1838(2), 635–642. 24021238 10.1016/j.bbamem.2013.08.023

[wsbm1462-bib-0089] Conte, E. , Fruciano, M. , Fagone, E. , Gili, E. , Caraci, F. , Iemmolo, M. , … Vancheri, C. (2011). Inhibition of PI3K prevents the proliferation and differentiation of human lung fibroblasts into myofibroblasts: The role of class I P110 isoforms. PLoS One, 6(10), e24663. 21984893 10.1371/journal.pone.0024663 PMC3184941

[wsbm1462-bib-0090] Cooper, R. , Tajbakhsh, S. , Mouly, V. , Cossu, G. , Buckingham, M. , & Butler‐Browne, G. (1999). In vivo satellite cell activation via Myf5 and MyoD in regenerating mouse skeletal muscle. Journal of Cell Science, 112(17), 2895–2901. 10444384 10.1242/jcs.112.17.2895

[wsbm1462-bib-0091] Cozzolino, M. , & Carrì, M. T. (2012). Mitochondrial dysfunction in ALS. Progress in Neurobiology, 97(2), 54–66. 10.1016/j.pneurobio.2011.06.003 21827820

[wsbm1462-bib-0092] Crum‐Cianflone, N. F. (2008). Bacterial, fungal, parasitic, and viral myositis. Clinical Microbiology Reviews, 21(3), 473–494. 18625683 10.1128/CMR.00001-08 PMC2493084

[wsbm1462-bib-0093] Cudmore, M. J. , Hewett, P. W. , Ahmad, S. , Wang, K. Q. , Cai, M. , al‐Ani, B. , … Ahmed, A. (2012). The role of heterodimerization between VEGFR‐1 and VEGFR‐2 in the regulation of endothelial cell homeostasis. Nature Communications, 3, 972. 10.1038/ncomms1977 22828632

[wsbm1462-bib-0094] DeBerardinis, R. J. , Mancuso, A. , Daikhin, E. , et al. (2007). Beyond aerobic glycolysis: Transformed cells can engage in glutamine metabolism that exceeds the requirement for protein and nucleotide synthesis. Proceedings of the National Academy of Sciences, 104(49), 19345–19350. 10.1073/pnas.0709747104 PMC2148292 18032601

[wsbm1462-bib-0095] Denker, A. , & Rizzoli, S. O. (2010). Synaptic vesicle pools: An update. Frontiers in Synaptic Neuroscience, 2, 135. 21423521 10.3389/fnsyn.2010.00135 PMC3059705

[wsbm1462-bib-0096] Di Rienzo, M. , Antonioli, M. , Fusco, C. , Liu, Y. , Mari, M. , Orhon, I. , … Ciccosanti, F . (2019). Autophagy induction in atrophic muscle cells requires ULK1 activation by TRIM32 through unanchored K63‐linked polyubiquitin chains. Science Advances, 5(5), eaau8857. 31123703 10.1126/sciadv.aau8857 PMC6527439

[wsbm1462-bib-0097] Doles, J. D. , & Olwin, B. B. (2014). The impact of JAK‐STAT signaling on muscle regeneration. Nature Medicine, 20(10), 1094–1095. 10.1038/nm.3720 PMC4426494 25295935

[wsbm1462-bib-0098] Doucet, M. , Russell, A. P. , Léger, B. , Debigaré, R. , Joanisse, D. R. , Caron, M. A. , … Maltais, F. (2007). Muscle atrophy and hypertrophy signaling in patients with chronic obstructive pulmonary disease. American Journal of Respiratory and Critical Care Medicine, 176(3), 261–269. 17478621 10.1164/rccm.200605-704OC

[wsbm1462-bib-0099] Du, J. , Wang, X. , Miereles, C. , Bailey, J. L. , Debigare, R. , Zheng, B. , … Mitch, W. E . (2004). Activation of caspase‐3 is an initial step triggering accelerated muscle proteolysis in catabolic conditions. Journal of Clinical Investigation, 113(1), 115–123. 14702115 10.1172/JCI200418330 PMC300763

[wsbm1462-bib-0100] Dumont, N. A. , Wang, Y. X. , & Rudnicki, M. A. (2015). Intrinsic and extrinsic mechanisms regulating satellite cell function. Development, 142(9), 1572–1581. 25922523 10.1242/dev.114223 PMC4419274

[wsbm1462-bib-0101] Dupuis, L. , & Loeffler, J.‐P. (2009). Neuromuscular junction destruction during amyotrophic lateral sclerosis: Insights from transgenic models. Current Opinion in Pharmacology, 9(3), 341–346. 10.1016/j.coph.2009.03.007 19386549

[wsbm1462-bib-0102] Durham, J. T. , Brand, O. M. , Arnold, M. , Reynolds, J. G. , Muthukumar, L. , Weiler, H. , … Naya, F. J. (2006). Myospryn is a direct transcriptional target for MEF2A that encodes a striated muscle, α‐actinin‐interacting, costamere‐localized protein. Journal of Biological Chemistry, 281(10), 6841–6849. 16407236 10.1074/jbc.M510499200

[wsbm1462-bib-0103] Eaton, S. , Bartlett, K. , & Pourfarzam, M. (1996). Mammalian mitochondrial beta‐oxidation. Biochemical Journal, 320(Pt. 2), 345–357. 8973539 10.1042/bj3200345 PMC1217938

[wsbm1462-bib-0104] Ellingsgaard, H. , Hauselmann, I. , Schuler, B. , Habib, A. M. , Baggio, L. L. , Meier, D. T. , … Donath, M. Y. (2011). Interleukin‐6 enhances insulin secretion by increasing glucagon‐like peptide‐1 secretion from L cells and alpha cells. Nature Medicine, 17(11), 1481–1489. 10.1038/nm.2513 PMC4286294 22037645

[wsbm1462-bib-0105] Engel, A. G. (2014). Congenital myasthenic syndromes In Darras B. H., Jones R. Jr, Ryan M., & De Vivo D., (Eds.), Neuromuscular disorders of infancy, childhood, and adolescence (2nd ed., pp. 456–481). San Diego, CA: Elsevier.

[wsbm1462-bib-0106] Espinoza‐Fonseca, L. M. , Autry, J. M. , & Thomas, D. D. (2015). Sarcolipin and phospholamban inhibit the calcium pump by populating a similar metal ion‐free intermediate state. Biochemical and Biophysical Research Communications, 463(1), 37–41. 25983321 10.1016/j.bbrc.2015.05.012 PMC4465059

[wsbm1462-bib-0107] Evans, R. A. , Tian, Y. C. , Steadman, R. , & Phillips, A. O. (2003). TGF‐β1‐mediated fibroblast–myofibroblast terminal differentiation—The role of smad proteins. Experimental Cell Research, 282(2), 90–100. 12531695 10.1016/s0014-4827(02)00015-0

[wsbm1462-bib-0108] Even, P. C. , Decrouy, A. , & Chinet, A. (1994). Defective regulation of energy metabolism in mdx‐mouse skeletal muscles. Biochemical Journal, 304(Pt. 2), 649–654. 7999003 10.1042/bj3040649 PMC1137540

[wsbm1462-bib-0109] Fatica, A. , & Bozzoni, I. (2014). Long non‐coding RNAs: New players in cell differentiation and development. Nature Reviews Genetics, 15(1), 7–21. 10.1038/nrg3606 24296535

[wsbm1462-bib-0110] Febbraio, M. A. , & Pedersen, B. K. (2005). Contraction‐induced myokine production and release: Is skeletal muscle an endocrine organ? Exercise and Sport Sciences Reviews, 33(3), 114–119. 16006818 10.1097/00003677-200507000-00003

[wsbm1462-bib-0111] Ferrara, N. , Carver‐Moore, K. , Chen, H. , Dowd, M. , Lu, L. , O'Shea, K. S. , … Moore, M. W. (1996). Heterozygous embryonic lethality induced by targeted inactivation of the VEGF gene. Nature, 380(6573), 439–442. 8602242 10.1038/380439a0

[wsbm1462-bib-0112] Fink, L. N. , Oberbach, A. , Costford, S. R. , Chan, K. L. , Sams, A. , Blüher, M. , & Klip, A . (2013). Expression of anti‐inflammatory macrophage genes within skeletal muscle correlates with insulin sensitivity in human obesity and type 2 diabetes. Diabetologia, 56(7), 1623–1628. 23595247 10.1007/s00125-013-2897-x

[wsbm1462-bib-0113] Fitts, R. H. (2008). The cross‐bridge cycle and skeletal muscle fatigue. Journal of Applied Physiology, 104(2), 551–558. 18162480 10.1152/japplphysiol.01200.2007

[wsbm1462-bib-0114] Flaumenhaft, R. , & Rifkin, D. B. (1991). Extracellular matrix regulation of growth factor and protease activity. Current Opinion in Cell Biology, 3(5), 817–823. 1931082 10.1016/0955-0674(91)90055-4

[wsbm1462-bib-0115] Flick, M. J. , & Konieczny, S. F. (2000). The muscle regulatory and structural protein MLP is a cytoskeletal binding partner of betaI‐spectrin. Journal of Cell Science, 113(9), 1553–1564. 10751147 10.1242/jcs.113.9.1553

[wsbm1462-bib-0116] Flucher, B. , & Daniels, M. (1989). Distribution of Na+ channels and ankyrin in neuromuscular junctions is complementary to that of acetylcholine receptors and the 43 kd protein. Neuron, 3(2), 163–175. 2560390 10.1016/0896-6273(89)90029-9

[wsbm1462-bib-0117] Foletta, V. C. , White, L. J. , Larsen, A. E. , Léger, B. , & Russell, A. P. (2011). The role and regulation of MAFbx/atrogin‐1 and MuRF1 in skeletal muscle atrophy. Pflügers Archiv‐European Journal of Physiology, 461(3), 325–335. 21221630 10.1007/s00424-010-0919-9

[wsbm1462-bib-0118] Fox, M. A. , Ho, M. S. , Smyth, N. , & Sanes, J. R. (2008). A synaptic nidogen: Developmental regulation and role of nidogen‐2 at the neuromuscular junction. Neural Development, 3(1), 1–17. 10.1186/1749-8104-3-24 PMC2567315 18817539

[wsbm1462-bib-0119] Francetic, T. , & Li, Q. (2011). Skeletal myogenesis and Myf5 activation. Transcription, 2(3), 109–114. 21922054 10.4161/trns.2.3.15829 PMC3173648

[wsbm1462-bib-0120] Francis, S. H. , Busch, J. L. , & Corbin, J. D. (2010). cGMP‐dependent protein kinases and cGMP phosphodiesterases in nitric oxide and cGMP action. Pharmacological Reviews, 62(3), 525–563. 20716671 10.1124/pr.110.002907 PMC2964902

[wsbm1462-bib-0121] Franckhauser, S. , Elias, I. , Sopasakis, V. R. , Ferre, T. , Nagaev, I. , Andersson, C. X. , … Smith, U . (2008). Overexpression of Il6 leads to hyperinsulinaemia, liver inflammation and reduced body weight in mice. Diabetologia, 51(7), 1306–1316. 18437347 10.1007/s00125-008-0998-8

[wsbm1462-bib-0122] Franzini‐Armstrong, C. (2004). Functional implications of RyR‐dHPR relationships in skeletal and cardiac muscles. Biological Research, 37(4), 507–512. 15709676 10.4067/s0716-97602004000400003

[wsbm1462-bib-0123] Freiburg, A. , & Gautel, M. (1996). A molecular map of the interactions between titin and myosin‐binding protein C. FEBS Journal, 235(1–2), 317–323. 10.1111/j.1432-1033.1996.00317.x 8631348

[wsbm1462-bib-0124] Fukada, S. , Uezumi, A. , Ikemoto, M. , Masuda, S. , Segawa, M. , Tanimura, N. , … Takeda, S.'. (2007). Molecular signature of quiescent satellite cells in adult skeletal muscle. Stem Cells, 25(10), 2448–2459. 17600112 10.1634/stemcells.2007-0019

[wsbm1462-bib-0125] Fukui, M. , Nakamura, T. , Ebihara, I. , Shirato, I. , Tomino, Y. , & Koide, H. (1992). ECM gene expression and its modulation by insulin in diabetic rats. Diabetes, 41(12), 1520–1527. 1280237 10.2337/diab.41.12.1520

[wsbm1462-bib-0126] Fukunaga, H. , Engel, A. G. , Osame, M. , & Lambert, E. H. (1982). Paucity and disorganization of presynaptic membrane active zones in the Lambert‐Eaton myasthenic syndrome. Muscle & Nerve, 5(9), 686–697.

[wsbm1462-bib-0127] Galińska‐Rakoczy, A. , Engel, P. , Xu, C. , Jung, H. S. , Craig, R. , Tobacman, L. S. , & Lehman, W. (2008). Structural basis for the regulation of muscle contraction by troponin and tropomyosin. Journal of Molecular Biology, 379(5), 929–935. 18514658 10.1016/j.jmb.2008.04.062 PMC2483953

[wsbm1462-bib-0128] Gamage, D. G. , Leikina, E. , Quinn, M. E. , Ratinov, A. , Chernomordik, L. V. , & Millay, D. P. (2017). Insights into the localization and function of myomaker during myoblast fusion. Journal of Biological Chemistry, 292, 17272–17289. 28860190 10.1074/jbc.M117.811372 PMC5655506

[wsbm1462-bib-0129] Gautel, M. , & Djinović‐Carugo, K. (2016). The sarcomeric cytoskeleton: From molecules to motion. Journal of Experimental Biology, 219(2), 135–145. 26792323 10.1242/jeb.124941

[wsbm1462-bib-0130] Gelse, K. , Pöschl, E. , & Aigner, T. (2003). Collagens—Structure, function, and biosynthesis. Advanced Drug Delivery Reviews, 55(12), 1531–1546. 14623400 10.1016/j.addr.2003.08.002

[wsbm1462-bib-0131] Gerhardt, H. (2008). VEGF and endothelial guidance in angiogenic sprouting In VEGF in development (pp. 68–78). New York, NY: Springer. 10.4161/org.4.4.7414 PMC2634329 19337404

[wsbm1462-bib-0132] Ghosh, D. , Syed, A. , Prada, M. , Nystoriak, M. A. , Santana, L. F. , Nieves‐Cintrón, M. , & Navedo, M. F. (2017). Calcium channels in vascular smooth muscle. Advances in Pharmacology, 78, 49–87. 28212803 10.1016/bs.apha.2016.08.002 PMC5439506

[wsbm1462-bib-0133] Gilbert, A. , Wyczalkowska‐Tomasik, A. , Zendzian‐Piotrowska, M. , & Czarkowska‐Paczek, B. (2016). Training differentially regulates elastin level and proteolysis in skeletal and heart muscles and aorta in healthy rats. Biology Open, 5(5), 556–562. 27069251 10.1242/bio.017459 PMC4874357

[wsbm1462-bib-0134] Gilbert, R. , Cohen, J. A. , Pardo, S. , Basu, A. , & Fischman, D. A. (1999). Identification of the A‐band localization domain of myosin binding proteins C and H (MyBP‐C, MyBP‐H) in skeletal muscle. Journal of Cell Science, 112(1), 69–79. 9841905 10.1242/jcs.112.1.69

[wsbm1462-bib-0135] Gillies, A. R. , & Lieber, R. L. (2011). Structure and function of the skeletal muscle extracellular matrix. Muscle & Nerve, 44(3), 318–331. 21949456 10.1002/mus.22094 PMC3177172

[wsbm1462-bib-0136] Giordani, L. , & Puri, P. L. (2013). Epigenetic control of skeletal muscle regeneration. FEBS Journal, 280(17), 4014–4025. 23745685 10.1111/febs.12383 PMC3753079

[wsbm1462-bib-0137] Glicksman, M. A. , & Sanes, J. R. (1983). Differentiation of motor nerve terminals formed in the absence of muscle fibres. Journal of Neurocytology, 12(4), 661–671. 6352869 10.1007/BF01181529

[wsbm1462-bib-0138] Gnaiger, E. (2009). Capacity of oxidative phosphorylation in human skeletal muscle: New perspectives of mitochondrial physiology. International Journal of Biochemistry & Cell Biology, 41(10), 1837–1845. 19467914 10.1016/j.biocel.2009.03.013

[wsbm1462-bib-0139] Gokhin, D. S. , Ochala, J. , Domenighetti, A. A. , & Fowler, V. M. (2015). Tropomodulin 1 directly controls thin filament length in both wild‐type and tropomodulin 4‐deficient skeletal muscle. Development, 142(24), 4351–4362. 26586224 10.1242/dev.129171 PMC4689220

[wsbm1462-bib-0140] Goldfarb, L. , Vicart, P. , Goebel, H. , & Dalakas, M. (2004). Desmin myopathy. Brain, 127(4), 723–734. 14724127 10.1093/brain/awh033

[wsbm1462-bib-0141] Gomes, M. D. , Lecker, S. H. , Jagoe, R. T. , Navon, A. , & Goldberg, A. L. (2001). Atrogin‐1, a muscle‐specific F‐box protein highly expressed during muscle atrophy. Proceedings of the National Academy of Sciences, 98(25), 14440–14445. 10.1073/pnas.251541198 PMC64700 11717410

[wsbm1462-bib-0142] Gonçalves, T. J. , & Armand, A.‐S. (2017). Non‐coding RNAs in skeletal muscle regeneration. Non‐coding RNA Research, 2(1), 56–67. 30159421 10.1016/j.ncrna.2017.03.003 PMC6096429

[wsbm1462-bib-0143] Gontier, Y. , Taivainen, A. , Fontao, L. , Sonnenberg, A. , van der Flier, A. , Carpen, O. , … Borradori, L. (2005). The Z‐disc proteins myotilin and FATZ‐1 interact with each other and are connected to the sarcolemma via muscle‐specific filamins. Journal of Cell Science, 118(16), 3739–3749. 16076904 10.1242/jcs.02484

[wsbm1462-bib-0144] Gopinath, S. D. , Webb, A. E. , Brunet, A. , & Rando, T. A. (2014). FOXO3 promotes quiescence in adult muscle stem cells during the process of self‐renewal. Stem Cell Reports, 2(4), 414–426. 24749067 10.1016/j.stemcr.2014.02.002 PMC3986584

[wsbm1462-bib-0145] Gordon, M. K. , & Hahn, R. A. (2010). Collagens. Cell and Tissue Research, 339(1), 247–257. 19693541 10.1007/s00441-009-0844-4 PMC2997103

[wsbm1462-bib-0146] Green, D. J. , Spence, A. , Rowley, N. , Thijssen, D. H. , & Naylor, L. H. (2012). Vascular adaptation in athletes: Is there an 'athlete's artery'? Experimental Physiology, 97(3), 295–304. 22179421 10.1113/expphysiol.2011.058826

[wsbm1462-bib-0147] Grifone, R. , Demignon, J. , Houbron, C. , Souil, E. , Niro, C. , Seller, M. J. , … Maire, P. (2005). Six1 and Six4 homeoproteins are required for Pax3 and Mrf expression during myogenesis in the mouse embryo. Development, 132(9), 2235–2249. 10.1242/dev.01773 15788460

[wsbm1462-bib-0148] Grzelkowska‐Kowalczyk, K. (2016). The importance of extracellular matrix in skeletal muscle development and function In Composition and function of the extracellular matrix in the human body (pp. 3–24).

[wsbm1462-bib-0149] Günther, S. , Kim, J. , Kostin, S. , Lepper, C. , Fan, C.‐M. , & Braun, T. (2013). Myf5‐positive satellite cells contribute to Pax7‐dependent long‐term maintenance of adult muscle stem cells. Cell Stem Cell, 13(5), 590–601. 23933088 10.1016/j.stem.2013.07.016 PMC4082715

[wsbm1462-bib-0150] Guttridge, D. C. , Albanese, C. , Reuther, J. Y. , Pestell, R. G. , & Baldwin, A. S. (1999). NF‐κB controls cell growth and differentiation through transcriptional regulation of cyclin D1. Molecular and Cellular Biology, 19(8), 5785–5799. 10409765 10.1128/mcb.19.8.5785 PMC84428

[wsbm1462-bib-0151] Guttridge, D. C. , Mayo, M. W. , Madrid, L. V. , Wang, C.‐Y. , & Baldwin, A. S., Jr. (2000). NF‐κB‐induced loss of MyoD messenger RNA: Possible role in muscle decay and cachexia. Science, 289(5488), 2363–2366. 11009425 10.1126/science.289.5488.2363

[wsbm1462-bib-0152] Györke, I. , Hester, N. , Jones, L. R. , & Györke, S. (2004). The role of calsequestrin, triadin, and junctin in conferring cardiac ryanodine receptor responsiveness to luminal calcium. Biophysical Journal, 86(4), 2121–2128. 15041652 10.1016/S0006-3495(04)74271-X PMC1304063

[wsbm1462-bib-0153] Haddad, F. , Zaldivar, F. , Cooper, D. M. , & Adams, G. R. (2005). IL‐6‐induced skeletal muscle atrophy. Journal of Applied Physiology, 98(3), 911–917. 15542570 10.1152/japplphysiol.01026.2004

[wsbm1462-bib-0154] Hald, A. , & Lotharius, J. (2005). Oxidative stress and inflammation in Parkinson's disease: Is there a causal link? Experimental Neurology, 193(2), 279–290. 15869932 10.1016/j.expneurol.2005.01.013

[wsbm1462-bib-0155] Hall, Z. W. , & Sanes, J. R. (1993). Synaptic structure and development: The neuromuscular junction. Cell, 72, 99–121. 8428377 10.1016/s0092-8674(05)80031-5

[wsbm1462-bib-0156] Halper, J. , & Kjaer, M. (2014). Basic components of connective tissues and extracellular matrix: Elastin, fibrillin, fibulins, fibrinogen, fibronectin, laminin, tenascins and thrombospondins In Progress in heritable soft connective tissue diseases (pp. 31–47). Dordrecht, Netherlands: Springer. 10.1007/978-94-007-7893-1_3 24443019

[wsbm1462-bib-0157] Hamrick, M. W. (2011). A role for myokines in muscle‐bone interactions. Exercise and Sport Sciences Reviews, 39(1), 43–47. 21088601 10.1097/JES.0b013e318201f601 PMC3791922

[wsbm1462-bib-0158] Hamrick, M. W. (2012). The skeletal muscle secretome: An emerging player in muscle–bone crosstalk. Bonekey Reports, 1(4), 60. 23951457 10.1038/bonekey.2012.60 PMC3727847

[wsbm1462-bib-0159] Handschin, C. , Chin, S. , Li, P. , Liu, F. , Maratos‐Flier, E. , LeBrasseur, N. K. , … Spiegelman, B. M. (2007). Skeletal muscle fiber‐type switching, exercise intolerance and myopathy in PGC‐1α muscle‐specific knockout animals. Journal of Biological Chemistry, 282, 30014–30021. 17702743 10.1074/jbc.M704817200

[wsbm1462-bib-0160] Harada, H. , Hayashi, T. , Nishi, H. , Kusaba, K. , Koga, Y. , Koga, Y. , … Kimura, A. (2018). Phenotypic expression of a novel desmin gene mutation: Hypertrophic cardiomyopathy followed by systemic myopathy. Journal of Human Genetics, 63(2), 249–254. 29167554 10.1038/s10038-017-0383-x

[wsbm1462-bib-0161] Hardie, D. G. (2011). AMP‐activated protein kinase—An energy sensor that regulates all aspects of cell function. Genes & Development, 25(18), 1895–1908. 21937710 10.1101/gad.17420111 PMC3185962

[wsbm1462-bib-0162] Harlow, M. L. , Ress, D. , Stoschek, A. , Marshall, R. M. , & McMahan, U. J. (2001). The architecture of active zone material at the frog's neuromuscular junction. Nature, 409(6819), 479–484. 11206537 10.1038/35054000

[wsbm1462-bib-0163] Härönen, H. , Zainul, Z. , Tu, H. , Naumenko, N. , Sormunen, R. , Miinalainen, I. , … Pihlajaniemi, T. (2017). Collagen XIII secures pre‐and postsynaptic integrity of the neuromuscular synapse. Human Molecular Genetics, 26(11), 2076–2090. 28369367 10.1093/hmg/ddx101

[wsbm1462-bib-0164] Haskins, K. , Bradley, B. , Powers, K. , Fadok, V. , Flores, S. , Ling, X. , … Kench, J . (2003). Oxidative stress in type 1 diabetes. Annals of the New York Academy of Sciences, 1005(1), 43–54. 14679039 10.1196/annals.1288.006

[wsbm1462-bib-0165] Hers, H. (1964). Glycogen storage disease In Advances in metabolic disorders (Vol. 1, pp. 1–44). Elsevier. 10.1016/b978-1-4831-6748-0.50006-9 14171618

[wsbm1462-bib-0166] Heslop, L. , Morgan, J. , & Partridge, T. (2000). Evidence for a myogenic stem cell that is exhausted in dystrophic muscle. Journal of Cell Science, 113(12), 2299–2308. 10825301 10.1242/jcs.113.12.2299

[wsbm1462-bib-0167] Hindi, S. M. , & Kumar, A. (2016). Toll‐like receptor signalling in regenerative myogenesis: Friend and foe. Journal of Pathology, 239(2), 125–128. 26956975 10.1002/path.4714 PMC4957968

[wsbm1462-bib-0168] Hiratsuka, S. , Maru, Y. , Okada, A. , Seiki, M. , Noda, T. , & Shibuya, M. (2001). Involvement of Flt‐1 tyrosine kinase (vascular endothelial growth factor receptor‐1) in pathological angiogenesis. Cancer Research, 61(3), 1207–1213. 11221852

[wsbm1462-bib-0169] Hirsch, N. (2007). Neuromuscular junction in health and disease. British Journal of Anaesthesia, 99(1), 132–138. 17573397 10.1093/bja/aem144

[wsbm1462-bib-0170] Hirst, R. , McCullagh, K. , & Davies, K. (2005). Utrophin upregulation in Duchenne muscular dystrophy. Acta Myologica: Myopathies and Cardiomyopathies, 24(3), 209–216. 16629055

[wsbm1462-bib-0171] Ho, M. S. , Böse, K. , Mokkapati, S. , Nischt, R. , & Smyth, N. (2008). Nidogens—Extracellular matrix linker molecules. Microscopy Research and Technique, 71(5), 387–395. 18219668 10.1002/jemt.20567

[wsbm1462-bib-0172] Hoffman, E. P. , Brown, R. H., Jr. , & Kunkel, L. M. (1987). Dystrophin: The protein product of the Duchenne muscular dystrophy locus. Cell, 51(6), 919–928. 3319190 10.1016/0092-8674(87)90579-4

[wsbm1462-bib-0173] Hoffmann, C. , Moreau, F. , Moes, M. , Luthold, C. , Dieterle, M. , Goretti, E. , … Thomas, C. (2014). Human muscle LIM protein dimerizes along the actin cytoskeleton and cross‐links actin filaments. Molecular and Cellular Biology, 34(16), 3053–3065. 24934443 10.1128/MCB.00651-14 PMC4135597

[wsbm1462-bib-0174] Hoier, B. , & Hellsten, Y. (2014). Exercise‐induced capillary growth in human skeletal muscle and the dynamics of VEGF. Microcirculation, 21(4), 301–314. 24450403 10.1111/micc.12117

[wsbm1462-bib-0175] Hong, Y. H. , Betik, A. C. , & McConell, G. K. (2014). Role of nitric oxide in skeletal muscle glucose uptake during exercise. Experimental Physiology, 99(12), 1569–1573. 25192731 10.1113/expphysiol.2014.079202

[wsbm1462-bib-0176] Horowits, R. , Kempner, E. S. , Bisher, M. E. , & Podolsky, R. J. (1986). A physiological role for titin and nebulin in skeletal muscle. Nature, 323(6084), 160–164. 3755803 10.1038/323160a0

[wsbm1462-bib-0177] Horvath, C. M. (2004). The Jak‐STAT pathway stimulated by interferon γ. Science's STKE, 2004(260), tr8. 10.1126/stke.2602004tr8 15561980

[wsbm1462-bib-0178] Hotamisligil, G. S. (2017a). Foundations of immunometabolism and implications for metabolic health and disease. Immunity, 47(3), 406–420. 28930657 10.1016/j.immuni.2017.08.009 PMC5627521

[wsbm1462-bib-0179] Hotamisligil, G. S. (2017b). Inflammation, metaflammation and immunometabolic disorders. Nature, 542(7640), 177–185. 28179656 10.1038/nature21363

[wsbm1462-bib-0180] Hotamisligil, G. S. , Shargill, N. S. , & Spiegelman, B. M. (1993). Adipose expression of tumor necrosis factor‐alpha: Direct role in obesity‐linked insulin resistance. Science, 259(5091), 87–91. 7678183 10.1126/science.7678183

[wsbm1462-bib-0181] Hu, L.‐Y. R. , Ackermann, M. A. , & Kontrogianni‐Konstantopoulos, A. (2015). The sarcomeric M‐region: A molecular command center for diverse cellular processes. BioMed Research International, 2015, 1–25. 10.1155/2015/714197 PMC4413555 25961035

[wsbm1462-bib-0182] Huang, J. , & Zhu, X. (2016). The molecular mechanisms of calpains action on skeletal muscle atrophy. Physiological Research, 65(4), 547. 26988155 10.33549/physiolres.933087

[wsbm1462-bib-0183] Hughes, B. W. , Kusner, L. L. , & Kaminski, H. J. (2006). Molecular architecture of the neuromuscular junction. Muscle & Nerve, 33(4), 445–461. 16228970 10.1002/mus.20440

[wsbm1462-bib-0184] Humphrey, J. D. , Dufresne, E. R. , & Schwartz, M. A. (2014). Mechanotransduction and extracellular matrix homeostasis. Nature Reviews. Molecular Cell Biology, 15(12), 802–812. 25355505 10.1038/nrm3896 PMC4513363

[wsbm1462-bib-0185] Huxley, H. E. (1957). The double array of filaments in cross‐striated muscle. Journal of Cell Biology, 3(5), 631–648. 10.1083/jcb.3.5.631 PMC2224118 13475381

[wsbm1462-bib-0186] Huxley, H. E. (1969). The mechanism of muscular contraction. Science, 164(3886), 1356–1365. 4181952 10.1126/science.164.3886.1356

[wsbm1462-bib-0187] Huxley, H. E. , & Kress, M. (1985). Crossbridge behaviour during muscle contraction. Journal of Muscle Research & Cell Motility, 6(2), 153–161. 2993356 10.1007/BF00713057

[wsbm1462-bib-0188] Ip, W. E. , Hoshi, N. , Shouval, D. S. , Snapper, S. , & Medzhitov, R. (2017). Anti‐inflammatory effect of IL‐10 mediated by metabolic reprogramming of macrophages. Science, 356(6337), 513–519. 28473584 10.1126/science.aal3535 PMC6260791

[wsbm1462-bib-0189] Jackman, R. W. , Cornwell, E. W. , Wu, C. , & Kandarian, S. C. (2013). Nuclear factor‐κB signalling and transcriptional regulation in skeletal muscle atrophy. Experimental Physiology, 98(1), 19–24. 22848079 10.1113/expphysiol.2011.063321 PMC3505235

[wsbm1462-bib-0190] Jackman, R. W. , & Kandarian, S. C. (2004). The molecular basis of skeletal muscle atrophy. American Journal of Physiology: Cell Physiology, 287(4), C834–C843. 10.1152/ajpcell.00579.2003 15355854

[wsbm1462-bib-0191] Jackson, W. F. (2005). Potassium channels in the peripheral microcirculation. Microcirculation, 12(1), 113–127. 15804979 10.1080/10739680590896072 PMC1405752

[wsbm1462-bib-0192] Jackson, W. F. (2018). KV channels and the regulation of vascular smooth muscle tone. Microcirculation, 25(1), e12421 10.1111/micc.12421 PMC5760307 28985443

[wsbm1462-bib-0193] Jäger, S. , Handschin, C. , Pierre, J. S. , & Spiegelman, B. M. (2007). AMP‐activated protein kinase (AMPK) action in skeletal muscle via direct phosphorylation of PGC‐1α. Proceedings of the National Academy of Sciences, 104(29), 12017–12022. 10.1073/pnas.0705070104 PMC1924552 17609368

[wsbm1462-bib-0194] Jain, R. K. (2005). Normalization of tumor vasculature: An emerging concept in antiangiogenic therapy. Science, 307(5706), 58–62. 15637262 10.1126/science.1104819

[wsbm1462-bib-0195] Jaka, O. , Casas‐Fraile, L. , de Munain, A. L. , & Sáenz, A. (2015). Costamere proteins and their involvement in myopathic processes. Expert Reviews in Molecular Medicine, 17, e12. 26088790 10.1017/erm.2015.9

[wsbm1462-bib-0196] Jeppesen, J. , & Kiens, B. (2012). Regulation and limitations to fatty acid oxidation during exercise. Journal of Physiology, 590(5), 1059–1068. 22271865 10.1113/jphysiol.2011.225011 PMC3381814

[wsbm1462-bib-0197] Jeukendrup, A. E. (2004). Carbohydrate intake during exercise and performance. Nutrition, 20(7–8), 669–677. 15212750 10.1016/j.nut.2004.04.017

[wsbm1462-bib-0198] Jha, A. K. , Huang, S. C.‐C. , Sergushichev, A. , Lampropoulou, V. , Ivanova, Y. , Loginicheva, E. , … Artyomov, M. N. (2015). Network integration of parallel metabolic and transcriptional data reveals metabolic modules that regulate macrophage polarization. Immunity, 42(3), 419–430. 25786174 10.1016/j.immuni.2015.02.005

[wsbm1462-bib-0199] Jiang, C. , Wen, Y. , Kuroda, K. , Hannon, K. , Rudnicki, M. A. , & Kuang, S. (2014). Notch signaling deficiency underlies age‐dependent depletion of satellite cells in muscular dystrophy. Disease Models & Mechanisms, 7(8), 997–1004. 24906372 10.1242/dmm.015917 PMC4107328

[wsbm1462-bib-0200] Joe, A. W. , Yi, L. , Natarajan, A. , Le Grand, F. , So, L. , Wang, J ., … Rossi, F. M . (2010). Muscle injury activates resident fibro/adipogenic progenitors that facilitate myogenesis. Nature Cell Biology, 12(2), 153–163. 20081841 10.1038/ncb2015 PMC4580288

[wsbm1462-bib-0201] Jungbluth, H. (2007). Central core disease. Orphanet Journal of Rare Diseases, 2(1), 25. 17504518 10.1186/1750-1172-2-25 PMC1887524

[wsbm1462-bib-0202] Jungbluth, H. , Treves, S. , Zorzato, F. , Sarkozy, A. , Ochala, J. , Sewry, C. , … Muntoni, F. (2018). Congenital myopathies: Disorders of excitation–contraction coupling and muscle contraction. Nature Reviews. Neurology, 14(3), 151–167. 29391587 10.1038/nrneurol.2017.191

[wsbm1462-bib-0203] Jurkat‐Rott, K. , Mitrovic, N. , Hang, C. , Kouzmenkine, A. , Iaizzo, P. , Herzog, J. , … Lehmann‐Horn, F. (2000). Voltage‐sensor sodium channel mutations cause hypokalemic periodic paralysis type 2 by enhanced inactivation and reduced current. Proceedings of the National Academy of Sciences, 97(17), 9549–9554. 10.1073/pnas.97.17.9549 PMC16902 10944223

[wsbm1462-bib-0204] Kawabe, Y. , Wang, Y. X. , McKinnell, I. W. , Bedford, M. T. , & Rudnicki, M. A. (2012). Carm1 regulates Pax7 transcriptional activity through MLL1/2 recruitment during asymmetric satellite stem cell divisions. Cell Stem Cell, 11(3), 333–345. 22863532 10.1016/j.stem.2012.07.001 PMC3438319

[wsbm1462-bib-0205] Kedzierski, R. M. , & Yanagisawa, M. (2001). Endothelin system: The double‐edged sword in health and disease. Annual Review of Pharmacology and Toxicology, 41(1), 851–876. 10.1146/annurev.pharmtox.41.1.851 11264479

[wsbm1462-bib-0206] Khan, I. M. , Perrard, X.‐Y. , Brunner, G. , Lui, H. , Sparks, L. M. , Smith, S. R. , … Ballantyne, C. M. (2015). Intermuscular and perimuscular fat expansion in obesity correlates with skeletal muscle T cell and macrophage infiltration and insulin resistance. International Journal of Obesity, 39(11), 1607–1618. 26041698 10.1038/ijo.2015.104 PMC5007876

[wsbm1462-bib-0207] Khansari, N. , Shakiba, Y. , & Mahmoudi, M. (2009). Chronic inflammation and oxidative stress as a major cause of age‐related diseases and cancer. Recent Patents on Inflammation & Allergy Drug Discovery, 3(1), 73–80. 19149749 10.2174/187221309787158371

[wsbm1462-bib-0208] Kimbell, L. M. , Ohno, K. , Engel, A. G. , & Rotundo, R. L. (2004). C‐terminal and heparin‐binding domains of collagenic tail subunit are both essential for anchoring acetylcholinesterase at the synapse. Journal of Biological Chemistry, 279(12), 10997–11005. 14702351 10.1074/jbc.M305462200

[wsbm1462-bib-0209] Klevanski, M. , Saar, M. , Baumkötter, F. , Weyer, S. W. , Kins, S. , & Müller, U. C. (2014). Differential role of APP and APLPs for neuromuscular synaptic morphology and function. Molecular and Cellular Neuroscience, 61, 201–210. 24998676 10.1016/j.mcn.2014.06.004

[wsbm1462-bib-0210] Kobzik, L. , Reid, M. B. , Bredt, D. S. , & Stamler, J. S. (1994). Nitric oxide in skeletal muscle. Nature, 372(6506), 546–548. 7527495 10.1038/372546a0

[wsbm1462-bib-0211] Komazaki, S. , Ito, K. , Takeshima, H. , & Nakamura, H. (2002). Deficiency of triad formation in developing skeletal muscle cells lacking junctophilin type 1. FEBS Letters, 524(1–3), 225–229. 12135771 10.1016/s0014-5793(02)03042-9

[wsbm1462-bib-0212] Konopka, A. R. , & Harber, M. P. (2014). Skeletal muscle hypertrophy after aerobic exercise training. Exercise and Sport Sciences Reviews, 42(2), 53–61. 24508740 10.1249/JES.0000000000000007 PMC4523889

[wsbm1462-bib-0213] Kontrogianni‐Konstantopoulos, A. , Catino, D. H. , Strong, J. C. , Sutter, S. , Borisov, A. B. , Pumplin, D. W. , … Bloch, R. J . (2006). Obscurin modulates the assembly and organization of sarcomeres and the sarcoplasmic reticulum. FASEB Journal, 20(12), 2102–2111. 17012262 10.1096/fj.06-5761com

[wsbm1462-bib-0214] Kontrogianni‐Konstantopoulos, A. , Jones, E. M. , Van Rossum, D. B. , & Bloch, R. J. (2003). Obscurin is a ligand for small ankyrin 1 in skeletal muscle. Molecular Biology of the Cell, 14(3), 1138–1148. 12631729 10.1091/mbc.E02-07-0411 PMC151585

[wsbm1462-bib-0215] Koopman, R. , Ly, C. H. , & Ryall, J. G. (2014). A metabolic link to skeletal muscle wasting and regeneration. Frontiers in Physiology, 5, 32. 24567722 10.3389/fphys.2014.00032 PMC3909830

[wsbm1462-bib-0216] Korthuis, R. J. (2011). Anatomy of skeletal muscle and its vascular supply (Vol. 3). San Rafael, CA: Morgan & Claypool Life Sciences Retrieved from https://www.ncbi.nlm.nih.gov/books/NBK57140/ 21850766

[wsbm1462-bib-0217] Kozakowska, M. , Pietraszek‐Gremplewicz, K. , Jozkowicz, A. , & Dulak, J. (2015). The role of oxidative stress in skeletal muscle injury and regeneration: Focus on antioxidant enzymes. Journal of Muscle Research and Cell Motility, 36(6), 377–393. 26728750 10.1007/s10974-015-9438-9 PMC4762917

[wsbm1462-bib-0218] Kozel, B. A. , Ciliberto, C. H. , & Mecham, R. P. (2004). Deposition of tropoelastin into the extracellular matrix requires a competent elastic fiber scaffold but not live cells. Matrix Biology, 23(1), 23–34. 15172035 10.1016/j.matbio.2004.02.004

[wsbm1462-bib-0219] Kramer, I. M. (2016). Cholinergic signaling and muscle contraction In Signal transduction (3rd ed., pp. 263–327). Boston, MA: Academic Press 10.1016/B978-0-12-394803-8.00004-8

[wsbm1462-bib-0220] Kuang, S. , Kuroda, K. , Le Grand, F. , & Rudnicki, M. A. (2007). Asymmetric self‐renewal and commitment of satellite stem cells in muscle. Cell, 129(5), 999–1010. 17540178 10.1016/j.cell.2007.03.044 PMC2718740

[wsbm1462-bib-0221] Kudryashova, E. , Kramerova, I. , & Spencer, M. J. (2012). Satellite cell senescence underlies myopathy in a mouse model of limb‐girdle muscular dystrophy 2H. Journal of Clinical Investigation, 122(5), 1764–1776. 22505452 10.1172/JCI59581 PMC3336976

[wsbm1462-bib-0222] Kumar, D. , Shadrach, J. L. , Wagers, A. J. , & Lassar, A. B. (2009). Id3 is a direct transcriptional target of Pax7 in quiescent satellite cells. Molecular Biology of the Cell, 20(14), 3170–3177. 19458195 10.1091/mbc.E08-12-1185 PMC2710837

[wsbm1462-bib-0223] Kunz, W. S. (2001). Control of oxidative phosphorylation in skeletal muscle. Biochimica et Biophysica Acta, 1504(1), 12–19. 11239481 10.1016/s0005-2728(00)00235-8

[wsbm1462-bib-0224] Laing, N. G. , & Nowak, K. J. (2005). When contractile proteins go bad: The sarcomere and skeletal muscle disease. BioEssays, 27(8), 809–822. 16015601 10.1002/bies.20269

[wsbm1462-bib-0225] Lander, A. D. , Kimble, J. , Clevers, H. , Fuchs, E. , Montarras, D. , Buckingham, M. , … Oskarsson, T . (2012). What does the concept of the stem cell niche really mean today? BMC Biology, 10(1), 19. 22405133 10.1186/1741-7007-10-19 PMC3298504

[wsbm1462-bib-0226] LaPier, T. K. (1997). Glucocorticoid‐induced muscle atrophy: The role of exercise in treatment and prevention. Journal of Cardiopulmonary Rehabilitation and Prevention, 17(2), 76–84. 10.1097/00008483-199703000-00002 9101384

[wsbm1462-bib-0227] Lawrence, T. (2009). The nuclear factor NF‐κB pathway in inflammation. Cold Spring Harbor Perspectives in Biology, 1(6), a001651. 20457564 10.1101/cshperspect.a001651 PMC2882124

[wsbm1462-bib-0228] Lecker, S. H. , Jagoe, R. T. , Gilbert, A. , Gomes, M. , Baracos, V. , Bailey, J. , … Goldberg, A. L . (2004). Multiple types of skeletal muscle atrophy involve a common program of changes in gene expression. FASEB Journal, 18(1), 39–51. 14718385 10.1096/fj.03-0610com

[wsbm1462-bib-0229] Lee, H. , Kim, S.‐H. , Lee, J.‐S. , Yang, Y. H. , Nam, J. M. , Kim, B. W. , & Ko, Y. G. (2016). Mitochondrial oxidative phosphorylation complexes exist in the sarcolemma of skeletal muscle. BMB Reports, 49(2), 116–121. 26645635 10.5483/BMBRep.2016.49.2.232 PMC4915115

[wsbm1462-bib-0230] Lee, Y.‐S. , Lehar, A. , Sebald, S. , Liu, M. , Swaggart, K. A. , Talbot, C. C., Jr. , … Lee, S. J. (2015). Muscle hypertrophy induced by myostatin inhibition accelerates degeneration in dysferlinopathy. Human Molecular Genetics, 24(20), 5711–5719. 26206886 10.1093/hmg/ddv288 PMC4581601

[wsbm1462-bib-0231] Lee, Y. S. , Wollam, J. , & Olefsky, J. M. (2018). An integrated view of immunometabolism. Cell, 172(1), 22–40. 29328913 10.1016/j.cell.2017.12.025 PMC8451723

[wsbm1462-bib-0232] Lee‐Young, R. S. , Canny, B. J. , Myers, D. E. , & McConell, G. K. (2009). AMPK activation is fiber type specific in human skeletal muscle: Effects of exercise and short‐term exercise training. Journal of Applied Physiology, 107(1), 283–289. 19359609 10.1152/japplphysiol.91208.2008

[wsbm1462-bib-0233] Lemos, D. R. , Babaeijandaghi, F. , Low, M. , Chang, C. K. , Lee, S. T. , Fiore, D. , … Rossi, F. M . (2015). Nilotinib reduces muscle fibrosis in chronic muscle injury by promoting TNF‐mediated apoptosis of fibro/adipogenic progenitors. Nature Medicine, 21(7), 786. 10.1038/nm.3869 26053624

[wsbm1462-bib-0234] Lepper, C. , & Fan, C. (2010). Inducible lineage tracing of Pax7‐descendant cells reveals embryonic origin of adult satellite cells. Genesis, 48(7), 424–436. 20641127 10.1002/dvg.20630 PMC3113517

[wsbm1462-bib-0235] Leto, D. , & Saltiel, A. R. (2012). Regulation of glucose transport by insulin: Traffic control of GLUT4. Nature Reviews Molecular Cell Biology, 13(6), 383–396. 22617471 10.1038/nrm3351

[wsbm1462-bib-0236] Li, H. , Malhotra, S. , & Kumar, A. (2008). Nuclear factor‐kappa B signaling in skeletal muscle atrophy. Journal of Molecular Medicine, 86(10), 1113–1126. 18574572 10.1007/s00109-008-0373-8 PMC2597184

[wsbm1462-bib-0237] Li, Y. , Li, J. , Zhu, J. , Sun, B. , Branca, M. , Tang, Y. , … Huard, J. (2007). Decorin gene transfer promotes muscle cell differentiation and muscle regeneration. Molecular Therapy, 15(9), 1616–1622. 17609657 10.1038/sj.mt.6300250

[wsbm1462-bib-0238] Li, Y.‐P. (2003). TNF‐α is a mitogen in skeletal muscle. American Journal of Physiology: Cell Physiology, 285(2), C370–C376. 12711593 10.1152/ajpcell.00453.2002

[wsbm1462-bib-0239] Li, Y.‐P. , Chen, Y. , John, J. , Moylan, J. , Jin, B. , Mann, D. L. , & Reid, M. B. (2005). TNF‐α acts via p38 MAPK to stimulate expression of the ubiquitin ligase atrogin1/MAFbx in skeletal muscle. FASEB Journal, 19(3), 362–370. 15746179 10.1096/fj.04-2364com PMC3099533

[wsbm1462-bib-0240] Li, Z. B. , Kollias, H. D. , & Wagner, K. R. (2008). Myostatin directly regulates skeletal muscle fibrosis. Journal of Biological Chemistry, 283(28), 19371–19378. 18453534 10.1074/jbc.M802585200 PMC2443655

[wsbm1462-bib-0241] Liang, H. , Hussey, S. E. , Sanchez‐Avila, A. , Tantiwong, P. , & Musi, N. (2013). Effect of lipopolysaccharide on inflammation and insulin action in human muscle. PLoS One, 8(5), e63983. 23704966 10.1371/journal.pone.0063983 PMC3660322

[wsbm1462-bib-0242] Liang, H. , & Ward, W. F. (2006). PGC‐1α: A key regulator of energy metabolism. Advances in Physiology Education, 30(4), 145–151. 17108241 10.1152/advan.00052.2006

[wsbm1462-bib-0243] Liang, W.‐C. , & Nishino, I. (2015). Limb‐girdle muscular dystrophy In R. N. Rosenberg & J. M. Pascual (Eds.), Rosenberg's molecular and genetic basis of neurological and psychiatric disease (5th ed., pp. 1113–1120). San Diego, CA: Elsevier.

[wsbm1462-bib-0244] Lieber, R. L. (2009). Skeletal muscle structure, function, and plasticity. Baltimore, MD: Lippincott Williams & Wilkins.

[wsbm1462-bib-0245] Lieber, R. L. , & Friden, J. (2000). Functional and clinical significance of skeletal muscle architecture. Muscle & Nerve, 23(11), 1647–1666. 11054744 10.1002/1097-4598(200011)23:11<1647::aid-mus1>3.0.co;2-m

[wsbm1462-bib-0246] Lim, Y.‐H. , Kwon, D.‐H. , Kim, J. , Park, W. J. , Kook, H. , & Kim, Y.‐K. (2018). Identification of long noncoding RNAs involved in muscle differentiation. PLoS One, 13(3), e0193898. 29499054 10.1371/journal.pone.0193898 PMC5834194

[wsbm1462-bib-0247] Lin, J. , Wu, H. , Tarr, P. T. , Zhang, C. Y. , Wu, Z. , Boss, O. , … Spiegelman, B. M. (2002). Transcriptional co‐activator PGC‐1α drives the formation of slow‐twitch muscle fibres. Nature, 418(6899), 797–801. 12181572 10.1038/nature00904

[wsbm1462-bib-0248] Linke, W. A. (2018). Titin gene and protein functions in passive and active muscle. Annual Review of Physiology, 80, 389–411. 10.1146/annurev-physiol-021317-121234 29131758

[wsbm1462-bib-0249] Linnemann, A. , van der Ven, P. F. , Vakeel, P. , Albinus, B. , Simonis, D. , Bendas, G. , … Fürst, D. O. (2010). The sarcomeric Z‐disc component myopodin is a multiadapter protein that interacts with filamin and α‐actinin. European Journal of Cell Biology, 89(9), 681–692. 20554076 10.1016/j.ejcb.2010.04.004

[wsbm1462-bib-0250] Lluís, F. , Perdiguero, E. , Nebreda, A. R. , & Muñoz‐Cánoves, P. (2006). Regulation of skeletal muscle gene expression by p38 MAP kinases. Trends in Cell Biology, 16(1), 36–44. 16325404 10.1016/j.tcb.2005.11.002

[wsbm1462-bib-0251] Logan, C. V. , Cossins, J. , Cruz, P. M. R. , Parry, D. A. , Maxwell, S. , Martínez‐Martínez, P. , … Robb, S . (2015). Congenital myasthenic syndrome type 19 is caused by mutations in COL13A1, encoding the atypical non‐fibrillar collagen type XIII α1 chain. American Journal of Human Genetics, 97(6), 878–885. 26626625 10.1016/j.ajhg.2015.10.017 PMC4678414

[wsbm1462-bib-0252] Loke, J. , & MacLennan, D. H. (1998). Malignant hyperthermia and central core disease: Disorders of Ca^2+^ release channels. American Journal of Medicine, 104(5), 470–486. 9626031 10.1016/s0002-9343(98)00108-9

[wsbm1462-bib-0253] Londhe, P. , & Davie, J. K. (2011). Gamma interferon modulates myogenesis through the major histocompatibility complex class II transactivator, CIITA. Molecular and Cellular Biology, 31(14), 2854–2866. 21576360 10.1128/MCB.05397-11 PMC3133399

[wsbm1462-bib-0254] Lotze, M. T. , Zeh, H. J. , Rubartelli, A. , Sparvero, L. J. , Amoscato, A. A. , Washburn, N. R. , … Billiar, T. (2007). The grateful dead: Damage‐associated molecular pattern molecules and reduction/oxidation regulate immunity. Immunological Reviews, 220(1), 60–81. 17979840 10.1111/j.1600-065X.2007.00579.x

[wsbm1462-bib-0255] Luther, P. K. (2009). The vertebrate muscle Z‐disc: Sarcomere anchor for structure and signalling. Journal of Muscle Research and Cell Motility, 30(5–6), 171–185. 19830582 10.1007/s10974-009-9189-6 PMC2799012

[wsbm1462-bib-0256] Ma, J. F. , Sanchez, B. J. , Hall, D. T. , Tremblay, A. K. , Di Marco, S. , & Gallouzi, I. (2017). STAT3 promotes IFNγ/TNFα‐induced muscle wasting in an NF‐κB‐dependent and IL‐6‐independent manner. EMBO Molecular Medicine, 9(5), 622–637. 28264935 10.15252/emmm.201607052 PMC5412921

[wsbm1462-bib-0257] Madaro, L. , Passafaro, M. , Sala, D. , Etxaniz, U. , Lugarini, F. , Proietti, D. , … Puri, P. L. (2018). Denervation‐activated STAT3–IL‐6 signalling in fibro‐adipogenic progenitors promotes myofibres atrophy and fibrosis. Nature Cell Biology, 20(8), 917–927. 30050118 10.1038/s41556-018-0151-y PMC6145844

[wsbm1462-bib-0258] Maesner, C. C. , Almada, A. E. , & Wagers, A. J. (2016). Established cell surface markers efficiently isolate highly overlapping populations of skeletal muscle satellite cells by fluorescence‐activated cell sorting. Skeletal Muscle, 6(1), 35. 27826411 10.1186/s13395-016-0106-6 PMC5100091

[wsbm1462-bib-0259] Mah, J. K. , Korngut, L. , Dykeman, J. , Day, L. , Pringsheim, T. , & Jette, N. (2014). A systematic review and meta‐analysis on the epidemiology of Duchenne and Becker muscular dystrophy. Neuromuscular Disorders, 24(6), 482–491. 24780148 10.1016/j.nmd.2014.03.008

[wsbm1462-bib-0260] Mann, C. J. , Perdiguero, E. , Kharraz, Y. , Aguilar, S. , Pessina, P. , Serrano, A. L. , & Muñoz‐Cánoves, P. (2011). Aberrant repair and fibrosis development in skeletal muscle. Skeletal Muscle, 1(1), 21–21. 21798099 10.1186/2044-5040-1-21 PMC3156644

[wsbm1462-bib-0261] Marazzi, G. , & Sassoon, D. (2018). FAPs are sensors for skeletal myofibre atrophy. Nature Cell Biology, 20(8), 864–865. 30050116 10.1038/s41556-018-0149-5

[wsbm1462-bib-0262] Marian, A. J. (2008). Genetic determinants of cardiac hypertrophy. Current Opinion in Cardiology, 23(3), 199–205. 18382207 10.1097/HCO.0b013e3282fc27d9 PMC2767266

[wsbm1462-bib-0263] Mason, S. , & Johnson, R. S. (2007). The role of Hif‐1 1 in hypoxic response in the skeletal muscle In Hypoxia and tHe circulation (pp. 229–244). Boston, MA: Springer. 10.1007/978-0-387-75434-5_18 18269201

[wsbm1462-bib-0264] Mathai, A. S. , Bonen, A. , Benton, C. R. , Robinson, D. L. , & Graham, T. E. (2008). Rapid exercise‐induced changes in PGC‐1α mRNA and protein in human skeletal muscle. Journal of Applied Physiology, 105(4), 1098–1105. 18653753 10.1152/japplphysiol.00847.2007

[wsbm1462-bib-0265] Maximov, A. , Tang, J. , Yang, X. , Pang, Z. P. , & Südhof, T. C. (2009). Complexin controls the force transfer from SNARE complexes to membranes in fusion. Science, 323(5913), 516–521. 19164751 10.1126/science.1166505 PMC3235366

[wsbm1462-bib-0266] Mc Mahan, U. (1990). The agrin hypothesis. Cold Spring Harbor Symposia on Quantitative Biology, 55, 407–418. 1966767 10.1101/sqb.1990.055.01.041

[wsbm1462-bib-0267] McConell, G. K. , Rattigan, S. , Lee‐Young, R. S. , Wadley, G. D. , & Merry, T. L. (2012). Skeletal muscle nitric oxide signaling and exercise: A focus on glucose metabolism. American Journal of Physiology‐Endocrinology and Metabolism, 303(3), E301–E307. 22550064 10.1152/ajpendo.00667.2011

[wsbm1462-bib-0268] McCroskery, S. , Thomas, M. , Maxwell, L. , Sharma, M. , & Kambadur, R. (2003). Myostatin negatively regulates satellite cell activation and self‐renewal. Journal of Cell Biology, 162(6), 1135–1147. 12963705 10.1083/jcb.200207056 PMC2172861

[wsbm1462-bib-0269] McGee, S. L. , Howlett, K. F. , Starkie, R. L. , Cameron‐Smith, D. , Kemp, B. E. , & Hargreaves, M. (2003). Exercise increases nuclear AMPK α2 in human skeletal muscle. Diabetes, 52(4), 926–928. 12663462 10.2337/diabetes.52.4.926

[wsbm1462-bib-0270] Mendias, C. L. , Gumucio, J. P. , Davis, M. E. , Bromley, C. W. , Davis, C. S. , & Brooks, S. V. (2012). Transforming growth factor‐beta induces skeletal muscle atrophy and fibrosis through the induction of atrogin‐1 and scleraxis. Muscle & Nerve, 45(1), 55–59. 22190307 10.1002/mus.22232 PMC3245632

[wsbm1462-bib-0271] Meng, S.‐J. , & Yu, L.‐J. (2010). Oxidative stress, molecular inflammation and sarcopenia. International Journal of Molecular Sciences, 11(4), 1509–1526. 20480032 10.3390/ijms11041509 PMC2871128

[wsbm1462-bib-0272] Millay, D. P. , O'Rourke, J. R. , Sutherland, L. B. , Bezprozvannaya, S. , Shelton, J. M. , Bassel‐Duby, R. , & Olson, E. N. (2013). Myomaker is a membrane activator of myoblast fusion and muscle formation. Nature, 499(7458), 301–305. 23868259 10.1038/nature12343 PMC3739301

[wsbm1462-bib-0273] Millay, D. P. , Sutherland, L. B. , Bassel‐Duby, R. , & Olson, E. N. (2014). Myomaker is essential for muscle regeneration. Genes & Development, 28(15), 1641–1646. 25085416 10.1101/gad.247205.114 PMC4117939

[wsbm1462-bib-0274] Minetti, G. C. , Feige, J. N. , Bombard, F. , Heier, A. , Morvan, F. , Nurnberg, B. , … Fornaro, M. (2014). Gαi2 signaling is required for skeletal muscle growth, regeneration, and satellite cell proliferation and differentiation. Molecular and Cellular Biology, 34(4), 619–630. 24298018 10.1128/MCB.00957-13 PMC3911486

[wsbm1462-bib-0275] Minetti, G. C. , Feige, J. N. , Rosenstiel, A. , Bombard, F. , Meier, V. , Werner, A. , … Fornaro, M. (2011). Gαi2 signaling promotes skeletal muscle hypertrophy, myoblast differentiation, and muscle regeneration. Science Signaling, 4(201), ra80–ra80. 22126963 10.1126/scisignal.2002038

[wsbm1462-bib-0276] Moretti, I. , Ciciliot, S. , Dyar, K. A. , Abraham, R. , Murgia, M. , Agatea, L. , … Schiaffino, S. (2016). MRF4 negatively regulates adult skeletal muscle growth by repressing MEF2 activity. Nature Communications, 7, 12397. 10.1038/ncomms12397 PMC4976255 27484840

[wsbm1462-bib-0277] Morgan, M. J. , & Liu, Z. (2011). Crosstalk of reactive oxygen species and NF‐κB signaling. Cell Research, 21(1), 103–115. 21187859 10.1038/cr.2010.178 PMC3193400

[wsbm1462-bib-0278] Mounier, R. , Chrétien, F. , & Chazaud, B. (2011). Blood vessels and the satellite cell niche In Current topics in developmental biology (Vol. 96, pp. 121–138). San Diego, CA: Elsevier. 21621069 10.1016/B978-0-12-385940-2.00005-X

[wsbm1462-bib-0279] Mounier, R. , Lantier, L. , Leclerc, J. , Sotiropoulos, A. , Pende, M. , Daegelen, D. , … Viollet, B. (2009). Important role for AMPKa1 in limiting skeletal muscle cell hypertrophy. FASEB Journal, 23(7), 2264–2273. 19237506 10.1096/fj.08-119057

[wsbm1462-bib-0280] Mourkioti, F. , & Rosenthal, N. (2008). NF‐κB signaling in skeletal muscle: Prospects for intervention in muscle diseases. Journal of Molecular Medicine, 86(7), 747–759. 18246321 10.1007/s00109-008-0308-4 PMC2480606

[wsbm1462-bib-0281] Moylan, J. S. , Smith, J. D. , Chambers, M. A. , McLoughlin, T. J. , & Reid, M. B. (2008). TNF induction of atrogin‐1/MAFbx mRNA depends on Foxo4 expression but not AKT‐Foxo1/3 signaling. American Journal of Physiology: Cell Physiology, 295(4), C986–C993. 10.1152/ajpcell.00041.2008 18701653 PMC2575831

[wsbm1462-bib-0282] Mukund, K. , Mathewson, M. , Minamoto, V. , Ward, S. R. , Subramaniam, S. , & Lieber, R. L. (2014). Systems analysis of transcriptional data provides insights into muscle's biological response to Botulinum toxin. Muscle & Nerve, 50(5), 744–758. 24536034 10.1002/mus.24211 PMC4136975

[wsbm1462-bib-0283] Mukund, K. , & Subramaniam, S. (2015). Dysregulated mechanisms underlying Duchenne muscular dystrophy from co‐expression network preservation analysis. BMC Research Notes, 8(1), 182. 25935398 10.1186/s13104-015-1141-9 PMC4424514

[wsbm1462-bib-0284] Mukund, K. , & Subramaniam, S. (2017). Co‐expression network approach reveals functional similarities among diseases affecting human skeletal muscle. Frontiers in Physiology, 8, 980. 29249983 10.3389/fphys.2017.00980 PMC5717538

[wsbm1462-bib-0285] Mukund, K. , Ward, S. R. , Lieber, R. L. , & Subramaniam, S. (2017). Co‐expression network approach to studying the effects of Botulinum Neurotoxin‐A. IEEE/ACM Transactions on Computational Biology and Bioinformatics, 15(6), 2009–2016. 10.1109/TCBB.2017.2763949 29053464

[wsbm1462-bib-0286] Muñoz‐Cánoves, P. , Scheele, C. , Pedersen, B. K. , & Serrano, A. L. (2013). Interleukin‐6 myokine signaling in skeletal muscle: A double‐edged sword? FEBS Journal, 280(17), 4131–4148. 23663276 10.1111/febs.12338 PMC4163639

[wsbm1462-bib-0287] Muñoz‐Cánoves, P. , & Serrano, A. L. (2015). Macrophages decide between regeneration and fibrosis in muscle. Trends in Endocrinology & Metabolism, 26(9), 449–450. 26250053 10.1016/j.tem.2015.07.005

[wsbm1462-bib-0288] Muntoni, F. , & Voit, T. (2004). The congenital muscular dystrophies in 2004: A century of exciting progress. Neuromuscular Disorders, 14(10), 635–649. 15351421 10.1016/j.nmd.2004.06.009

[wsbm1462-bib-0289] Muoio, D. M. , & Koves, T. R. (2007). Skeletal muscle adaptation to fatty acid depends on coordinated actions of the PPARs and PGC1α: Implications for metabolic disease. Applied Physiology, Nutrition, and Metabolism, 32(5), 874–883. 10.1139/H07-083 18059612

[wsbm1462-bib-0290] Murphy, R. M. (2010). Calpains, skeletal muscle function and exercise. Clinical and Experimental Pharmacology and Physiology, 37(3), 385–391. 19793101 10.1111/j.1440-1681.2009.05310.x

[wsbm1462-bib-0291] Murphy, S. , & Ohlendieck, K. (2016). The extracellular matrix complexome from skeletal muscle In Composition and function of the extracellular matrix in the human body (pp. 69–92).

[wsbm1462-bib-0292] Musi, N. , & Goodyear, L. (2003). AMP‐activated protein kinase and muscle glucose uptake. Acta Physiologica, 178(4), 337–345. 10.1046/j.1365-201X.2003.01168.x 12864738

[wsbm1462-bib-0293] Nakamura, K. , Koga, Y. , Sakai, H. , Homma, K. , & Ikebe, M. (2007). cGMP‐dependent relaxation of smooth muscle is coupled with the change in the phosphorylation of myosin phosphatase. Circulation Research, 101(7), 712–722. 17673671 10.1161/CIRCRESAHA.107.153981

[wsbm1462-bib-0294] Nance, J. R. , Dowling, J. J. , Gibbs, E. M. , & Bönnemann, C. G. (2012). Congenital myopathies: An update. Current Neurology and Neuroscience Reports, 12(2), 165–174. 22392505 10.1007/s11910-012-0255-x PMC4491488

[wsbm1462-bib-0295] Nandadasa, S. , Foulcer, S. , & Apte, S. S. (2014). The multiple, complex roles of versican and its proteolytic turnover by ADAMTS proteases during embryogenesis. Matrix Biology, 35, 34–41. 24444773 10.1016/j.matbio.2014.01.005 PMC5525047

[wsbm1462-bib-0296] Natsuga, K. , Nishie, W. , Shinkuma, S. , Arita, K. , Nakamura, H. , Ohyama, M. , … Shimizu, H. (2010). Plectin deficiency leads to both muscular dystrophy and pyloric atresia in epidermolysis bullosa simplex. Human Mutation, 31(10), E1687–E1698. 20665883 10.1002/humu.21330 PMC3023027

[wsbm1462-bib-0297] Nazio, F. , & Cecconi, F. (2013). mTOR, AMBRA1, and autophagy: An intricate relationship. Cell Cycle, 12(16), 2524–2525. 23907135 10.4161/cc.25835 PMC3865034

[wsbm1462-bib-0298] Nelson, B. R. , Makarewich, C. A. , Anderson, D. M. , Winders, B. R. , Troupes, C. D. , Wu, F. , … Olson, E. N. (2016). A peptide encoded by a transcript annotated as long noncoding RNA enhances SERCA activity in muscle. Science, 351(6270), 271–275. 26816378 10.1126/science.aad4076 PMC4892890

[wsbm1462-bib-0299] Newsholme, P. , Cruzat, V. F. , Keane, K. N. , Carlessi, R. , & de Bittencourt, P. I. H. (2016). Molecular mechanisms of ROS production and oxidative stress in diabetes. Biochemical Journal, 473(24), 4527–4550. 27941030 10.1042/BCJ20160503C

[wsbm1462-bib-0300] Nie, M. , Deng, Z.‐L. , Liu, J. , & Wang, D.‐Z. (2015). Noncoding RNAs, emerging regulators of skeletal muscle development and diseases. BioMed Research International, 2015, 1–17. 10.1155/2015/676575 PMC4516831 26258142

[wsbm1462-bib-0301] Nishi, M. , Komazaki, S. , Kurebayashi, N. , Ogawa, Y. , Noda, T. , Iino, M. , & Takeshima, H. (1999). Abnormal features in skeletal muscle from mice lacking mitsugumin29. Journal of Cell Biology, 147(7), 1473–1480. 10613905 10.1083/jcb.147.7.1473 PMC2174246

[wsbm1462-bib-0302] Nishimune, H. (2012). Molecular mechanism of active zone organization at vertebrate neuromuscular junctions. Molecular Neurobiology, 45(1), 1–16. 22135013 10.1007/s12035-011-8216-y PMC3890249

[wsbm1462-bib-0303] Oldfors, A. (2007). Hereditary myosin myopathies. Neuromuscular Disorders, 17(5), 355–367. 17434305 10.1016/j.nmd.2007.02.008

[wsbm1462-bib-0304] Olguin, H. C. , & Olwin, B. B. (2004). Pax‐7 up‐regulation inhibits myogenesis and cell cycle progression in satellite cells: A potential mechanism for self‐renewal. Developmental Biology, 275(2), 375–388. 15501225 10.1016/j.ydbio.2004.08.015 PMC3322464

[wsbm1462-bib-0305] O'Loghlen, A. , Perez‐Morgado, M. , Salinas, M. , & Martin, M. (2006). N‐acetyl‐cysteine abolishes hydrogen peroxide‐induced modification of eukaryotic initiation factor 4F activity via distinct signalling pathways. Cellular Signalling, 18(1), 21–31. 15907373 10.1016/j.cellsig.2005.03.013

[wsbm1462-bib-0306] Olsson, A.‐K. , Dimberg, A. , Kreuger, J. , & Claesson‐Welsh, L. (2006). VEGF receptor signalling? In control of vascular function. Nature Reviews Molecular Cell Biology, 7(5), 359–371. 16633338 10.1038/nrm1911

[wsbm1462-bib-0307] Özen, H. (2007). Glycogen storage diseases: New perspectives. World Journal of Gastroenterology, 13(18), 2541–2553. 17552001 10.3748/wjg.v13.i18.2541 PMC4146814

[wsbm1462-bib-0308] Pallafacchina, G. , François, S. , Regnault, B. , Czarny, B. , Dive, V. , Cumano, A. , … Buckingham, M. (2010). An adult tissue‐specific stem cell in its niche: A gene profiling analysis of in vivo quiescent and activated muscle satellite cells. Stem Cell Research, 4(2), 77–91. 19962952 10.1016/j.scr.2009.10.003

[wsbm1462-bib-0309] Palmisano, M. G. , Bremner, S. N. , Hornberger, T. A. , Meyer, G. A. , Domenighetti, A. A. , Shah, S. B. , … Lieber, R. L. (2015). Skeletal muscle intermediate filaments form a stress‐transmitting and stress‐signaling network. Journal of Cell Science, 128(2), 219–224. 25413344 10.1242/jcs.142463 PMC4294770

[wsbm1462-bib-0310] Panati, K. , Suneetha, Y. , & Narala, V. (2016). Irisin/FNDC5—An updated review. European Review for Medical and Pharmacological Sciences, 20(4), 689–697. 26957272

[wsbm1462-bib-0311] Papponen, H. , Kaisto, T. , Leinonen, S. , Kaakinen, M. , & Metsikkö, K. (2009). Evidence for γ‐actin as a Z disc component in skeletal myofibers. Experimental Cell Research, 315(2), 218–225. 19013151 10.1016/j.yexcr.2008.10.021

[wsbm1462-bib-0312] Pardo, P. S. , & Boriek, A. M. (2011). The physiological roles of Sirt1 in skeletal muscle. Aging (Albany NY), 3(4), 430–437. 21483036 10.18632/aging.100312 PMC3117458

[wsbm1462-bib-0313] Patan, S. (2004). Vasculogenesis and angiogenesis In Angiogenesis in brain tumors (pp. 3–32). Boston, MA: Springer.

[wsbm1462-bib-0314] Paulin, D. , & Li, Z. (2004). Desmin: A major intermediate filament protein essential for the structural integrity and function of muscle. Experimental Cell Research, 301(1), 1–7. 15501438 10.1016/j.yexcr.2004.08.004

[wsbm1462-bib-0315] Pawlikowski, B. , Orion Vogler, T. , Gadek, K. , & Olwin, B. (2017). Regulation of skeletal muscle stem cells by fibroblast growth factors. Developmental Dynamics, 246, 359–367. 28249356 10.1002/dvdy.24495

[wsbm1462-bib-0316] Pedersen, B. K. (2011). Muscles and their myokines. Journal of Experimental Biology, 214(2), 337–346. 21177953 10.1242/jeb.048074

[wsbm1462-bib-0317] Pedersen, B. K. , Akerstrom, T. C. , Nielsen, A. R. , & Fischer, C. P. (2007). Role of myokines in exercise and metabolism. Journal of Applied Physiology, 103(3), 1093–1098. 17347387 10.1152/japplphysiol.00080.2007

[wsbm1462-bib-0318] Pedersen, B. K. , & Febbraio, M. A. (2008). Muscle as an endocrine organ: Focus on muscle‐derived interleukin‐6. Physiological Reviews, 88(4), 1379–1406. 18923185 10.1152/physrev.90100.2007

[wsbm1462-bib-0319] Pedersen, B. K. , & Febbraio, M. A. (2012). Muscles, exercise and obesity: Skeletal muscle as a secretory organ. Nature Reviews Endocrinology, 8(8), 457–465. 10.1038/nrendo.2012.49 22473333

[wsbm1462-bib-0320] Perakakis, N. , Triantafyllou, G. A. , Fernández‐Real, J. M. , Huh, J. Y. , Park, K. H. , Seufert, J. , & Mantzoros, C. S. (2017). Physiology and role of irisin in glucose homeostasis. Nature Reviews Endocrinology, 13(6), 324–337. 10.1038/nrendo.2016.221 PMC5878942 28211512

[wsbm1462-bib-0321] Percival, J. M. , Anderson, K. N. , Gregorevic, P. , Chamberlain, J. S. , & Froehner, S. C. (2008). Functional deficits in nNOSμ‐deficient skeletal muscle: Myopathy in nNOS knockout mice. PLoS One, 3(10), e3387. 18852886 10.1371/journal.pone.0003387 PMC2559862

[wsbm1462-bib-0322] Periasamy, M. , & Kalyanasundaram, A. (2007). SERCA pump isoforms: Their role in calcium transport and disease. Muscle & Nerve, 35(4), 430–442. 17286271 10.1002/mus.20745

[wsbm1462-bib-0323] Pernigo, S. , Fukuzawa, A. , Beedle, A. E. , Holt, M. , Round, A. , Pandini, A. , … Steiner, R. A . (2017). Binding of myomesin to obscurin‐like‐1 at the muscle M‐band provides a strategy for isoform‐specific mechanical protection. Structure, 25(1), 107–120. 27989621 10.1016/j.str.2016.11.015 PMC5222588

[wsbm1462-bib-0324] Pernigo, S. , Fukuzawa, A. , Pandini, A. , Holt, M. , Kleinjung, J. , Gautel, M. , & Steiner, R. A. (2015). The crystal structure of the human titin: Obscurin complex reveals a conserved yet specific muscle M‐band zipper module. Journal of Molecular Biology, 427(4), 718–736. 10.1016/j.jmb.2014.11.019 25490259

[wsbm1462-bib-0325] Peter, A. K. , Cheng, H. , Ross, R. S. , Knowlton, K. U. , & Chen, J. (2011). The costamere bridges sarcomeres to the sarcolemma in striated muscle. Progress in Pediatric Cardiology, 31(2), 83–88. 24039381 10.1016/j.ppedcard.2011.02.003 PMC3770312

[wsbm1462-bib-0326] Philippou, A. , Maridaki, M. , Theos, A. , & Koutsilieris, M. (2012). Cytokines in muscle damage In Makowski G. S. (Ed.), Advances in clinical chemistry (Vol. 58, pp. 49–87). San Diego, CA: Elsevier 10.1016/B978-0-12-394383-5.00010-2 22950343

[wsbm1462-bib-0327] Pillon, N. J. , & Krook, A. (2017). Innate immune receptors in skeletal muscle metabolism. Experimental Cell Research, 360, 47–54. 28232117 10.1016/j.yexcr.2017.02.035

[wsbm1462-bib-0328] Pirazzini, M. , Rossetto, O. , Eleopra, R. , & Montecucco, C. (2017). Botulinum neurotoxins: Biology, pharmacology, and toxicology. Pharmacological Reviews, 69(2), 200–235. 28356439 10.1124/pr.116.012658 PMC5394922

[wsbm1462-bib-0329] Pouliquin, P. , & Dulhunty, A. F. (2009). Homer and the ryanodine receptor. European Biophysics Journal, 39(1), 91–102. 19513708 10.1007/s00249-009-0494-1

[wsbm1462-bib-0330] Powers, S. K. , Ji, L. L. , Kavazis, A. N. , & Jackson, M. J. (2011). Reactive oxygen species: Impact on skeletal muscle. Comprehensive Physiology, 1(2), 941–969. 10.1002/cphy.c100054 PMC3893116 23737208

[wsbm1462-bib-0331] Powers, S. K. , Kavazis, A. N. , & McClung, J. M. (2007). Oxidative stress and disuse muscle atrophy. Journal of Applied Physiology, 102(6), 2389–2397. 17289908 10.1152/japplphysiol.01202.2006

[wsbm1462-bib-0332] Powers, S. K. , Smuder, A. , & Judge, A. (2012). Oxidative stress and disuse muscle atrophy: Cause or consequence? Current Opinion in Clinical Nutrition and Metabolic Care, 15(3), 240–245. 22466926 10.1097/MCO.0b013e328352b4c2 PMC3893113

[wsbm1462-bib-0333] Pownall, M. E. , Gustafsson, M. K. , & Emerson, C. P., Jr. (2002). Myogenic regulatory factors and the specification of muscle progenitors in vertebrate embryos. Annual Review of Cell and Developmental Biology, 18(1), 747–783. 10.1146/annurev.cellbio.18.012502.105758 12142270

[wsbm1462-bib-0334] Pownall, M. E. , & Isaacs, H. V. (2010). Fgf signalling in vertebrate development (Vol. 1, pp. 1–75). San Rafael, CA: Morgan & Claypool Life Sciences. 21452439

[wsbm1462-bib-0335] Próchnicki, T. , & Latz, E. (2017). Inflammasomes on the crossroads of innate immune recognition and metabolic control. Cell Metabolism, 26(1), 71–93. 28683296 10.1016/j.cmet.2017.06.018

[wsbm1462-bib-0336] Ptáček, L. J. , Tawil, R. , Griggs, R. C. , Engel, A. G. , Layzer, R. B. , Kwieciński, H. , … Bradley, P . (1994). Dihydropyridine receptor mutations cause hypokalemic periodic paralysis. Cell, 77(6), 863–868. 8004673 10.1016/0092-8674(94)90135-x

[wsbm1462-bib-0337] Puri, P. L. , & Sartorelli, V. (2000). Regulation of muscle regulatory factors by DNA‐binding, interacting proteins, and post‐transcriptional modifications. Journal of Cellular Physiology, 185(2), 155–173. 11025438 10.1002/1097-4652(200011)185:2<155::AID-JCP1>3.0.CO;2-Z

[wsbm1462-bib-0338] Quinn, L. S. , & Anderson, B. G. (2011). Interleukin‐15, IL‐15 receptor‐alpha, and obesity: Concordance of laboratory animal and human genetic studies. Journal of Obesity, 2011, 1–8. 10.1155/2011/456347 PMC3092601 21603270

[wsbm1462-bib-0339] Quinn, L. S. , Anderson, B. G. , Strait‐Bodey, L. , Stroud, A. M. , & Argilés, J. M. (2009). Oversecretion of interleukin‐15 from skeletal muscle reduces adiposity. American Journal of Physiology‐Endocrinology and Metabolism, 296(1), E191–E202. 19001550 10.1152/ajpendo.90506.2008 PMC2636988

[wsbm1462-bib-0340] Rafuse, V. F. , Polo‐Parada, L. , & Landmesser, L. T. (2000). Structural and functional alterations of neuromuscular junctions in NCAM‐deficient mice. Journal of Neuroscience, 20(17), 6529–6539. 10964958 10.1523/JNEUROSCI.20-17-06529.2000 PMC6772958

[wsbm1462-bib-0341] Rall, J. (1996). Role of parvalbumin in skeletal muscle relaxation. Physiology, 11(6), 249–255.

[wsbm1462-bib-0342] Ramadasan‐Nair, R. , Gayathri, N. , Mishra, S. , Sunitha, B. , Mythri, R. B. , Nalini, A. , … Bharath, M. M. S. (2014). Mitochondrial alterations and oxidative stress in an acute transient mouse model of muscle degeneration: Implications for muscular dystrophy and related muscle pathologies. Journal of Biological Chemistry, 289(1), 485–509. 10.1074/jbc.M113.493270 24220031 PMC3879571

[wsbm1462-bib-0343] Raschke, S. , & Eckel, J. (2013). Adipo‐myokines: Two sides of the same coin—Mediators of inflammation and mediators of exercise. Mediators of Inflammation, 2013, 1–16. 10.1155/2013/320724 PMC3686148 23861558

[wsbm1462-bib-0344] Ray, I. , Mahata, S. K. , & De, R. K. (2016). Obesity: An immunometabolic perspective. Frontiers in Endocrinology, 7, 157. 28018292 10.3389/fendo.2016.00157 PMC5149556

[wsbm1462-bib-0345] Rebbeck, R. T. , Nitu, F. R. , Rohde, D. , Most, P. , Bers, D. M. , Thomas, D. D. , & Cornea, R. L. (2016). S100A1 protein does not compete with Calmodulin for ryanodine receptor binding but structurally alters the ryanodine receptor Calmodulin complex. Journal of Biological Chemistry, 291(30), 15896–15907. 27226555 10.1074/jbc.M115.713107 PMC4957069

[wsbm1462-bib-0346] Reggiani, C. , & Te Kronnie, T. (2006). RyR isoforms and fibre type‐specific expression of proteins controlling intracellular calcium concentration in skeletal muscles. Journal of Muscle Research & Cell Motility, 27(5–7), 327–335. 16874451 10.1007/s10974-006-9076-3

[wsbm1462-bib-0347] Reimers, C. D. , Fleckenstein, J. L. , Witt, T. N. , Müller‐Felber, W. , & Pongratz, D. E. (1993). Muscular ultrasound in idiopathic inflammatory myopathies of adults. Journal of the Neurological Sciences, 116(1), 82–92. 8509807 10.1016/0022-510x(93)90093-e

[wsbm1462-bib-0348] Reuter, S. , Gupta, S. C. , Chaturvedi, M. M. , & Aggarwal, B. B. (2010). Oxidative stress, inflammation, and cancer: How are they linked? Free Radical Biology and Medicine, 49(11), 1603–1616. 20840865 10.1016/j.freeradbiomed.2010.09.006 PMC2990475

[wsbm1462-bib-0349] Richter, E. A. , & Hargreaves, M. (2013). Exercise, GLUT4, and skeletal muscle glucose uptake. Physiological Reviews, 93(3), 993–1017. 23899560 10.1152/physrev.00038.2012

[wsbm1462-bib-0350] Rinaldi, C. , Haddad, F. , Bodell, P. W. , Qin, A. X. , Jiang, W. , & Baldwin, K. M. (2008). Intergenic bidirectional promoter and cooperative regulation of the IIx and IIb MHC genes in fast skeletal muscle. American Journal of Physiology‐Regulatory, Integrative and Comparative Physiology, 295(1), R208–R218. 10.1152/ajpregu.00134.2008 PMC2494810 18434443

[wsbm1462-bib-0351] Rivero, J.‐L. L. , Talmadge, R. J. , & Edgerton, V. R. (1998). Fibre size and metabolic properties of myosin heavy chain‐based fibre types in rat skeletal muscle. Journal of Muscle Research & Cell Motility, 19(7), 733–742. 9836144 10.1023/a:1005482816442

[wsbm1462-bib-0352] Rizzoli, S. O. , & Betz, W. J. (2005). Synaptic vesicle pools. Nature Reviews. Neuroscience, 6(1), 57–69. 15611727 10.1038/nrn1583

[wsbm1462-bib-0353] Rocheteau, P. , Vinet, M. , & Chretien, F. (2015). Dormancy and quiescence of skeletal muscle stem cells In B. Brand‐Saberi (Ed.), Vertebrate myogenesis (pp. 215–235). Berlin, Heidelberg: Springer. 10.1007/978-3-662-44608-9_10 25344673

[wsbm1462-bib-0354] Rodney, G. G. , Pal, R. , & Abo‐Zahrah, R. (2016). Redox regulation of autophagy in skeletal muscle. Free Radical Biology and Medicine, 98, 103–112. 27184957 10.1016/j.freeradbiomed.2016.05.010 PMC4975974

[wsbm1462-bib-0355] Rodriguez, J. , Vernus, B. , Chelh, I. , Cassar‐Malek, I. , Gabillard, J. C. , Hadj Sassi, A. , … Bonnieu, A. (2014). Myostatin and the skeletal muscle atrophy and hypertrophy signaling pathways. Cellular and Molecular Life Sciences, 71(22), 4361–4371. 25080109 10.1007/s00018-014-1689-x PMC11113773

[wsbm1462-bib-0356] Rogers, R. S. , & Nishimune, H. (2017). The role of laminins in the organization and function of neuromuscular junctions. Matrix Biology, 57, 86–105. 27614294 10.1016/j.matbio.2016.08.008 PMC5328857

[wsbm1462-bib-0357] Romijn, J. A. , Coyle, E. F. , Sidossis, L. S. , Gastaldelli, A. , Horowitz, J. F. , Endert, E. , & Wolfe, R. R . (1993). Regulation of endogenous fat and carbohydrate metabolism in relation to exercise intensity and duration. American Journal of Physiology‐Endocrinology and Metabolism, 265(3), E380–E391. 10.1152/ajpendo.1993.265.3.E380 8214047

[wsbm1462-bib-0358] Rubinstein, N. A. , & Kelly, A. M. (2004). The diversity of muscle fiber types and its origin during development In A. Engel & C. Franzini‐Armstrong (Eds.), Myology: Basic and clinical (3rd ed., pp. 87–101). McGraw‐Hill Retrieved from http://books.google.com/books?id=zP5vQgAACAAJ

[wsbm1462-bib-0359] Sabatier, L. , Chen, D. , Fagotto‐Kaufmann, C. , Hubmacher, D. , McKee, M. D. , Annis, D. S. , … Reinhardt, D. P. (2009). Fibrillin assembly requires fibronectin. Molecular Biology of the Cell, 20(3), 846–858. 19037100 10.1091/mbc.E08-08-0830 PMC2633374

[wsbm1462-bib-0360] Saccone, V. , & Lorenzo, P. P. (2010). Epigenetic regulation of skeletal myogenesis. Organogenesis, 6(1), 48–53. 20592865 10.4161/org.6.1.11293 PMC2861743

[wsbm1462-bib-0361] Saenz, A. , Leturcq, F. , Cobo, A. M. , Poza, J. J. , Ferrer, X. , Otaegui, D. , … Emparanza, J . (2005). LGMD2A: Genotype–phenotype correlations based on a large mutational survey on the calpain 3 gene. Brain, 128(4), 732–742. 15689361 10.1093/brain/awh408

[wsbm1462-bib-0362] Saghizadeh, M. , Ong, J. M. , Garvey, W. T. , Henry, R. R. , & Kern, P. A. (1996). The expression of TNF alpha by human muscle. Relationship to insulin resistance. Journal of Clinical Investigation, 97(4), 1111–1116. 8613535 10.1172/JCI118504 PMC507159

[wsbm1462-bib-0363] Salo, A. M. , Cox, H. , Farndon, P. , Moss, C. , Grindulis, H. , Risteli, M. , … Myllylä, R. (2008). A connective tissue disorder caused by mutations of the lysyl hydroxylase 3 gene. American Journal of Human Genetics, 83(4), 495–503. 18834968 10.1016/j.ajhg.2008.09.004 PMC2561927

[wsbm1462-bib-0364] Sandri, M. , El Meslemani, A. H. , Sandri, C. , Schjerling, P. , Vissing, K. , Andersen, J. L. , … Angelini, C . (2001). Caspase 3 expression correlates with skeletal muscle apoptosis in Duchenne and facioscapulo human muscular dystrophy. A potential target for pharmacological treatment? Journal of Neuropathology & Experimental Neurology, 60(3), 302–312. 11245214 10.1093/jnen/60.3.302

[wsbm1462-bib-0365] Sandri, M. , Sandri, C. , Gilbert, A. , Skurk, C. , Calabria, E. , Picard, A. , … Goldberg, A. L. (2004). Foxo transcription factors induce the atrophy‐related ubiquitin ligase atrogin‐1 and cause skeletal muscle atrophy. Cell, 117(3), 399–412. 15109499 10.1016/s0092-8674(04)00400-3 PMC3619734

[wsbm1462-bib-0366] Sanes, J. R. (2003). The basement membrane/basal lamina of skeletal muscle. Journal of Biological Chemistry, 278(15), 12601–12604. 12556454 10.1074/jbc.R200027200

[wsbm1462-bib-0367] Sanes, J. R. , & Lichtman, J. W. (1999). Development of the vertebrate neuromuscular junction. Annual Review of Neuroscience, 22(1), 389–442. 10.1146/annurev.neuro.22.1.389 10202544

[wsbm1462-bib-0368] Sanes, J. R. , Marshall, L. M. , & McMahan, U. (1978). Reinnervation of muscle fiber basal lamina after removal of myofibers. Differentiation of regenerating axons at original synaptic sites. Journal of Cell Biology, 78(1), 176–198. 307554 10.1083/jcb.78.1.176 PMC2110176

[wsbm1462-bib-0369] Santacatterina, F. , Chamorro, M. , de Arenas, C. N. , Navarro, C. , Martín, M. A. , Cuezva, J. M. , & Sánchez‐Aragó, M. (2015). Quantitative analysis of proteins of metabolism by reverse phase protein microarrays identifies potential biomarkers of rare neuromuscular diseases. Journal of Translational Medicine, 13(1), 65 10.1186/s12967-015-0424-1 25880557 PMC4342896

[wsbm1462-bib-0370] Sarparanta, J. , Blandin, G. , Charton, K. , Vihola, A. , Marchand, S. , Milic, A. , … Udd, B. (2010). Interactions with M‐band titin and calpain 3 link myospryn (CMYA5) to tibial and limb‐girdle muscular dystrophies. Journal of Biological Chemistry, 285(39), 30304–30315. 20634290 10.1074/jbc.M110.108720 PMC2943315

[wsbm1462-bib-0371] Sarrazin, S. , Lamanna, W. C. , & Esko, J. D. (2011). Heparan sulfate proteoglycans. Cold Spring Harbor Perspectives in Biology, 3(7), a004952. 21690215 10.1101/cshperspect.a004952 PMC3119907

[wsbm1462-bib-0372] Sartori, R. , Milan, G. , Patron, M. , Mammucari, C. , Blaauw, B. , Abraham, R. , & Sandri, M. (2009). Smad2 and 3 transcription factors control muscle mass in adulthood. American Journal of Physiology: Cell Physiology, 296(6), C1248–C1257. 19357234 10.1152/ajpcell.00104.2009

[wsbm1462-bib-0373] Sato, T. , Rocancourt, D. , Marques, L. , Thorsteinsdóttir, S. , & Buckingham, M. (2010). A Pax3/Dmrt2/Myf5 regulatory cascade functions at the onset of myogenesis. PLoS Genetics, 6(4), e1000897. 20368965 10.1371/journal.pgen.1000897 PMC2848553

[wsbm1462-bib-0374] Scharner, J. , & Zammit, P. S. (2011). The muscle satellite cell at 50: The formative years. Skeletal Muscle, 1(1), 28. 21849021 10.1186/2044-5040-1-28 PMC3177780

[wsbm1462-bib-0375] Schiaffino, S. , & Mammucari, C. (2011). Regulation of skeletal muscle growth by the IGF1‐Akt/PKB pathway: Insights from genetic models. Skeletal Muscle, 1, 4 10.1186/2044-5040-1-4 21798082 PMC3143906

[wsbm1462-bib-0376] Schiaffino, S. , & Reggiani, C. (2011). Fiber types in mammalian skeletal muscles. Physiological Reviews, 91(4), 1447–1531. 22013216 10.1152/physrev.00031.2010

[wsbm1462-bib-0377] Schmidt, N. , Akaaboune, M. , Gajendran, N. , Martinez‐Pena y Valenzuela, I. , Wakefield, S. , Thurnheer, R. , & Brenner, H. R. (2011). Neuregulin/ErbB regulate neuromuscular junction development by phosphorylation of α‐dystrobrevin. Journal of Cell Biology, 195, 1171–1184. 22184199 10.1083/jcb.201107083 PMC3246897

[wsbm1462-bib-0378] Schnyder, S. , & Handschin, C. (2015). Skeletal muscle as an endocrine organ: PGC‐1α, myokines and exercise. Bone, 80, 115–125. 26453501 10.1016/j.bone.2015.02.008 PMC4657151

[wsbm1462-bib-0379] Schuler, M. , Ali, F. , Chambon, C. , Duteil, D. , Bornert, J. M. , Tardivel, A. , … Metzger, D. (2006). PGC1α expression is controlled in skeletal muscles by PPARβ, whose ablation results in fiber‐type switching, obesity, and type 2 diabetes. Cell Metabolism, 4(5), 407–414. 17084713 10.1016/j.cmet.2006.10.003

[wsbm1462-bib-0380] Schultz, G. S. , & Wysocki, A. (2009). Interactions between extracellular matrix and growth factors in wound healing. Wound Repair and Regeneration, 17(2), 153–162. 19320882 10.1111/j.1524-475X.2009.00466.x

[wsbm1462-bib-0381] Segalés, J. , Perdiguero, E. , & Muñoz‐Cánoves, P. (2016). Regulation of muscle stem cell functions: A focus on the p38 MAPK signaling pathway. Frontiers in Cell and Developmental Biology, 4, 91. 27626031 10.3389/fcell.2016.00091 PMC5003838

[wsbm1462-bib-0382] Selcen, D. (2011). Myofibrillar myopathies. Neuromuscular Disorders, 21(3), 161–171. 21256014 10.1016/j.nmd.2010.12.007 PMC3052736

[wsbm1462-bib-0383] Selcen, D. , & Engel, A. G. (2004). Mutations in myotilin cause myofibrillar myopathy. Neurology, 62(8), 1363–1371. 15111675 10.1212/01.wnl.0000123576.74801.75

[wsbm1462-bib-0384] Serrano, A. L. , & Muñoz‐Cánoves, P. (2010). Regulation and dysregulation of fibrosis in skeletal muscle. Experimental Cell Research, 316(18), 3050–3058. 20570674 10.1016/j.yexcr.2010.05.035

[wsbm1462-bib-0385] Serrano, A. L. , & Muñoz‐Cánoves, P. (2017). Fibrosis development in early‐onset muscular dystrophies: Mechanisms and translational implications. Seminars in Cell & Developmental Biology, 64, 181–190. 27670721 10.1016/j.semcdb.2016.09.013

[wsbm1462-bib-0386] Shea, K. L. , Xiang, W. , LaPorta, V. S. , Licht, J. D. , Keller, C. , Basson, M. A. , & Brack, A. S . (2010). Sprouty1 regulates reversible quiescence of a self‐renewing adult muscle stem cell pool during regeneration. Cell Stem Cell, 6(2), 117–129. 20144785 10.1016/j.stem.2009.12.015 PMC2846417

[wsbm1462-bib-0387] Sheehan, S. M. , & Allen, R. E. (1999). Skeletal muscle satellite cell proliferation in response to members of the fibroblast growth factor family and hepatocyte growth factor. Journal of Cellular Physiology, 181(3), 499–506. 10528236 10.1002/(SICI)1097-4652(199912)181:3<499::AID-JCP14>3.0.CO;2-1

[wsbm1462-bib-0388] Shen, J. , Tareste, D. C. , Paumet, F. , Rothman, J. E. , & Melia, T. J. (2007). Selective activation of cognate SNAREpins by Sec1/Munc18 proteins. Cell, 128(1), 183–195. 17218264 10.1016/j.cell.2006.12.016

[wsbm1462-bib-0389] Shintaku, J. , Peterson, J. M. , Talbert, E. E. , Gu, J. M. , Ladner, K. J. , Williams, D. R. , … Guttridge, D. C. (2016). MyoD regulates skeletal muscle oxidative metabolism cooperatively with alternative NF‐κB. Cell Reports, 17(2), 514–526. 27705798 10.1016/j.celrep.2016.09.010 PMC5059110

[wsbm1462-bib-0390] Sigoillot, S. M. , Bourgeois, F. , Lambergeon, M. , Strochlic, L. , & Legay, C. (2010). ColQ controls postsynaptic differentiation at the neuromuscular junction. Journal of Neuroscience, 30(1), 13–23. 20053883 10.1523/JNEUROSCI.4374-09.2010 PMC6632527

[wsbm1462-bib-0391] Smith, L. R. , Lee, K. S. , Ward, S. R. , Chambers, H. G. , & Lieber, R. L. (2011). Hamstring contractures in children with spastic cerebral palsy result from a stiffer extracellular matrix and increased in vivo sarcomere length. Journal of Physiology, 589(10), 2625–2639. 21486759 10.1113/jphysiol.2010.203364 PMC3115830

[wsbm1462-bib-0392] Smuder, A. J. , Kavazis, A. N. , Hudson, M. B. , Nelson, W. B. , & Powers, S. K. (2010). Oxidation enhances myofibrillar protein degradation via calpain and caspase‐3. Free Radical Biology and Medicine, 49(7), 1152–1160. 20600829 10.1016/j.freeradbiomed.2010.06.025 PMC2930052

[wsbm1462-bib-0393] So, B. , Kim, H.‐J. , Kim, J. , & Song, W. (2014). Exercise‐induced myokines in health and metabolic diseases. Integrative Medicine Research, 3(4), 172–179. 28664094 10.1016/j.imr.2014.09.007 PMC5481763

[wsbm1462-bib-0394] Soreq, H. , & Seidman, S. (2001). Acetylcholinesterase—New roles for an old actor. Nature Reviews Neuroscience, 2(4), 294–302. 11283752 10.1038/35067589

[wsbm1462-bib-0395] Spriet, L. L. (1992). Anaerobic metabolism in human skeletal muscle during short‐term, intense activity. Canadian Journal of Physiology and Pharmacology, 70(1), 157–165. 1581850 10.1139/y92-023

[wsbm1462-bib-0396] Spriet, L. L. (2014). New insights into the interaction of carbohydrate and fat metabolism during exercise. Sports Medicine, 44(1), 87–96. 10.1007/s40279-014-0154-1 PMC4008806 24791920

[wsbm1462-bib-0397] Stamler, J. S. , & Meissner, G. (2001). Physiology of nitric oxide in skeletal muscle. Physiological Reviews, 81(1), 209–237. 11152758 10.1152/physrev.2001.81.1.209

[wsbm1462-bib-0398] Stanford, K. I. , & Goodyear, L. J. (2018). Muscle‐adipose tissue cross talk. Cold Spring Harbor Perspectives in Medicine, 8(8), a029801. 10.1101/cshperspect.a029801 PMC5685935 28507197

[wsbm1462-bib-0399] Stearns‐Reider, K. M. , D'Amore, A. , Beezhold, K. , Rothrauff, B. , Cavalli, L. , Wagner, W. R. , … Ambrosio, F. (2017). Aging of the skeletal muscle extracellular matrix drives a stem cell fibrogenic conversion. Aging Cell, 16(3), 518–528. 28371268 10.1111/acel.12578 PMC5418187

[wsbm1462-bib-0400] Stitt, T. N. , Drujan, D. , Clarke, B. A. , Panaro, F. , Timofeyva, Y. , Kline, W. O. , … Glass, D. J. (2004). The IGF‐1/PI3K/Akt pathway prevents expression of muscle atrophy‐induced ubiquitin ligases by inhibiting FOXO transcription factors. Molecular Cell, 14(3), 395–403. 15125842 10.1016/s1097-2765(04)00211-4

[wsbm1462-bib-0401] Südhof, T. C. (2004). The synaptic vesicle cycle. Annual Review of Neuroscience, 27, 509–547. 10.1146/annurev.neuro.26.041002.131412 15217342

[wsbm1462-bib-0402] Südhof, T. C. , & Rizo, J. (2011). Synaptic vesicle exocytosis. Cold Spring Harbor Perspectives in Biology, 3(12), a005637. 22026965 10.1101/cshperspect.a005637 PMC3225952

[wsbm1462-bib-0403] Sun, S.‐C. (2011). Non‐canonical NF‐κB signaling pathway. Cell Research, 21(1), 71–85. 21173796 10.1038/cr.2010.177 PMC3193406

[wsbm1462-bib-0404] Szule, J. A. , Harlow, M. L. , Jung, J. H. , De‐Miguel, F. F. , Marshall, R. M. , & McMahan, U. J. (2012). Regulation of synaptic vesicle docking by different classes of macromolecules in active zone material. PLoS One, 7(3), e33333. 22438915 10.1371/journal.pone.0033333 PMC3306385

[wsbm1462-bib-0405] Taegtmeyer, H. , Sen, S. , & Vela, D. (2010). Return to the fetal gene program. Annals of the New York Academy of Sciences, 1188(1), 191–198. 20201903 10.1111/j.1749-6632.2009.05100.x PMC3625436

[wsbm1462-bib-0406] Tajbakhsh, S. (2009). Skeletal muscle stem cells in developmental versus regenerative myogenesis. Journal of Internal Medicine, 266(4), 372–389. 19765181 10.1111/j.1365-2796.2009.02158.x

[wsbm1462-bib-0407] Tajbakhsh, S. , & Cossu, G. (1997). Establishing myogenic identity during somitogenesis. Current Opinion in Genetics & Development, 7(5), 634–641. 9388780 10.1016/s0959-437x(97)80011-1

[wsbm1462-bib-0408] Takada, F. , Vander Woude, D. L. , Tong, H.‐Q. , Thompson, T. G. , Watkins, S. C. , Kunkel, L. M. , & Beggs, A. H. (2001). Myozenin: An α‐actinin‐and γ‐filamin‐binding protein of skeletal muscle Z lines. Proceedings of the National Academy of Sciences, 98(4), 1595–1600. 10.1073/pnas.041609698 PMC29302 11171996

[wsbm1462-bib-0409] Takeshima, H. , Komazaki, S. , Nishi, M. , Iino, M. , & Kangawa, K. (2000). Junctophilins: A novel family of junctional membrane complex proteins. Molecular Cell, 6(1), 11–22. 10949023 10.1016/s1097-2765(00)00003-4

[wsbm1462-bib-0410] Takeshima, H. , Shimuta, M. , Komazaki, S. , Kazuhiro, O. H. M. I. , Nishi, M. , Masamitsu, I. I. N. O. , … Kangawa, K . (1998). Mitsugumin29, a novel synaptophysin family member from the triad junction in skeletal muscle. Biochemical Journal, 331(Pt. 1), 317–322. 9512495 10.1042/bj3310317 PMC1219354

[wsbm1462-bib-0411] Tam, C. S. , Power, J. E. , Markovic, T. P. , Yee, C. , Morsch, M. , McLennan, S. V. , & Twigg, S. M . (2015). The effects of high‐fat feeding on physical function and skeletal muscle extracellular matrix. Nutrition & Diabetes, 5(12), e187. 26657013 10.1038/nutd.2015.39 PMC4735053

[wsbm1462-bib-0412] Tammi, R. H. , Passi, A. G. , Rilla, K. , Karousou, E. , Vigetti, D. , Makkonen, K. , & Tammi, M. I. (2011). Transcriptional and post‐translational regulation of hyaluronan synthesis. FEBS Journal, 278(9), 1419–1428. 21362137 10.1111/j.1742-4658.2011.08070.x

[wsbm1462-bib-0413] Tan, P. L. , Shavlakadze, T. , Grounds, M. D. , & Arthur, P. G. (2015). Differential thiol oxidation of the signaling proteins Akt, PTEN or PP2A determines whether Akt phosphorylation is enhanced or inhibited by oxidative stress in C2C12 myotubes derived from skeletal muscle. International Journal of Biochemistry & Cell Biology, 62, 72–79. 25737250 10.1016/j.biocel.2015.02.015

[wsbm1462-bib-0414] Tang, A. H. , & Rando, T. A. (2014). Induction of autophagy supports the bioenergetic demands of quiescent muscle stem cell activation. EMBO Journal, 33(23), 2782–2797. 25316028 10.15252/embj.201488278 PMC4282556

[wsbm1462-bib-0415] Tee, J.‐M. , & Peppelenbosch, M. P. (2010). Anchoring skeletal muscle development and disease: The role of ankyrin repeat domain containing proteins in muscle physiology. Critical Reviews in Biochemistry and Molecular Biology, 45(4), 318–330. 20515317 10.3109/10409238.2010.488217 PMC2942773

[wsbm1462-bib-0416] Terrill, J. R. , Radley‐Crabb, H. G. , Iwasaki, T. , Lemckert, F. A. , Arthur, P. G. , & Grounds, M. D. (2013). Oxidative stress and pathology in muscular dystrophies: Focus on protein thiol oxidation and dysferlinopathies. FEBS Journal, 280(17), 4149–4164. 23332128 10.1111/febs.12142

[wsbm1462-bib-0417] Thomas, M. , Langley, B. , Berry, C. , Sharma, M. , Kirk, S. , Bass, J. , & Kambadur, R. (2000). Myostatin, a negative regulator of muscle growth, functions by inhibiting myoblast proliferation. Journal of Biological Chemistry, 275(51), 40235–40243. 10976104 10.1074/jbc.M004356200

[wsbm1462-bib-0418] Tidball, J. G. (2011). Mechanisms of muscle injury, repair, and regeneration. Comprehensive Physiology. 10.1002/cphy.c100092 23733696

[wsbm1462-bib-0419] Tidball, J. G. , & Spencer, M. J. (2000). Calpains and muscular dystrophies. International Journal of Biochemistry & Cell Biology, 32(1), 1–5. 10661889 10.1016/s1357-2725(99)00095-3

[wsbm1462-bib-0420] Tidball, J. G. , & Villalta, S. A. (2010). Regulatory interactions between muscle and the immune system during muscle regeneration. American Journal of Physiology. Regulatory, Integrative and Comparative Physiology, 298(5), R1173–R1187. 10.1152/ajpregu.00735.2009 PMC2867520 20219869

[wsbm1462-bib-0421] Timpani, C. A. , Hayes, A. , & Rybalka, E. (2015). Revisiting the dystrophin‐ATP connection: How half a century of research still implicates mitochondrial dysfunction in Duchenne muscular dystrophy aetiology. Medical Hypotheses, 85(6), 1021–1033. 26365249 10.1016/j.mehy.2015.08.015

[wsbm1462-bib-0422] Toth, K. G. , McKay, B. R. , De Lisio, M. , Little, J. P. , Tarnopolsky, M. A. , & Parise, G. (2011). IL‐6 induced STAT3 signalling is associated with the proliferation of human muscle satellite cells following acute muscle damage. PLoS One, 6(3), e17392. 21408055 10.1371/journal.pone.0017392 PMC3052298

[wsbm1462-bib-0423] Tripathy, A. , Xu, L. , Mann, G. , & Meissner, G. (1995). Calmodulin activation and inhibition of skeletal muscle Ca^2+^ release channel (ryanodine receptor). Biophysical Journal, 69(1), 106–119. 7669888 10.1016/S0006-3495(95)79880-0 PMC1236229

[wsbm1462-bib-0424] Tsujino, A. , Maertens, C. , Ohno, K. , Shen, X. M. , Fukuda, T. , Harper, C. M. , … Engel, A. G. (2003). Myasthenic syndrome caused by mutation of the SCN4A sodium channel. Proceedings of the National Academy of Sciences, 100(12), 7377–7382. 10.1073/pnas.1230273100 PMC165883 12766226

[wsbm1462-bib-0425] Ueha, S. , Shand, F. H. , & Matsushima, K. (2012). Cellular and molecular mechanisms of chronic inflammation‐associated organ fibrosis. Frontiers in Immunology, 3, 71. 22566952 10.3389/fimmu.2012.00071 PMC3342381

[wsbm1462-bib-0426] Uezumi, A. , Fukada, S. , Yamamoto, N. , Takeda, S. , & Tsuchida, K. (2010). Mesenchymal progenitors distinct from satellite cells contribute to ectopic fat cell formation in skeletal muscle. Nature Cell Biology, 12(2), 143–152. 20081842 10.1038/ncb2014

[wsbm1462-bib-0427] Uezumi, A. , Ikemoto‐Uezumi, M. , & Tsuchida, K. (2014). Roles of nonmyogenic mesenchymal progenitors in pathogenesis and regeneration of skeletal muscle. Frontiers in Physiology, 5, 68. 24605102 10.3389/fphys.2014.00068 PMC3932482

[wsbm1462-bib-0428] Valle, G. , Faulkner, G. , De Antoni, A. , Pacchioni, B. , Pallavicini, A. , Pandolfo, D. , … Lanfranchi, G . (1997). Telethonin, a novel sarcomeric protein of heart and skeletal muscle. FEBS Letters, 415(2), 163–168. 9350988 10.1016/s0014-5793(97)01108-3

[wsbm1462-bib-0429] Van, P. R. , Fontelonga, T. M. , Barraza‐Flores, P. , Sarathy, A. , Nunes, A. M. , & Burkin, D. J. (2017). ECM‐related myopathies and muscular dystrophies: Pros and cons of protein therapies. Comprehensive Physiology, 7(4), 1519–1536. 28915335 10.1002/cphy.c150033

[wsbm1462-bib-0430] van Rooij, E. , Quiat, D. , Johnson, B. A. , Sutherland, L. B. , Qi, X. , Richardson, J. A. , … Olson, E. N. (2009). A family of microRNAs encoded by myosin genes governs myosin expression and muscle performance. Developmental Cell, 17(5), 662–673. 19922871 10.1016/j.devcel.2009.10.013 PMC2796371

[wsbm1462-bib-0431] Vander Heiden, M. G. , Cantley, L. C. , & Thompson, C. B. (2009). Understanding the Warburg effect: The metabolic requirements of cell proliferation. Science, 324(5930), 1029–1033. 19460998 10.1126/science.1160809 PMC2849637

[wsbm1462-bib-0432] Varkey, B. , & Varkey, L. (2003). Muscle hypertrophy in myotonia congenita. Journal of Neurology, Neurosurgery & Psychiatry, 74(3), 338–338. 10.1136/jnnp.74.3.338 PMC1738324 12588919

[wsbm1462-bib-0433] von Maltzahn, J. , Chang, N. C. , Bentzinger, C. F. , & Rudnicki, M. A. (2012). Wnt signaling in myogenesis. Trends in Cell Biology, 22(11), 602–609. 22944199 10.1016/j.tcb.2012.07.008 PMC3479319

[wsbm1462-bib-0434] Wallace, D. C. (2000). Mitochondrial defects in cardiomyopathy and neuromuscular disease. American Heart Journal, 139(2, Suppl. 2), s70–s85. 10.1067/mhj.2000.103934 10650320

[wsbm1462-bib-0435] Wallimann, T. , & Eppenberger, H. M. (1985). Localization and function of M‐line‐bound creatine kinase In J. W. Shay (Ed.), Cell and muscle motility (pp. 239–285). Boston, MA: Springer. 10.1007/978-1-4757-4723-2_8 3888375

[wsbm1462-bib-0436] Wallimann, T. , Schlösser, T. , & Eppenberger, H. M. (1984). Function of M‐line‐bound creatine kinase as intramyofibrillar ATP regenerator at the receiving end of the phosphorylcreatine shuttle in muscle. Journal of Biological Chemistry, 259(8), 5238–5246. 6143755

[wsbm1462-bib-0437] Wanders, R. J. , Ruiter, J. P. , IJlst, L. , Waterham, H. R. , & Houten, S. M. (2010). The enzymology of mitochondrial fatty acid beta‐oxidation and its application to follow‐up analysis of positive neonatal screening results. Journal of Inherited Metabolic Disease, 33(5), 479–494. 20490924 10.1007/s10545-010-9104-8 PMC2946543

[wsbm1462-bib-0438] Wang, C. , Bai, J. , Chang, P. Y. , Chapman, E. R. , & Jackson, M. B. (2006). Synaptotagmin–Ca^2+^ triggers two sequential steps in regulated exocytosis in rat PC12 cells: Fusion pore opening and fusion pore dilation. Journal of Physiology, 570(2), 295–307. 16293646 10.1113/jphysiol.2005.097378 PMC1464313

[wsbm1462-bib-0439] Wang, K. , McClure, J. , & Tu, A. (1979). Titin: Major myofibrillar components of striated muscle. Proceedings of the National Academy of Sciences, 76(8), 3698–3702. 10.1073/pnas.76.8.3698 PMC383900 291034

[wsbm1462-bib-0440] Wang, K. , Wang, C. , Xiao, F. , Wang, H. , & Wu, Z. (2008). JAK2/STAT2/STAT3 are required for myogenic differentiation. Journal of Biological Chemistry, 283(49), 34029–34036. 18835816 10.1074/jbc.M803012200 PMC2662224

[wsbm1462-bib-0441] Wang, Y. , Winters, J. , & Subramaniam, S. (2012). Functional classification of skeletal muscle networks. II. Applications to pathophysiology. Journal of Applied Physiology, 113(12), 1902–1920. 23085957 10.1152/japplphysiol.01515.2011 PMC3544493

[wsbm1462-bib-0442] Wang, Y.‐X. , Zhang, C.‐L. , Yu, R. T. , Cho, H. K. , Nelson, M. C. , Bayuga‐Ocampo, C. R. , … Evans, R. M. (2004). Regulation of muscle fiber type and running endurance by PPARδ. PLoS Biology, 2(10), e294. 15328533 10.1371/journal.pbio.0020294 PMC509410

[wsbm1462-bib-0443] Webb, R. C. (2003). Smooth muscle contraction and relaxation. Advances in Physiology Education, 27(4), 201–206. 14627618 10.1152/advan.00025.2003

[wsbm1462-bib-0444] Wehling, M. , Spencer, M. J. , & Tidball, J. G. (2001). A nitric oxide synthase transgene ameliorates muscular dystrophy in mdx mice. Journal of Cell Biology, 155(1), 123–132. 11581289 10.1083/jcb.200105110 PMC2150800

[wsbm1462-bib-0445] Weinberg, R. A. (1995). The retinoblastoma protein and cell cycle control. Cell, 81(3), 323–330. 7736585 10.1016/0092-8674(95)90385-2

[wsbm1462-bib-0446] Wilkes, M. C. , Mitchell, H. , Penheiter, S. G. , Doré, J. J. , Suzuki, K. , Edens, M. , … Leof, E. B. (2005). Transforming growth factor‐β activation of phosphatidylinositol 3‐kinase is independent of Smad2 and Smad3 and regulates fibroblast responses via p21‐activated kinase‐2. Cancer Research, 65(22), 10431–10440. 16288034 10.1158/0008-5472.CAN-05-1522

[wsbm1462-bib-0447] Williams, A. S. , Kang, L. , & Wasserman, D. H. (2015). The extracellular matrix and insulin resistance. Trends in Endocrinology & Metabolism, 26(7), 357–366. 26059707 10.1016/j.tem.2015.05.006 PMC4490038

[wsbm1462-bib-0448] Williams, R. S. , & Annex, B. H. (2004). Plasticity of myocytes and capillaries: A possible coordinating role for VEGF. Circulation Research, 95(1), 7–8. 15242980 10.1161/01.RES.0000136345.81719.37

[wsbm1462-bib-0449] Wilson, J. M. , Loenneke, J. P. , Jo, E. , Wilson, G. J. , Zourdos, M. C. , & Kim, J.‐S. (2012). The effects of endurance, strength, and power training on muscle fiber type shifting. Journal of Strength and Conditioning Research, 26(6), 1724–1729. 21912291 10.1519/JSC.0b013e318234eb6f

[wsbm1462-bib-0450] Winter, L. , Türk, M. , Harter, P. N. , Mittelbronn, M. , Kornblum, C. , Norwood, F. , … Schröder, R. (2016). Downstream effects of plectin mutations in epidermolysis bullosa simplex with muscular dystrophy. Acta Neuropathologica Communications, 4(1), 44. 27121971 10.1186/s40478-016-0314-7 PMC4847350

[wsbm1462-bib-0451] Wood, S. , & Slater, C. (1998). β‐Spectrin is colocalized with both voltage‐gated sodium channels and ankyrinG at the adult rat neuromuscular junction. Journal of Cell Biology, 140(3), 675–684. 9456326 10.1083/jcb.140.3.675 PMC2140176

[wsbm1462-bib-0452] Wu, C.‐L. , Cornwell, E. W. , Jackman, R. W. , & Kandarian, S. C. (2014). NF‐κB but not FoxO sites in the MuRF1 promoter are required for transcriptional activation in disuse muscle atrophy. American Journal of Physiology: Cell Physiology, 306(8), C762–C767. 24553183 10.1152/ajpcell.00361.2013 PMC3989716

[wsbm1462-bib-0453] Wu, H. , Naya, F. J. , McKinsey, T. A. , Mercer, B. , Shelton, J. M. , Chin, E. R. , … Williams, R. S. (2000). MEF2 responds to multiple calcium‐regulated signals in the control of skeletal muscle fiber type. EMBO Journal, 19(9), 1963–1973. 10790363 10.1093/emboj/19.9.1963 PMC305686

[wsbm1462-bib-0454] Wu, H. , Xiong, W. C. , & Mei, L. (2010). To build a synapse: Signaling pathways in neuromuscular junction assembly. Development, 137(7), 1017–1033. 20215342 10.1242/dev.038711 PMC2835321

[wsbm1462-bib-0455] Wu, L.‐G. , Hamid, E. , Shin, W. , & Chiang, H.‐C. (2014). Exocytosis and endocytosis: Modes, functions, and coupling mechanisms. Annual Review of Physiology, 76, 301–331. 10.1146/annurev-physiol-021113-170305 PMC4880020 24274740

[wsbm1462-bib-0456] Wu, Z. , Puigserver, P. , Andersson, U. , Zhang, C. , Adelmant, G. , Mootha, V. , … Spiegelman, B. M. (1999). Mechanisms controlling mitochondrial biogenesis and respiration through the thermogenic coactivator PGC‐1. Cell, 98(1), 115–124. 10412986 10.1016/S0092-8674(00)80611-X

[wsbm1462-bib-0457] Xiao, S. , & Gräter, F. (2014). Molecular basis of the mechanical hierarchy in myomesin dimers for sarcomere integrity. Biophysical Journal, 107(4), 965–973. 25140432 10.1016/j.bpj.2014.06.043 PMC4142248

[wsbm1462-bib-0458] Yablonka‐Reuveni, Z. , & Anderson, J. E. (2006). Satellite cells from dystrophic (mdx) mice display accelerated differentiation in primary cultures and in isolated myofibers. Developmental Dynamics, 235(1), 203–212. 16258933 10.1002/dvdy.20602

[wsbm1462-bib-0459] Yajima, H. , Motohashi, N. , Ono, Y. , Sato, S. , Ikeda, K. , Masuda, S. , … Kawakami, K. (2010). Six family genes control the proliferation and differentiation of muscle satellite cells. Experimental Cell Research, 316(17), 2932–2944. 20696153 10.1016/j.yexcr.2010.08.001

[wsbm1462-bib-0460] Yamagata, M. , Sanes, J. R. , & Weiner, J. A. (2003). Synaptic adhesion molecules. Current Opinion in Cell Biology, 15(5), 621–632. 14519398 10.1016/s0955-0674(03)00107-8

[wsbm1462-bib-0461] Yamaguchi, M. , Watanabe, Y. , Ohtani, T. , Uezumi, A. , Mikami, N. , Nakamura, M. , … Fukada, S. I. (2015). Calcitonin receptor signaling inhibits muscle stem cells from escaping the quiescent state and the niche. Cell Reports, 13(2), 302–314. 26440893 10.1016/j.celrep.2015.08.083

[wsbm1462-bib-0462] Yamashita, A. , Maeda, K. , & Maéda, Y. (2003). Crystal structure of CapZ: Structural basis for actin filament barbed end capping. EMBO Journal, 22(7), 1529–1538. 12660160 10.1093/emboj/cdg167 PMC152911

[wsbm1462-bib-0463] Ye, J. , Zhang, Y. , Xu, J. , Zhang, Q. , & Zhu, D. (2007). FBXO40, a gene encoding a novel muscle‐specific F‐box protein, is upregulated in denervation‐related muscle atrophy. Gene, 404(1), 53–60. 17928169 10.1016/j.gene.2007.08.020

[wsbm1462-bib-0464] Yeagley, D. , & Lang, C. H. (2010). Endotoxin‐induced IL‐6 promoter activation in skeletal muscle requires an NF‐κB site. International Journal of Interferon, Cytokine and Mediator Research, 2010(2), 9–21. 10.2147/IJICMR.S6690 PMC3713526 23874122

[wsbm1462-bib-0465] Yin, H. , Price, F. , & Rudnicki, M. A. (2013). Satellite cells and the muscle stem cell niche. Physiological Reviews, 93(1), 23–67. 23303905 10.1152/physrev.00043.2011 PMC4073943

[wsbm1462-bib-0466] Yoshida, M. , Minamisawa, S. , Shimura, M. , Komazaki, S. , Kume, H. , Zhang, M. , … Takeshima, H. (2005). Impaired Ca^2+^ store functions in skeletal and cardiac muscle cells from sarcalumenin‐deficient mice. Journal of Biological Chemistry, 280(5), 3500–3506. 15569689 10.1074/jbc.M406618200

[wsbm1462-bib-0467] Yu, J.‐G. , Bonnerud, P. , Eriksson, A. , Stål, P. S. , Tegner, Y. , & Malm, C. (2014). Effects of long term supplementation of anabolic androgen steroids on human skeletal muscle. PLoS One, 9(9), e105330 10.1371/journal.pone.0105330 25207812 PMC4160183

[wsbm1462-bib-0468] Zhang, L. , Kelley, J. , Schmeisser, G. , Kobayashi, Y. M. , & Jones, L. R. (1997). Complex formation between junctin, triadin, calsequestrin, and the ryanodine receptor proteins of the cardiac junctional sarcoplasmic reticulum membrane. Journal of Biological Chemistry, 272(37), 23389–23397. 9287354 10.1074/jbc.272.37.23389

[wsbm1462-bib-0469] Zhang, L. , Kimball, S. R. , Jefferson, L. S. , & Shenberger, J. S. (2009). Hydrogen peroxide impairs insulin‐stimulated assembly of mTORC1. Free Radical Biology and Medicine, 46(11), 1500–1509. 19281842 10.1016/j.freeradbiomed.2009.03.001 PMC2677139

[wsbm1462-bib-0470] Zhang, Q. , Vashisht, A. A. , O'Rourke, J. , Corbel, S. Y. , Moran, R. , Romero, A. , … Sampath, S. C . (2017). The microprotein Minion controls cell fusion and muscle formation. Nature Communications, 8, 15664. 10.1038/ncomms15664 PMC5461507 28569745

[wsbm1462-bib-0471] Zhao, C. , Slevin, J. T. , & Whiteheart, S. W. (2007). Cellular functions of NSF: Not just SNAPs and SNAREs. FEBS Letters, 581(11), 2140–2149. 17397838 10.1016/j.febslet.2007.03.032 PMC1948069

[wsbm1462-bib-0472] Zhao, J. , Brault, J. J. , Schild, A. , Cao, P. , Sandri, M. , Schiaffino, S. , … Goldberg, A. L. (2007). FoxO3 coordinately activates protein degradation by the autophagic/lysosomal and proteasomal pathways in atrophying muscle cells. Cell Metabolism, 6(6), 472–483. 18054316 10.1016/j.cmet.2007.11.004

[wsbm1462-bib-0473] Zhao, Y. , Hu, X. , Liu, Y. , Dong, S. , Wen, Z. , He, W. , … Shi, M. (2017). ROS signaling under metabolic stress: Cross‐talk between AMPK and AKT pathway. Molecular Cancer, 16(1), 79. 28407774 10.1186/s12943-017-0648-1 PMC5390360

[wsbm1462-bib-0474] Zhu, H. , Xiao, F. , Wang, G. , Wei, X. , Jiang, L. , Chen, Y. , … Wu, Z. (2016). STAT3 regulates self‐renewal of adult muscle satellite cells during injury‐induced muscle regeneration. Cell Reports, 16(8), 2102–2115. 27524611 10.1016/j.celrep.2016.07.041

[wsbm1462-bib-0475] Zhu, J. , Li, Y. , Shen, W. , Qiao, C. , Ambrosio, F. , Lavasani, M. , … Huard, J. (2007). Relationships between transforming growth factor‐β1, myostatin, and decorin: Implications for skeletal muscle fibrosis. Journal of Biological Chemistry, 282(35), 25852–25863. 17597062 10.1074/jbc.M704146200

[wsbm1462-bib-0476] Zhu, X. , Hadhazy, M. , Wehling, M. , Tidball, J. G. , & McNally, E. M. (2000). Dominant negative myostatin produces hypertrophy without hyperplasia in muscle. FEBS Letters, 474(1), 71–75. 10828454 10.1016/s0014-5793(00)01570-2

[wsbm1462-bib-0477] Zong, Y. , Zhang, B. , Gu, S. , Lee, K. , Zhou, J. , Yao, G. , … Jin, R. (2012). Structural basis of agrin‐LRP4‐MuSK signaling. Genes & Development, 26(3), 247–258. 22302937 10.1101/gad.180885.111 PMC3278892

[wsbm1462-bib-0478] Zot, A. S. , & Potter, J. D. (1987). Structural aspects of troponin‐tropomyosin regulation of skeletal muscle contraction. Annual Review of Biophysics and Biophysical Chemistry, 16(1), 535–559. 10.1146/annurev.bb.16.060187.002535 2954560

[wsbm1462-bib-0479] Zou, P. , Pinotsis, N. , Lange, S. , Song, Y. H. , Popov, A. , Mavridis, I. , … Wilmanns, M. (2006). Palindromic assembly of the giant muscle protein titin in the sarcomeric Z‐disk. Nature, 439(7073), 229–233. 16407954 10.1038/nature04343

[wsbm1462-bib-0480] Zuber, B. , & Unwin, N. (2013). Structure and superorganization of acetylcholine receptor–rapsyn complexes. Proceedings of the National Academy of Sciences, 110(26), 10622–10627. 10.1073/pnas.1301277110 PMC3696831 23754381

[wsbm1462-bib-0481] Zweier, J. L. , & Talukder, M. H. (2006). The role of oxidants and free radicals in reperfusion injury. Cardiovascular Research, 70(2), 181–190. 16580655 10.1016/j.cardiores.2006.02.025

